# Organophosphorus *S*-adenosyl-*L*-methionine mimetics: synthesis, stability, and substrate properties

**DOI:** 10.3389/fchem.2024.1448747

**Published:** 2024-08-01

**Authors:** Alexander Yu Rudenko, Sofia S. Mariasina, Anastasiia K. Bolikhova, Maxim V. Nikulin, Ratislav M. Ozhiganov, Vasiliy G. Vasil’ev, Yuri A. Ikhalaynen, Anastasia L. Khandazhinskaya, Maxim A. Khomutov, Peter V. Sergiev, Alex R. Khomutov, Vladimir I. Polshakov

**Affiliations:** ^1^ Belozersky Institute of Physico-Chemical Biology, M. V. Lomonosov Moscow State University, Moscow, Russia; ^2^ Faculty of Fundamental Medicine, M. V. Lomonosov Moscow State University, Moscow, Russia; ^3^ Chemistry Department, M. V. Lomonosov Moscow State University, Moscow, Russia; ^4^ Research and Educational Resource Center “Pharmacy”, RUDN University, Moscow, Russia; ^5^ Higher Chemical College RAS, Mendeleev University of Chemical Technology, Moscow, Russia; ^6^ Engelhardt Institute of Molecular Biology, Russian Academy of Sciences, Moscow, Russia

**Keywords:** *S*-adenosyl-L-methionine, SAM analogs, halide methyltransferase, MAT2A, COMT, WBSCR27, AdoMet mimetics

## Abstract

*S*-Adenosyl-l-methionine (SAM)-mediated methylation of biomolecules controls their function and regulates numerous vital intracellular processes. Analogs of SAM with a reporter group in place of the *S*-methyl group are widely used to study these processes. However, many of these analogs are chemically unstable that largely limits their practical application. We have developed a new compound, **SAM-P**_**H**_, which contains an *H-*phosphinic group (-P(O)(H)OH) instead of the SAM carboxylic group. **SAM-P**_**H**_ is significantly more stable than SAM, retains functional activity in catechol-O-methyltransferase and methyltransferase WBSCR27 reactions. The last is associated with Williams–Beuren syndrome. *Rac*-**SAM-P**_**H**_ was synthesized chemically, while (*R,S*)-**SAM-P**_**H**_ and its analogs were prepared enzymatically either from *H*-phosphinic analogs of methionine (Met-P_H_) or *H*-phosphinic analog of *S*-adenosyl-l-homocysteine (**SAH-P**_**H**_) using methionine adenosyltransferase 2A or halide methyltransferases, respectively. **SAH-P**_**H**_ undergoes glycoside bond cleavage in the presence of methylthioadenosine nucleosidase like natural SAH. Thus, **SAM-P**_**H**_ and its analogs are promising new tools for investigating methyltransferases and incorporating reporter groups into their substrates.

## 1 Introduction

*S*-Adenosyl-l-methionine (SAM) is the second most abundant cofactor in living systems after ATP. It is a methyl group donor for SAM-dependent methyltransferases (MTases, E.C. 2.1.1.X), which are enzymes responsible for the methylation of biomolecules. Enzymatic methylation of DNA, RNA, proteins, and small-molecule metabolites is chemo-, regio-, and stereo-selective and is crucial for the functions of biomolecules (Struck et al., 2012; Bennett et al., 2017; Ospina et al., 2022). Many vital intracellular processes including the regulation of cancer-related gene expression through promoter hypermethylation (Ehrlich, 2019), control of protein biosynthesis *via* rRNA methylation (Sergiev et al., 2018) and multiple tRNA methylation (Hori, 2014), and histone methylation in epigenetics (Zhang et al., 2021) depend on the proper methylation of biomolecules. Small-molecule methylation is important in regulatory processes, e.g., noradrenaline to adrenaline conversion catalyzed by the phenylethanolamine *N*-methyltransferase (Ubuka, 2021). All these regulatory processes rely on a single methyl group insertion, dramatically altering the properties and functions of the biomolecules.

One of the modern approaches for studying methyltransferase reactions is the enzymatic derivatization of biomolecules using SAM analogs with a reporter group instead of a methyl group. Some of these SAM analogs are the substrates of methyltransferases. For example, a clickable propargylated SAM analog (ProSeAM) was used for the modification of cytosine bases of mRNA (Hartstock et al., 2023) and other substrates (Sohtome et al., 2021; Cornelissen et al., 2023). However, the widespread use of most of SAM analogs is hindered by their intrinsic chemical instability. Natural SAM and its sulfonium-substituted derivatives degrade rapidly under physiological conditions, forming 5′-deoxy-5′-methylthioadenosine (MTA) and homoserine lactone (Parks and Schlenk, 1958b; Wu et al., 1983). Half-life of SAM is approximately 16 h at pH 8 (Huber et al., 2016).

Certain bioisosteric substitutions in the SAM molecule are known to increase its stability in key decomposition reactions (depurination, intramolecular cyclization, and sulfonium epimerization) (Huber et al., 2020; Rudenko et al., 2022; Neti et al., 2023; Okuda et al., 2023). For example, replacing the carboxyl group of SAM with a tetrazole ring significantly improved the stability of this SAM analog (half-life is 83 h at pH 8 instead of 16 h for SAM (Huber et al., 2016)) and did not affect its substrate properties, at least in the carminomycin 4-O-methyltransferase reaction (Huber et al., 2016). Substitution of the sulfur atom of SAM with selenium also increased the stability of SAM and its analogs. Thus, fluoroethyl Se-adenosyl-L-selenomethionine was the substrate of some methyltransferases and fluoroethylated several O-, N-, S-, and C-nucleophiles (Yu et al., 2024).

The chemical instability of SAM and its functionally active mimetics is also a significant drawback when scaling up SAM-dependent reactions (Tang et al., 2021b; Schneider et al., 2021; Mohr et al., 2023). During the methylation reaction, SAM is converted to SAH, which acts as a competitive inhibitor of MTase (Ueland, 1982; Mariasina et al., 2020). To regenerate SAM from SAH, various enzymatic methods have been developed that significantly reduce the required amount of SAM in these reactions (Popadić et al., 2021; Gericke et al., 2023). For example, methyl iodide in the presence of halide methyltransferase (HMT) has been used to regenerate SAM from SAH, with the catalytic cofactor recycling up to 580 times (Liao and Seebeck, 2019). Clearly, SAM stability is an important factor in these enzymatic cascade systems (Mordhorst et al., 2017; Hoffmann et al., 2023).

Not all stable SAM analogs, including those with reporter groups in the sulfonium center, are recognized by target methyltransferases to the required extent. Thus, the range of chemically stable SAM analogs needs to be expanded. In this work, the racemic *H*-phosphinic analog of SAM (*rac-* **SAM-P**_**H**_) was synthesized chemically, and its increased chemical stability was demonstrated. First, enzymatic syntheses of (*R,S*)-**SAM-P**_**H**_ having the same stereoconfiguration as natural SAM were performed starting either from *H*-phosphinic analogs of methionine (**Met-P**_**H**_) or *S*-adenosylhomocysteine (**SAH-P**_**H**_) and using methionine adenosyltransferase or halide methyltransferase, respectively. (*R,S*)-**SAM-P**_**H**_ turned to be a methyl group donor in the catechol-*O*-methyltransferase reaction, and NMR studies of the (*R,S*)-**SAM-P**_**H**_ interaction with the active site of methyltransferase WBSCR27, associated with the Williams–Beuren syndrome, confirmed that the binding of this analog is similar to that of natural SAM. Thus, **SAM-P**_**H**_ is a novel functionally active mimetic of SAM and a scaffold for the synthesis of chemically stable S-substituted derivatives with reporter groups allowing labelling of the substrates of methyltransferases.

## 2 Results

### 2.1 Chemical synthesis of phosphorus analogs of methionine, SAH, and SAM

**FIGURE 1:**
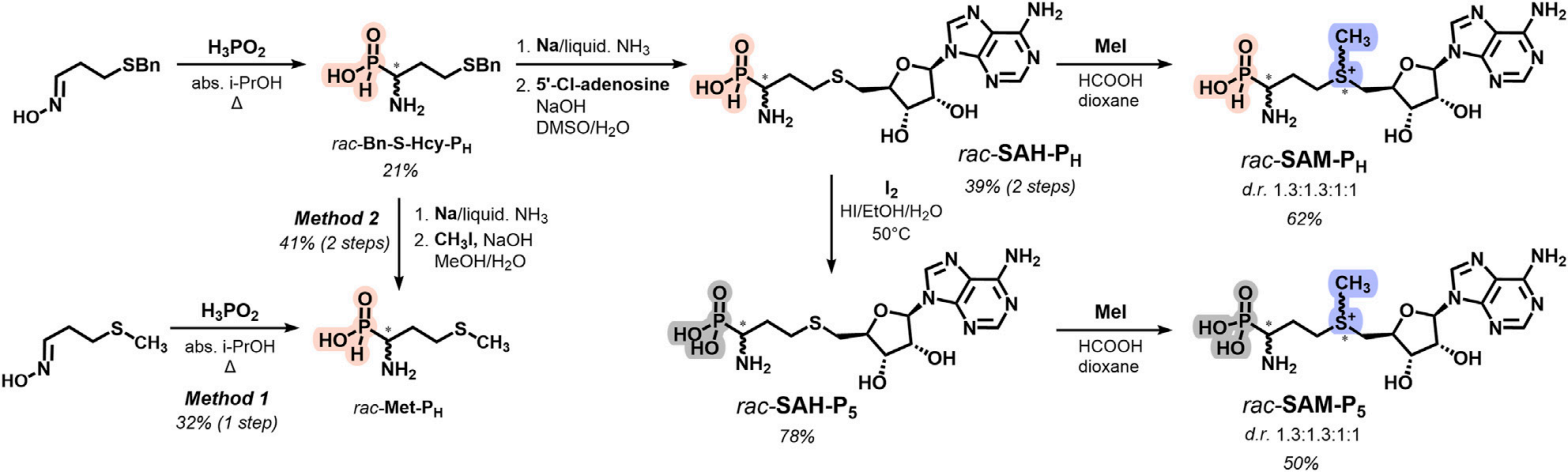
Overview and synthetic route for *rac-* **Met-P**_**H**_, *rac-* **SAM-P**_**H**_, and *rac-* **SAM-P**_**5**_.

Racemic *H*-phosphinic analogs of methionine **Met-P**_**H**_ (Figure 1, Supplementary Scheme S1) were obtained in gram-scale by reacting oximes with hypophosphorous acid in boiling alcohols and isolated with good or moderate yields by ion exchange chromatography or crystallization (see Experimental section).This one-step synthesis method, developed earlier (Zhukov et al., 1999), uses readily available starting materials, which sets it apart from the classical methods for producing aminophosphinic acids. These methods involve the nucleophilic addition of phosphorus-containing compounds, such as hypophosphorous acid, trimethylsilyl phosphonites, or alkyl phosphinates, to imines, followed by the removal of the protective groups (Baylis et al., 1984; Grobelny, 1987; Jiao et al., 1994; Yao and Yuan, 2013; Ordóñez et al., 2016). One-step synthesis of **Met-P**_**H**_ from 3-(methylthio)propanal oxime (Figure 1, method 1) is preferable if compared with the alkylation of homocysteine *H*-phosphinic analog (**Hcy-P**_**H**_) with MeI (Figure 1, method 2). The key step of the synthesis of a racemic *H*-phosphinic analog of SAH (*rac-* **SAH-P**_**H**_) was the alkylation of **Hcy-P**_**H**_ with 5′-deoxy-5′-chloroadenosine in aqueous DMSO, affording the target *rac-* **SAH-P**_**H**_ in gram scale as an epimer mixture at the alpha-carbon atom (Figure 1, Supplementary Scheme S2). Methylation of *rac-* **SAH-P**_**H**_ with CH_3_I in the HCOOH/dioxane mixture resulted in the *H*-phosphinic analog of SAM (*rac-* **SAM-P**_**H**_) in 62% yield as a mixture of four diastereomers with a ratio 1.3:1.3:1:1 (Figure 1; Supplementary Figures S52, S54). Racemic phosphonic analogs of methionine and SAH (*rac-* **Met-P**_**5**_ and *rac-* **SAH-P**_**5**_, respectively) were obtained by the oxidation of the corresponding *H*-phosphinic compounds with iodine with excellent yields (Figure 1). Racemic phosphonic analog of SAM (*rac-* **SAM-P**_**5**_) was synthesized similarly to that for *rac-* **SAM-P**_**H**_ **,** starting from *rac-* **SAH-P**_**5**_ with 50% yield (Figure 1, Supplementary Scheme S3).

### 2.2 SAM-P_H_ and SAM-P_5_ are more chemically stable than SAM

**FIGURE 2:**
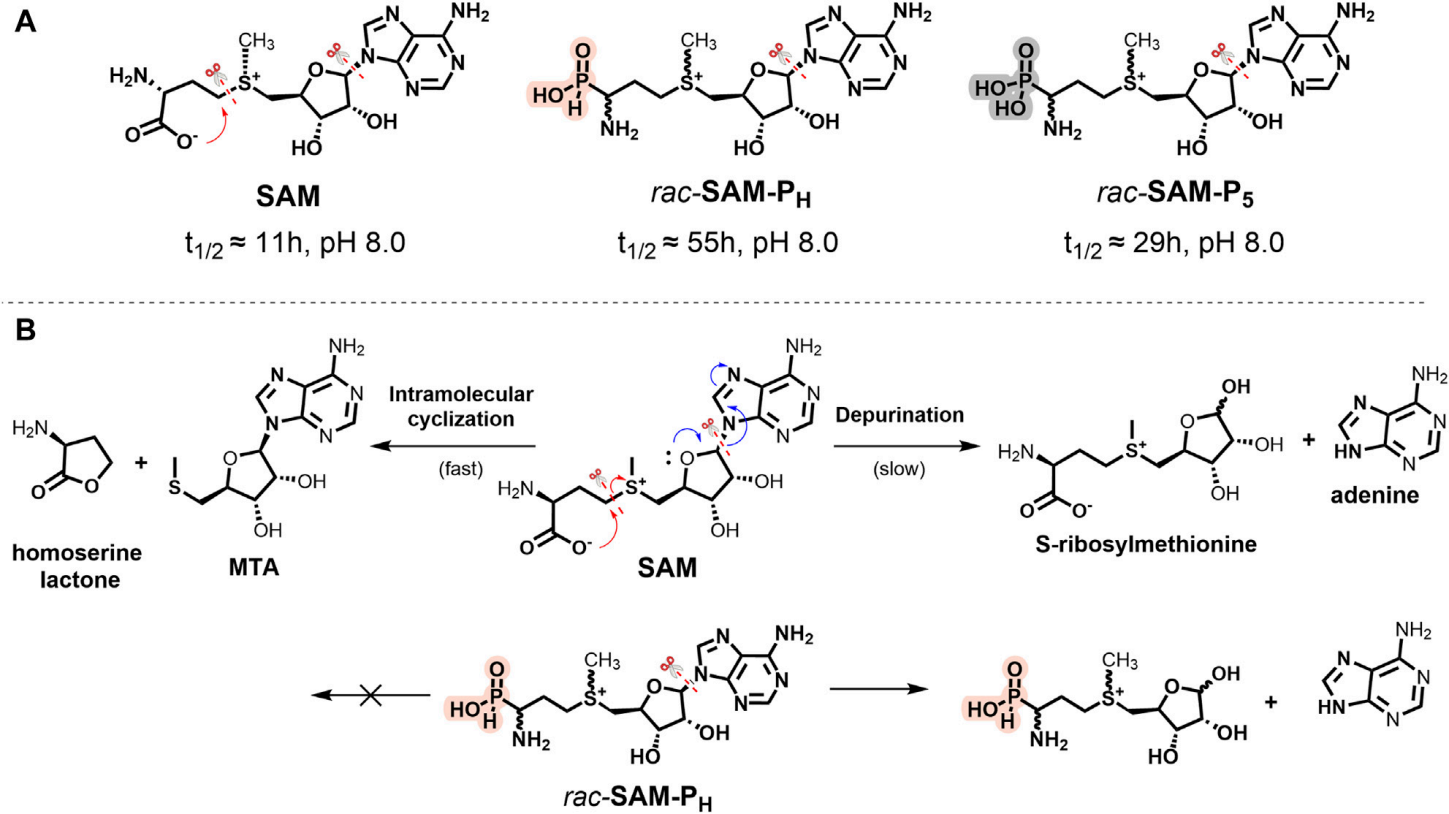
**(A)** Stability of SAM, *rac-* **SAM-P**_**H**_, and *rac-* **SAM-P**_**5**_. Half-life time (t_1/2_) of compounds was measured in 100 mM Tris-d_11_, pH 8.0, and 37°C. The symbol “scissors” marks the main degradation pathways of the molecule. **(B)** Scheme of SAM (top) and *rac-* **SAM-P**_**H**_ (bottom) degradation at pH 8.0.

SAM is a relatively unstable compound, and at pH > 1.5, in aqueous solutions, it undergoes intramolecular cyclization to form homoserine lactone and methylthioadenosine (MTA, Figure 2B). At neutral and alkaline pH, hydrolysis of SAM to adenine and *S*-ribosylmethionine is also observed (Parks and Schlenk, 1958a; Parks and Schlenk, 1958b; Borchardt, 1979; Wu et al., 1983; Hoffman, 1986; Iwig and Booker, 2004).

NMR monitoring of SAM, *rac-* **SAM-P**_**H**_, and *rac-* **SAM-P**_**5**_ in 100 mM Tris-HCl at pH 8.0 and 37°C showed that the overall stabilities of *rac-* **SAM-P**_**5**_ and *rac-* **SAM-P**_**H**_ were approximately two and five times higher than that of SAM, respectively (Figure 2A; Supplementary Figure S1). Under these conditions, SAM simultaneously degrades along two pathways (Figure 2B): intramolecular cyclization (fast) and depurination (slow). It is known that some γ-substituted alkyl *H*-phosphinic and phosphonic acids can undergo intramolecular cyclization and yield phostones under drastic conditions (Xu, 2023). The lactone-like decomposition pathway is impossible for **SAM-P**_**H**_ and **SAM-P**_**5**_ under physiological conditions. Only the slow depurination process occurs in the case of **SAM-P**_**H**_ and **SAM-P**_**5**_ (Figure 2B). The stability of **SAM-P**_**H**_ and **SAM-P**_**5**_ will be important while working with phosphorus-containing analogs of SAM having alkyl modifications at the sulfonium center, allowing labeling methyltransferase substrates with the reporter groups.

### 2.3 Methionine adenosyltransferase 2A catalyzes the synthesis of (*R,S*)-SAM-P_H_ from Met-P_H_ and ATP

**FIGURE 3:**
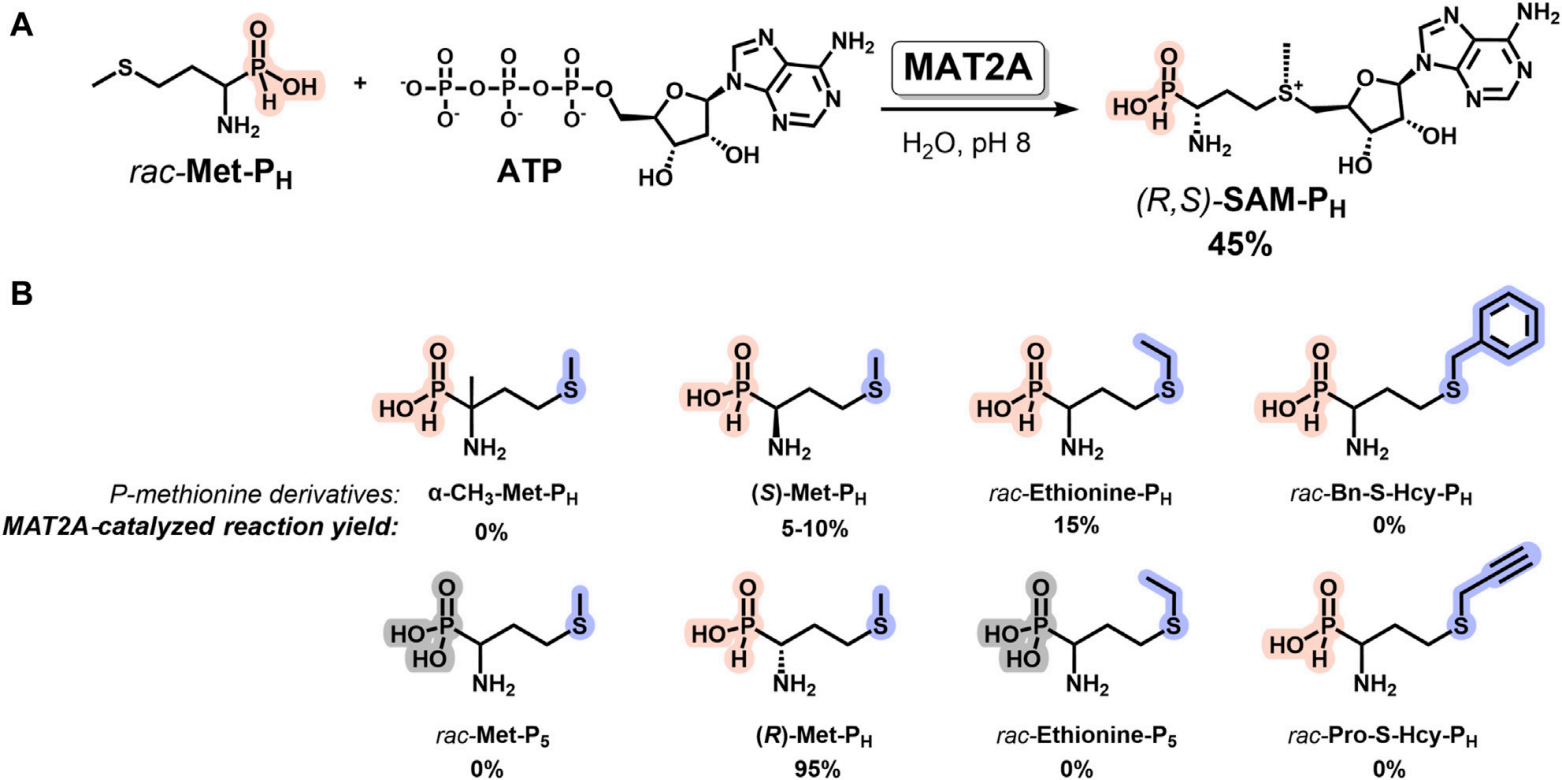
MAT2A-catalyzed synthesis of diastereomerically pure *H*-phosphinic analog of SAM (*R,S* **-SAM-P**_**H**_) and its derivatives. **(A)** Synthesis of (*R,S* **-SAM-P**_**H**_) from *rac-*Met-P_H_ and ATP. **(B)** *H*-Phosphinic and phosphonic analogs of methionine as substrates of MAT2A. Reaction conditions: *H*-Phosphinic or phosphonic analogs of methionine 5 mM, 1 mM ATP, 25 mM Tris-d_11_, pH 8.0, 50 mM KCl, 10 mM MgCl_2_, 37°C, and 50 μM MAT2A.

Methionine adenosyltransferase 2A (MAT2A, E.C. 2.5.1.6) catalyzes the synthesis of SAM from methionine and adenosine triphosphate (ATP) in mammals. MAT2A has strict stereospecificity and uses *L*-methionine as a substrate (Markham et al., 1987; Komoto et al., 2004; Ottink et al., 2010; Zhang and Klinman, 2015; Niland et al., 2021). This enzyme is widely used for the biocatalytic synthesis of SAM analogs from ATP and methionine derivatives (Singh et al., 2014; Cornelissen et al., 2020; Gade et al., 2021; Erguven et al., 2022; Peters et al., 2022; Rudenko et al., 2022; Gericke et al., 2023; Hoffmann et al., 2023; Mohr et al., 2023). The synthesis of functionally active isosteric SAM analogs containing a tetrazole group instead of a carboxyl group is an example (Huber et al., 2016). Here, we describe the substrate properties of the family of phosphorus-containing analogs of methionine (Figure 3) in the MAT2A reaction. The interaction of *rac-* **Met-P**_**H**_ with ATP in the presence of MAT2A under optimal pH 8 leads to the formation of a diastereomerically pure *H*-phosphinic analog of SAM (*R,S* **-SAM-P**_**H**_) in 45% yield, as calculated for *rac-* **Met-P**_**H**_, since the *(R)-* **Met-P**_**H**_ (*L*-isomer) of *rac-* **Met-P**_**H**_ mainly reacts (Figure 3A). The phosphonic analog of methionine (*rac-* **Met-P**_**5**_) exhibited no substrate properties under the same conditions. The substrate properties of *(S)-* **Met-P**_**H**_ (*D*-isomer) were considerably worse (Figure 3B). The *H-*phosphinic analog of ethionine (*rac-* **Ethionine-P**_**H**_) was a poor substrate compared to *rac*-**Met-P**_**H**_, while the *H*-phosphinic analog of *S*-propargyl homocysteine (*rac-* **Pro-S-Hcy-P**_**H**_) was not a substrate. Surprisingly, the *H*-phosphinic analog of α-methyl methionine (α-**CH**_**3**_ **-Met-P**_**H**_) also was not a substrate.

Thus, a diastereomerically pure *H*-phosphinic analog of SAM (*R,S* **-SAM-P**_**H**_, Figure 3A) has been enzymatically synthesized for the first time. However, for the synthesis of *H*-phosphinic analogs of SAM with substituents different from the methyl group, another MAT synthetase with broader substrate specificity, for example, PC-MjMAT (Peters et al., 2022) or HMT (see below), may be used.

### 2.4 Halide methyltransferase catalyzes synthesis of (*R,S*)-SAM-P_H_ from *rac-*SAH-P_H_

MAT2A is capable of catalyzing the synthesis of **SAM-P**_**H**_ analogs only with methyl and ethyl groups in the sulfonium center, while the bulkier *S*-substituted Met-P_H_ analogs did not exhibit substrate activity (Figure 3). An alternative enzymatic approach for the synthesis of SAM analogs is to use halide methyltransferase (HMT, E.C. 2.1.1.165), catalyzing the formation of SAM from SAH and methyl iodide (Tang et al., 2021b). The engineered *Arabidopsis thaliana* HMT variant V140T (*At*HMT^V140T^) has broad substrate specificity and can utilize bulky alkyl iodides as alkyl group donors (Tang et al., 2021a). HMTs were recently used for the synthesis of several *S*-substituted SAM analogs (Tang et al., 2021a; Tang et al., 2021b; Peng et al., 2021; Schülke et al., 2022; Schülke et al., 2024; Wen et al., 2022; Yang et al., 2023; Gao et al., 2024). However, to the best of our knowledge, there is currently no literature data on the substrate properties of phosphorus analogs of SAH, including **SAH-P**_**H**_ and any other SAH analogs with bioisosterically substituted carboxyl group, in the reaction catalyzed by HMT.

**FIGURE 4:**
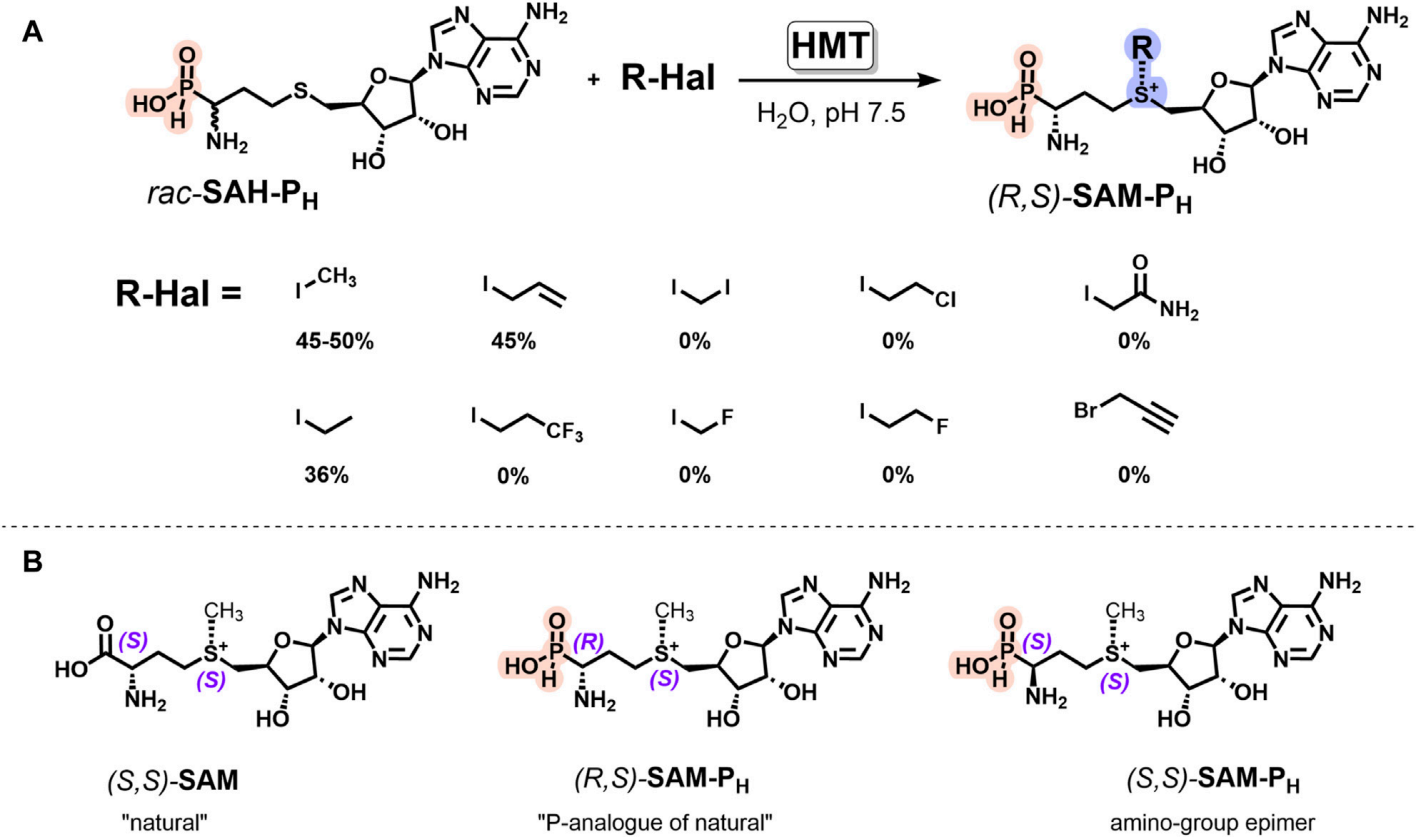
HMT-catalyzed synthesis of the diastereomerically pure *H*-phosphinic analog of SAM and its derivatives. **(A)** *At*HMT^V140T^-catalyzed synthesis of *(R,S)-* **SAM-P**_**H**_ derivatives from *rac*-**SAH-P**_**H**_ and alkyl iodides. Reaction conditions: 2 mM *rac*-**SAH-P**_**H**_, 20 mM alkyl iodides, 50 mM chloride-free potassium phosphate buffer, pH 7.5, 37°C, and 40 μM *At*HMT^V140T^. **(B)** Stereochemical configurations of SAM and its phosphorus analogs. Natural *(S,S)*-SAM, its analog *(R,S)*-**SAM-P**_**H**_, and amino-group epimer *(S,S)*-**SAM-P**_**H**_.

Here, we demonstrated that (*R,S*)-**SAM-P**_**H**_ can be synthesized from *rac-* **SAH-P**_**H**_ and methyl iodide using *At*HMT^V140T^ (Figure 4A). This reaction exhibits Michaelis–Menten kinetics (Supplementary Figure S7). The K_m_ value for the methyl iodide was 5.3 ± 3 mM, and the K_cat_ value was 18 ± 2 min^−1^. Interestingly, *rac-* **SAH-P**_**5**_ did not exhibit substrate activity in the reaction catalyzed by *At*HMT^V140T^, i.e., the enzyme is capable of distinguishing *H*-phosphinic and phosphonic groups.

Enzymatic alkylation of natural SAH yields (*S,S*)-SAM with the natural (*S*)-configuration of sulfonium center (Figure 4B) that favors synthetic applications of HMT. When *rac*-**SAH-P**_**H**_ was used as a starting compound, only one **SAM-P**_**H**_ diastereomer having an *L-*configuration of the amino-*H*-phosphinic group and natural (*S*)-configuration of the sulfonium center, i.e., (*R,S*)-**SAM-P**_**H**_, was preferentially formed (Supplementary Figures S8, S11, S12). Increasing the enzyme concentration allowed the conversion of the remaining (*S*)-**SAH-P**_**H**_ (analog of (*D*)-SAH) into (*S,S*)-**SAM-P**_**H**_, having an unnatural configuration. There is no literature available on the use of (*D*)-SAH in HMT reaction, making it challenging to explain the substrate properties of (*S*)-**SAH-P**_**H**_ in HMT reaction. However, this observation is interesting and may prove useful.

HMT-dependent *rac-* **SAH-P**_**H**_ alkylation was successfully scaled up, and 22 mg of (*R,S*)-**SAM-P**_**H**_ was obtained from 39 mg of *rac*-**SAH-P**_**H**_ (Supplementary Material).

The introduction of different iodides into the reaction with *rac*-**SAH-P**_**H**_ revealed that *At*HMT^V140T^ catalyzes alkylation with only ethyl and allyl iodides, with the yields being similar to those observed for SAH and the corresponding iodides (Figure 4A). Possibly, using HMT from other organisms or genetically modified enzymes will allow expanding the spectrum of synthesized **SAM-P**_**H**_ analogs and will be helpful in the synthesis of **SAM-P**_**H**_ derivatives having the substituents of biological importance or reporter groups at the sulfonium center.

### 2.5 SAM-P_H_ is a substrate of сatechol-*O*-methyltransferase

**FIGURE 5:**
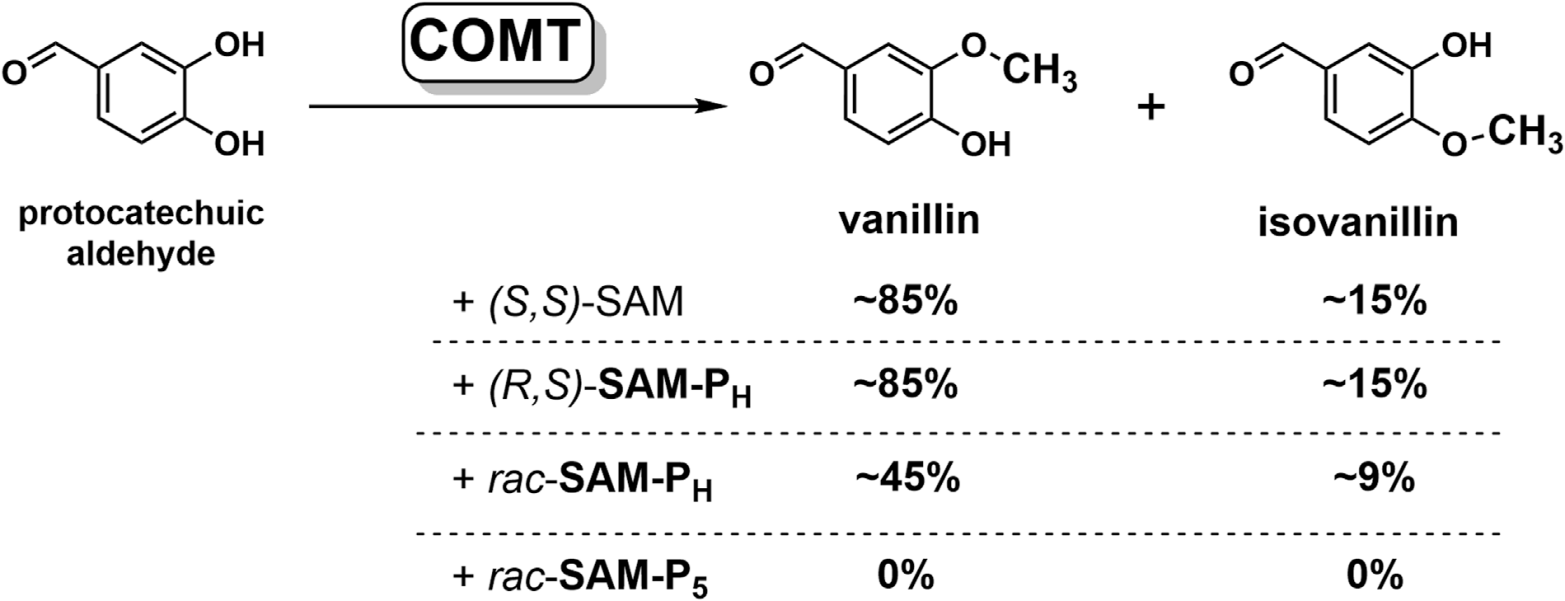
SAM and its phosphorus analogs as methyl group donors in the methylation of protocatechuic aldehyde catalyzed by COMT. Conversion of substrate to products, determined by NMR, is indicated. Reaction conditions: 12 mM SAM or its analogs, 3 mM protocatechuic aldehyde, 18 mM MgCl_2_, 50 mM potassium phosphate, pH 7.5, 37°C, and 20 μM COMT.

SAM and its analogs are mainly applied in biocatalytic reactions as cofactors of MTases. To test the ability of MTases to recognize SAM phosphorus analogs, we studied substrate properties of these analogs in the reaction of protocatechuic aldehyde methylation by сatechol-*O*-methyltransferase (COMT, EC 2.1.1.6), which is a typical model of MTase class I (Figure 5).

**FIGURE 6:**
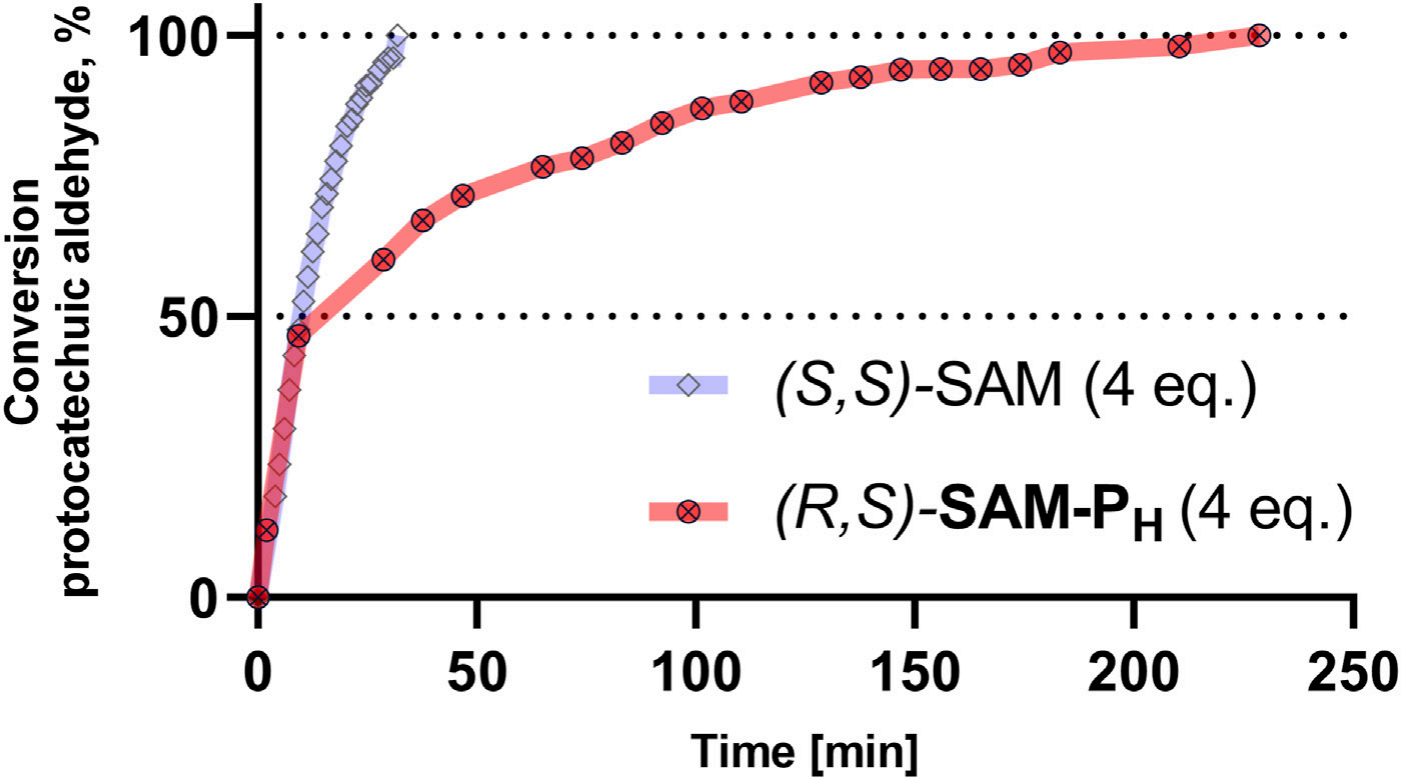
COMT-catalyzed methylation of protocatechuic aldehyde using (*S,S*)-SAM (blue) and (*R,S*)-**SAM-P**_**H**_ (red). Conversion of protocatechuic aldehyde to vanillin–isovanillin was monitored by NMR. Reaction conditions are the same as in Figure 5.

In the presence of four equivalents of *(R,S)-* **SAM-P**_**H**_, COMT fully converts protocatechuic aldehyde into vanillin and isovanillin (Figure 5, Supplementary Figures S13, S14). Notably, the ratio of vanillin–isovanillin is the same in the case of *(R,S)-* **SAM-P**_**H**_ and SAM, which indicates the correct recognition of *(R,S)-* **SAM-P**_**H**_ as a methyl group donor. However, when *(R,S)-* **SAM-P**_**H**_ was used as a cofactor, a longer time was required to achieve a complete methylation of protocatechuic aldehyde than when using SAM (Figure 6 and Supplementary Figure S14). Finally, *rac-* **SAM-P**_**5**_ did not exhibit any cofactor activity in the COMT-catalyzed reaction.

### 2.6 Methyltransferase WBSCR27 binds to SAM phosphorus analogs in the native co-factor binding site

To study the structural aspects of the binding of organophosphorus SAM mimetics to methyltransferases at the atomic level, we used WBSCR27, a Williams–Beuren syndrome-related methyltransferase from *Mus musculus*. We previously showed that WBSCR27 tightly binds SAM and SAH (Mariasina et al., 2020). Complete NMR assignments were obtained for the protein in the apo-form and for the WBSCR27 complexes with SAM and SAH (Mariasina et al., 2018). The 3D solution structure was determined for the apo-form and the complex of WBSCR27 with SAH (Mariasina et al., 2022). The available data allow studying the structural aspects of phosphorus-containing SAM mimetics binding to the active site of this methyltransferase using NMR spectroscopy techniques.

**FIGURE 7:**
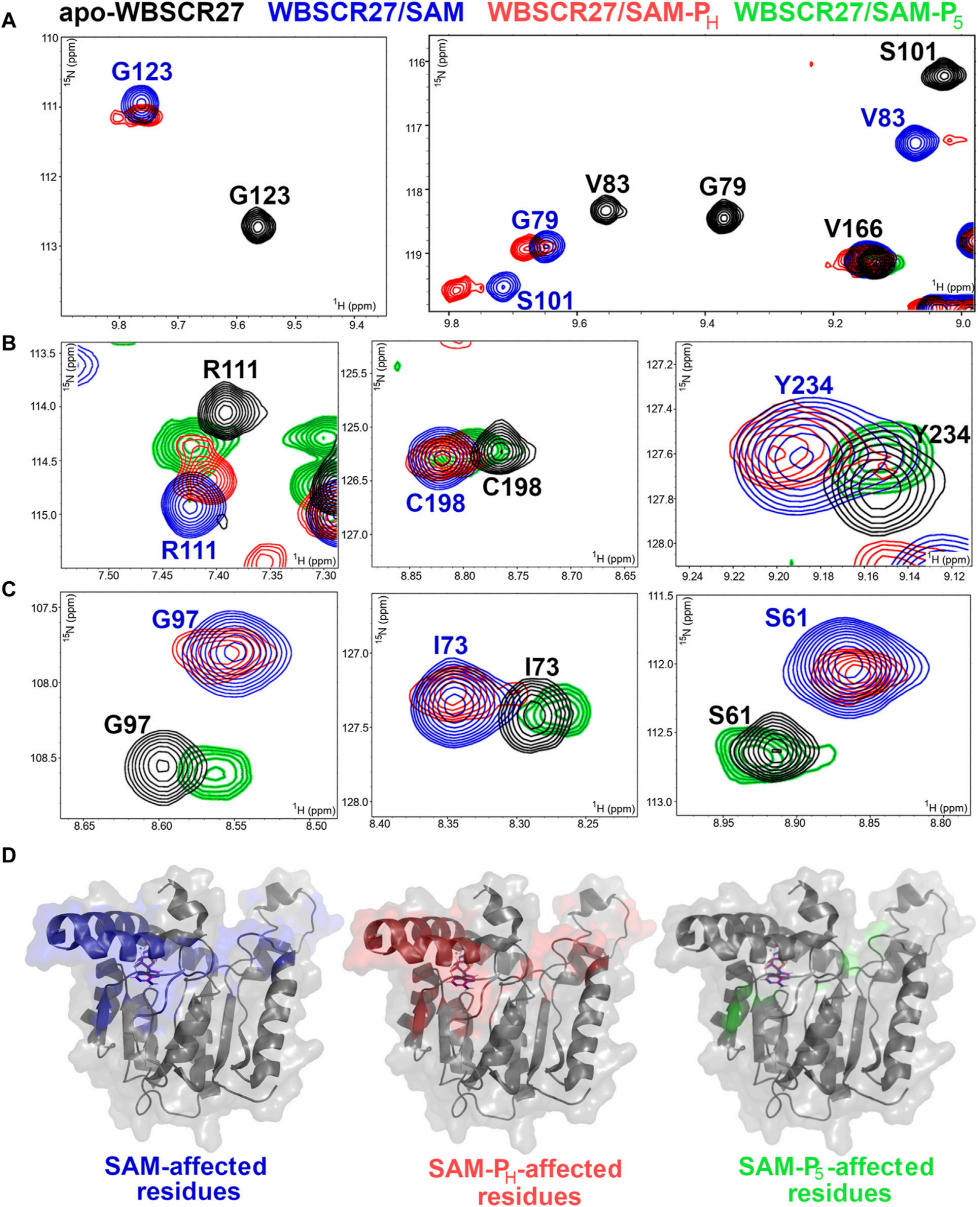
SAM, **SAM-P**_**H**_, and **SAM-P**_**5**_ interactions with methyltransferase WBSCR27. **(A–C)** Fragments of ^1^H,^15^N HSQC spectra of ^15^N-labeled WBSCR27 in the apo-form (black) in the presence of SAM (blue), *rac-* **SAM-P**_**H**_, (red), and *rac-* **SAM-P**_**5**_ (green). Labels indicate the amino acid residues: black labels for apo-WBSCR27 and blue labels for WBSCR27 in the presence of SAM. **(A)** Signals of residues directly involved in ligand binding disappear from the spectrum of WBSCR27/**SAM-P**_**5**_ due to the fast dynamic exchange between the bound/unbound state. **(B)** Signals of residues in which WBSCR27/**SAM-P**_**5**_ chemical shifts are between its positions in apo-WBSCR27 and WBSCR27/SAM complex. **(C)** Signals of residues in which WBSCR27/**SAM-P**_**5**_ chemical shifts are almost identical to those in apo-WBSCR27. **(D)** The structure of the methyltransferase WBSCR27 in its SAH-bound state (PDB id 7QCB) is shown. Residues that have a significant difference in chemical shift between the apo-state and the ligand-bound state are highlighted in different colors: blue for SAM binding, red for **SAM-P**_**H**_ binding, and green for **SAM-P**_**5**_ binding. The SAH molecule is represented by magenta sticks in all structures.

WBSCR27 strongly binds to **SAM-P**_**H**_. The positions of the WBSCR27 signals in the NMR spectra are almost identical for the WBSCR27/**SAM-P**_**H**_ and WBSCR27/**SAM** complexes (Figures 7A–C, blue and red residues in Supplementary Figure S15). These data suggest that the spatial arrangement of **SAM-P**_**H**_ at the WBSCR27 binding site is very similar to that of SAM.

On the contrary, *rac-* **SAM-P**_**5**_ demonstrated a weaker interaction with WBSCR27. Signals from amino acid residues located in the ligand-binding site disappeared from the spectrum due to a conformational exchange between the bound and unbound states of the protein (Figures 7A, Supplementary Figure S16). The signals of all other amino acid residues associated with the cofactor binding site are located either between their positions in apo-WBSCR27 and the WBSCR27/SAM complex (Figures 7B, Supplementary Figure S16) or have chemical shifts identical to apo-WBSCR27 (Figures 7C, Supplementary Figure S16). Therefore, the lack of functional activity of *rac-* **SAM-P**_**5**_ in the COMT and *rac-* **SAH-P**_**5**_ in HMT reactions may also be explained by its weaker binding to MTase when compared with that of SAH and **SAM-P**_**H**_.

The residues with high chemical shift perturbations between the apo-form, SAM, **SAM-P**_**H**_, and **SAM-P**_**5**_-bound states were mapped onto the 3D structure of WBSCR27 (Figure 7D). The distribution of the affected residues is almost identical between WBSCR27/SAM and WBSCR27/**SAM-P**_**H**_ complexes. In contrast, only a few residues have high chemical shift perturbations between the apo-form and the **SAM-P**_**5**_-bound state (Figure 7D, right).

### 2.7 Methylthioadenosine nucleosidase recognizes phosphorus analogs of SAH but not those of SAM

**FIGURE 8:**
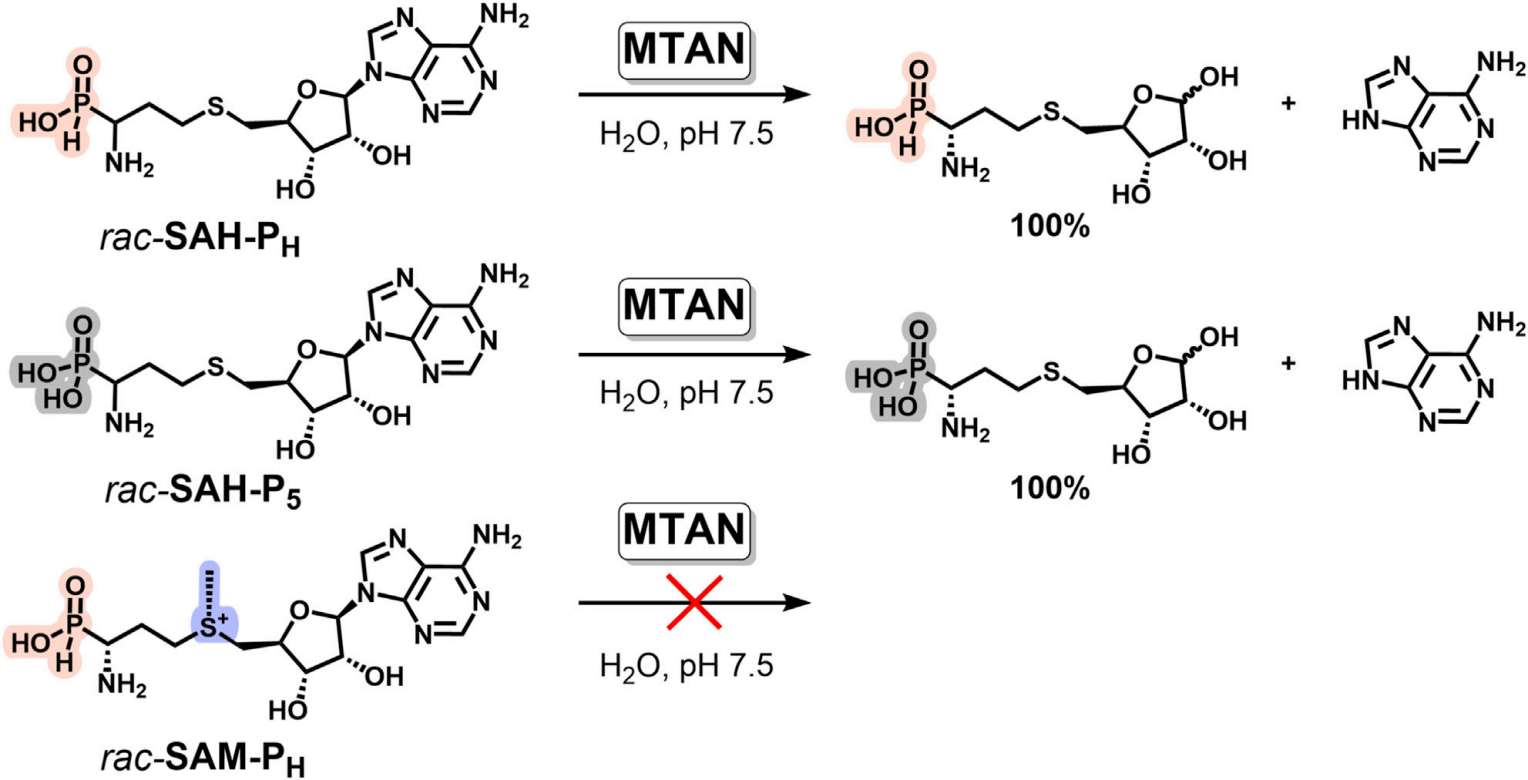
Phosphorus analogs of SAH, but not of SAM, are the substrates of methylthioadenosine/*S-*adenosylhomocysteine nucleosidase (MTAN). Reaction conditions: 2 mM phosphorus analog of SAH or SAM, 50 mM potassium phosphate, pH 7.5, 37°C, and 0.1 μM MTAN.

SAH is the co-product of SAM-dependent methyltransferase reactions and is an inhibitor of most methyltransferases (Ueland, 1982). To increase the yield of the methylated product, methylthioadenosine/*S*-adenosylhomocysteine nucleosidase (MTAN, EC 3.2.2.16), an enzyme cleaving SAH to adenine and *S*-(5-ribosyl)-homocysteine, is often added to the reaction mixture (Siegrist et al., 2017). We investigated the ability of MTAN to degrade phosphorus-containing mimetics of SAH. Both **SAH-P**_**H**_ and **SAH-P**_**5**_ are the substrates of MTAN and rapidly undergo glycoside bond cleavage, with the formation of adenine and the corresponding ribose derivative (Figure 8). The reaction was monitored by NMR and HPLC (Supplementary Figures S18–S20).

To be effectively used as a methylation accelerator, MTAN should not catalyze the degradation of SAM analogs. We have shown that neither **SAM-P**_**H**_ nor **SAM-P**_**5**_, nor native SAM, undergoes glycoside bond cleavage under similar conditions. Therefore, addition of MTAN can be used in methyltransferase reactions with different *H*-phosphinic SAM analogs to remove **SAH-P**_**H**_ from the reaction mixture and increase the yield of the methylated product.

## 3 Discussion

One of the modern trends for studying methyltransferase reactions is the enzymatic derivatization of substrate biomolecules using SAM analogs with a reporter group instead of a methyl group of SAM. However, many such derivatives of natural SAM are chemically unstable that largely limits their practical application. Several functionally active mimetics of SAM with sufficient chemical stability are known, such as tetrazole derivatives, selenium-containing derivatives (SeAM) (Sohtome et al., 2021; Cornelissen et al., 2023), decarboxylated SAM, and others (Rudenko et al., 2022). However, the existing range of such SAM analogs needs to be expanded as biomethylation reactions are highly diverse and numerous, making it unlikely that the known analogs can be as effective as SAM in all transformations. In this study, *(R,S)-* **SAM-P**_**H**_ is described as an original functionally active mimic of SAM, which may serve as a promising scaffold for the synthesis of new tools for studying methyltransferases and their substrates.

**SAM-P**_**H**_, bearing an *H-*phosphinic group instead of a carboxyl group, is significantly more chemically stable compared to SAM and is comparable in stability to the tetrazole derivative (Figure 2). This property of **SAM-P**_**H**_ meets the current criteria for chemically stable and functionally active analogs of SAM.

However, in addition to chemical stability, novel SAM analogs must exhibit good substrate properties in reactions catalyzed by methyltransferases. In the reaction catalyzed by COMT, **SAM-P**_**H**_ retains the functional activity of SAM, maintaining the vanillin/isovanillin ratio, although the reaction proceeds somewhat slower (Figure 5). The binding of **SAM-P**_**H**_ to methyltransferases at the molecular level was investigated using WBSCR27 as an example. ^1^H,^15^N HSQC NMR analysis of WBSCR27 complexes showed that the perturbations of chemical shifts of enzyme amino acid residues were very similar for *rac-* **SAM-P**_**H**_ and SAM (Figure 7), indicating the identity of the binding site for these molecules. **SAM-P**_**H**_ also exhibited good substrate properties in the case of DNA methyltransferase Dnmt3A, which carries out *de novo* methylation of cytosine residues at the C5 position in CpG sequences of DNA (Filonov et al., 2023). Thus, the requirement to exhibit substrate properties in different methyltransferase reactions is fulfilled for **SAM-P**_**H**_.

In this study, we describe the chemical synthesis of *rac-* **SAM-P**_**H**_ from readily available starting materials, enabling the production of gram-scale quantities of *rac-* **SAM-P**_**H**_ (Figure 1 and Supplementary Scheme S2). Additionally, we propose, for the first time, an enzymatic method for obtaining diastereomerically pure **SAM-P**_**H**_, a bioisosteric analog of natural SAM. We have demonstrated that *(R,S)-* **SAM-P**_**H**_ can be enzymatically synthesized either from (*R*)-**Met-P**_**H**_/*rac-* **Met-P**_**H**_ using MAT2A (Figure 3) or from *rac-* **SAH-P**_**H**_ using HMT (Figure 4A).

The preference of *L*-methionine as a substrate in MAT2A reaction is well-known (Markham et al., 1987; Zhang and Klinman, 2015). *(R)-* **Met-P**_**H**_ (*L*-isomer) was a good MAT2A substrate (Figure 3B), which is in line with the stereospecificity of the enzyme. (*S*)-**Met-P**_**H**_ (*D*-isomer) exhibited weak substrate properties in the MAT2A reaction. It was not surprising because it is known that MAT from *E.coli* can utilize *D*-methionine as a substrate, but in this case, the K_m_ value is 175 times and V_max_ is 11 times worse when compared with that for *L*-isomer (Lu and Markham, 2002). However, in the case of MAT from archaeon *Methanococcus jannaschii*, the differences between *L*- and *D*-isomers of methionine are not so dramatic: K_m_ is only 7.7 times and V_max_ is 14.5 times worse (Lu and Markham, 2002).

During the methylation reaction, SAM is converted to SAH, which acts as a competitive inhibitor of MTase (Ueland, 1982; Mariasina et al., 2020). Several enzymatic methods have been developed and used to regenerate SAM from SAH directly in the substrate mixture (Popadić et al., 2021; Gericke et al., 2023). For example, methyl iodide in the presence of halide methyltransferase (HMT) has been successfully used for this purpose (Liao and Seebeck, 2019). Therefore, we studied substrate properties of *rac-* **SAH-P**_**H**_ in HMT-reaction and demonstrated that *(R,S)-* **SAM-P**_**H**_ can be synthesized using this enzyme from *rac-* **SAH-P**_**H**_ (Figure 4A).

It was found that in HMT-catalyzed reactions, (*R*)-**SAH-P**_**H**_ (*L*-isomer) is consumed first, followed by the reaction of the other diastereomer, (*S*)-**SAH-P**_**H**_ (*D*-isomer). Accordingly, the NMR spectra showed the initial formation of (*R,S*)-**SAM-P**_**H**_ from (*R*)-**SAH-P**_**H**_, followed by the formation of (*S,S*)-**SAM-P**_**H**_ from (*S*)-**SAH-P**_**H**_ (Supplementary Figures S8–S10, S56). To the best of our knowledge, the stereospecificity of the HMT reaction has not been studied yet. Thus, we successfully synthesized enzymatically *via* two independent pathways that makes this analog of SAM an easily accessible substance.

The obtained data suggest that (*R,S*)-**SAM-P**_**H**_ can be considered a new functionally active mimetic of SAM and a promising scaffold for generating a family of phosphorus-containing SAM analogs suitable for introducing reporter groups into methyltransferase substrates. Additionally, the substrate properties of **SAH-P**_**H**_ in the HMT reaction will be beneficial in substrate-conserving enzymatic cyclic reactions.

In the reactions catalyzed by MAT2A, HMT, COMT, methyltransferase WBSCR27, and MTAN in addition to *H*-phosphinic analogs of Met, SAH, and SAM, here, we studied the corresponding phosphonic derivatives having the (HO)_2_(O)P-group instead of the carboxyl group. Methyltransferase COMT uses **SAM-P**_**H**_, but not **SAM-P**_**5**_, as a methyl group donor (Figure 5). The same is true for phosphorus-containing methionine analogs and MAT2A, which catalyzes adenosylation of **Met-P**_**H**_ but not of **Met-P**_**5**_ (Figure 3). HMT can also discriminate *H*-phosphinic and phosphonic analogs, catalyzing methylation of **SAH-P**_**H**_ but not of **SAH-P**_**5**_ (Figure 4A). Chemical shift perturbation of amino acid residues of methyltransferase WBSCR27 in the ^1^H,^15^N HSQC NMR experiment clearly showed that the binding of *rac-* **SAM-P**_**H**_ in the cofactor binding site of WBSCR27 is very similar to that of SAM, whereas *rac-* **SAM-P**_**5**_ binds to the weaker protein, leaving the binding site more similar to that of the apo-form of the enzyme (Figure 7).

Such differences in the activity of *H*-phosphinic and phosphonic analogs arise due to variations in the geometry of the monobasic *H*-phosphinic and dibasic phosphonic groups. The *H*-phosphinic group adopts an oblate tetrahedral conformation due to the smaller volume of the hydrogen atom compared to the hydroxyl group in the phosphonic acid. The free rotation of the *H*-phosphinic group can ensure the orientation of this hydrogen atom in the direction of minimal steric effect in the binding site. Meanwhile, the distances between the charged oxygen atoms in *H*-phosphinic and carboxyl groups are similar, which ensures an equally effective interaction with amino acid residues in the enzyme active center. Thus, amino-*H*-phosphinic acids, in contrast to aminophosphonic acids with a doubly charged (HO)_2_P(O) group, turn out to be complementary enough to the active site of enzymes. This relationship between the structure and functional activity is typical not only for the enzymes considered in the present work but also for PLP-dependent methionine-γ-lyase (Faleev et al., 2009), tyrosine-γ-lyase (Faleev et al., 2000), and glutamate decarboxylase using the distal *H*-phosphinic analog of glutamate as a substrate (De Biase et al., 2020). However, this difference between the substrate properties of *H*-phosphinic and phosphonic analogs of amino acids is not a strict rule. Thus, *rac-* **SAM-P**_**H**_ and *rac-* **SAM-P**_**5**_ exhibited equal functional activity in the case of DNA methyltransferase Dnmt3A, carrying out *de novo* methylation of cytosine residues at the C5 position in CpG sequences of DNA (Filonov et al., 2023), and, as we have shown here, MTAN can split both *rac-* **SAH-P**_**H**_ and *rac-* **SAH-P**_**5**_ (Figure 8).

The possibility of **SAM-P**_**H**_ biosynthesis, which we have shown here for the first time using the isolated MAT2A enzyme (Figure 3), may be one of the reasons for the biological activity of **Met-P**_**H**_. This methionine analog has superior fungicidal activity in field trials (equal to the Japanese fungicide, Fujione^®^) against rice blast disease caused by *Pyricularia oryzae* (Zhukov et al., 2004). The biochemical properties and mechanism of fungicidal activity of **Met-P**_**H**_ are poorly studied, and the biosynthesis of **SAM-P**_**H**_ from **Met-P**_**H**_ is crucial and may be directly related to the biological activity of **Met-P**_**H**_. First, **SAM-P**_**H**_ *a priori* cannot serve as an optimal methyl group donor in reactions catalyzed by all types of methyltransferases. Even if **SAM-P**_**H**_ has functional activity as a cofactor, it is likely to be less effective than SAM. The latter was recently confirmed by studying the interaction of *rac-* **SAM-P**_**H**_ with DNA methyltransferase Dnmt3a. *rac-* **SAM-P**_**H**_ turned out to be a two times worse substrate than SAM when recalculated to active diastereomer (Filonov et al., 2023). In the present study, COMT also demonstrated a slower reaction rate with *(R,S)-* **SAM-P**_**H**_ compared to SAM (Supplementary Figure S14). Second, *de novo* synthesized *(R,S)-* **SAM-P**_**H**_ may be recognized as SAM in cells, similar to the recognition of *(R)-* **Met-P**_**H**_ as methionine in *Citrobacter intermedius*, resulting in the induction of methionine-γ-lyase biosynthesis (Alferov et al., 2002). Recognition of *(R,S)-* **SAM-P**_**H**_ as SAM may alter the intracellular native SAM/SAH ratio, which is crucial for the efficient regulation of numerous methylation reactions, including epigenetically important pathways. The above considerations may provide the background for the molecular mechanism of the biological activity of *(R)-* **Met-P**_**H**_, which can be intracellularly converted into *(R,S)-* **SAM-P**_**H**_.

## 4 Conclusion

A new SAM analog, *rac*-**SAM-P**_**H**_, has been chemically synthesized, replacing SAM’s carboxylic group with the *H-*phosphinic group (-P(O)(H)OH). Diastereomerically pure *(R,S)-* **SAM-P**_**H**_, which retains the same stereoconfiguration as native SAM, was enzymatically synthesized for the first time using methionine adenosyltransferase 2A or halide methyltransferases from *(R)-* **Met-P**_**H**_ and *rac-* **SAH-P**_**H**_, respectively. **SAM-P**_**H**_ turned out to be significantly more chemically stable than SAM. The functional activity of **SAM-P**_**H**_ in the сatechol-*O*-methyltransferase reaction and interaction with the methyltransferase WBSCR27 is similar to that of native SAM. Replacing the methyl group in **SAM-P**_**H**_ with a reporter group, which can be used to label methyltransferase substrates, could lead to a valuable tool for studying metabolic transformations. The possibility of intracellular biosynthesis of *(R,S)-* **SAM-P**_**H**_ from *(R)-* **Met-P**_**H**_ may open new avenues in designing specific inhibitors of microbial and tumor cell growth.

## 5 Experimental section

### 5.1 Chemistry

#### 5.1.1 Materials and methods

Oximes were synthesized as described in Metzger (1971). Anhydrous H_3_PO_2_ was prepared by concentrating the commercially available 50% aqueous hypophosphorous acid (Aldrich) at 1 mbar and a temperature not exceeding 40°C and subsequent co-evaporation *in vacuo* with abs. *i*-PrOH. *R*- and *S*-isomers of Met-P_H_ were resolved by recrystallization of the (+)- and (−)-α-methylbenzylamine salts of *N*-benzyloxycarbonyl derivative of Met-P_H_, following a previously published protocol (Baylis et al., 1984). Propargyl chloride (Aldrich) was distilled before use. All other reagents, salts, and solvents were of the highest purity and used as supplied by Aldrich and Acros.

Ion-exchange chromatography was carried out on a Dowex 50W-X8, H^+^-form, 100–200 mesh (BioRad) or on the same resin in Py-form; systems for elution are indicated in the text. Flash chromatography was performed on silica gel (particle sizes 40–63 μm, pore size 60 Å, 230-400 mesh, Merck). Systems for elution are indicated in the text.

TLC was carried out on Alufolien Kieselgel 60 F_254_ plates (Merck) in *i*-PrOH-25% NH_4_OH-H_2_O = 7:1:2 (A); *n*-BuOH-AcOH-H_2_O = 12:3:5 (B); and on Plastikfolien Cellulose F_254_ (Merck) in *n*-BuOH-AcOH-H_2_O = 12:3:5 (C). Phosphorus-containing analogs of amino acids, SAH, and SAM were detected on TLC plates by UV light or by using color reaction with ninhydrin.

The melting points were determined in open capillary tubes on a Mel-Temp^®^ Instrument (Barnstead International, Dubuque, Iowa, USA) and are given uncorrected. High-resolution ESI mass spectra were measured on a Waters Xevo G3 QTof (Waters, Framingham, USA) spectrometer.

#### 5.1.2 NMR analysis

^1^H NMR spectra were recorded on a Bruker AVANCE 600 spectrometer (600.13 MHz) at 25°С. Chemical shifts for ^1^H NMR were reported as δ values, and coupling constants were reported in Hertz (Hz). The following abbreviations were used for spin multiplicity: s = singlet, d = doublet, t = triplet, dd = double doublet, m = multiplet, and bs = broad singlet. Chemical shifts were referenced to sodium trimethylsilylpropanesulfonate (DSS) as the internal standard and reported in parts per million (ppm). All spectra were processed with MestReNova 14.2.2.

#### 5.1.3 General method of racemic 1-amino-*H*-phosphinic acid synthesis

To the almost boiling solution of two equivalents of anhydrous H_3_PO_2_ in abs. alcohol, one equivalent of the corresponding oxime was added dropwise with stirring under an Ar atmosphere (keeping the reaction slightly boiling). The reaction mixture was refluxed for an additional 4–16 h, cooled, and the crude product was precipitated from the reaction mixture after the pH was adjusted to 4–4.5 with triethylamine. The product purification was performed by ion-exchange chromatography on cation exchange resin and subsequent crystallization from water–alcohol mixture.

##### 5.1.3.1 1-Amino-3-(methylthio)propyl-*H*-phosphinic acid (*rac-*Met-P_H_)

Method A: a solution of 3-methylthiopropanal oxime (Metzger, 1971) (17.8 g, 0.15 mol) in abs. MeOH (15 mL) was added dropwise with stirring under an Ar atmosphere to a boiling solution of anhydrous H_3_PO_2_ (20 g, 0.3 mol) in the mixture of MeOH (35 mL) and abs. *i*-PrOH (150 mL) over 45 min. The reaction mixture was refluxed for 4 h and cooled to 20°C, followed by the addition of Et_3_N to pH ∼ 4.5. The resulting mixture was kept at 4°C for 16 h; the precipitated material was filtered off, washed with abs. *i*-PrOH, and dried *in vacuo* over P_2_O_5_ to yield crude *rac-*Met-P_H_ (11.4 g). The product was purified on a column with Dowex 50W-X8 resin (H^+^-form, V = 500 mL) eluting with 15% aq. *i*-PrOH. Fractions containing *rac-*Met-P_H_ were concentrated *in vacuo*, and the residue was recrystallized from aq. *i*-PrOH. Crystals were dried *in vacuo* over P_2_O_5_ to yield *rac-*Met-P_H_ (8.1 g, 32%), mp 229°С (lit.: 231°С (Baylis et al., 1984)), *R*_f_ 0.53 (A), and *R*_f_ 0.36 (B).

Method B: *rac-*Met-P_H_ was synthesized, as described below for *rac-*Pro-S-Hcy-P_H_, starting from *rac-*Hcy-P_H_ (0.62 g, 4 mmol) and freshly distilled methyl iodide (0.5 mL, 8 mmol). The reaction mixture was left in the dark for 18 h at 20°C. *rac-*Met-P_H_ was isolated on a Dowex 50W-X8 column (H^+^-form, V = 100 mL) eluting with 15% aq. *i*- PrOH. Fractions containing *rac-*Met-P_H_ were evaporated to dryness *in vacuo* and dried *in vacuo* over P_2_O_5_ to yield *rac-*Met-P_H_ (0.6 g, 88%). The obtained compound was identical to that synthesized according to method A.

ESI-MS (m/z): the expected mass for C_4_H_13_NO_2_PS^+^ = 170.0399 [M + H]^+^, found: 170.0396. The expected mass for C_4_H_11_NO_2_PS^−^ = 168.0254 [M-H]^-^, found: 168.0252 ^**1**^ **H NMR** (600 MHz, D_2_O) δ = 7.0 (d, ^*1*^ *J*_PH_ = 534.8, 1H), 3.4–3.3 (m, 1H), 2.8–2.6 (m, 2H), 2.3–2.1 (m, 1H), 2.1 (s, 3H), and 2.0–1.9 (m, 1H). ^**13**^ **C NMR** (151 MHz, D_2_O) δ = 52.1 (d, *J* = 91.5), 32.1 (d, *J* = 9.7), and 28.1, 16.5. ^**31**^ **P NMR** (243 MHz, D_2_O) δ = 20.3.

##### 5.1.3.2 1-Amino-3-(ethylthio)propyl-*H*-phosphinic acid (*rac-*ethionine-P_H_)

*rac-*Ethionine-P_H_ was obtained as described for *rac-*Met-P_H_ starting with 3-ethylthiopropanal oxime (Metzger, 1971) (13.3 g, 0.1 mol) and anhydrous H_3_PO_2_ (13.2 g, 0.2 mol) by refluxing in abs. *i*-PrOH (100 mL) for 4 h. The reaction mixture was set up as described above for *rac-*Met-P_H_. Subsequent purification of crude *rac-*Ethionine-P_H_ was performed on Dowex 50W-X8 resin (H^+^-form, V = 350 mL), and elution was carried out with 15% aq. *i*-PrOH. Recrystallization from aq. *i*- PrOH yielded *rac-*Ethionine-P_H_ (4.9 g, 27%), mp 238°С, *R*_f_ 0.64 (A), and *R*_f_ 0.44 (B). **ESI-MS (m/z):** expected mass for C_5_H_15_NO_2_PS^+^ = 184.0556 [M + H]^+^, found: 184.0552. Expected mass for C_5_H_13_NO_2_PS^−^ = 182.041 [M-H]^-^, found: 182.0406. ^**1**^ **H NMR** (600 MHz, D_2_O) δ = 7.0 (d, *J* = 534.5, 1H), 3.3–3.3 (m, 1H), 2.8–2.8 (m, 1H), 2.8–2.7 (m, 1H), 2.6 (q, *J* = 7.4, 2H), 2.2–2.1 (m, 1H), 2.0–1.9 (m, 1H), and 1.2 (t, *J* = 7.4, 3H). ^**13**^ **C NMR** (151 MHz, D_2_O) δ = 52.2 (d, *J* = 91.5), 29.5 (d, *J* = 9.5), 28.8, 27.5, and 16.5. ^**31**^ **P NMR** (243 MHz, D_2_O) δ = 20.3.

##### 5.1.3.3 1-Amino-3-(benzylthio)propyl-*H*-phosphinic acid (*rac-*Bn-S-Hcy-P_H_)

A solution of 3-benzylthiopropanal oxime (Metzger, 1971) (52.2 g, 0.25 mol) in abs. *i*-PrOH (50 mL) was added dropwise with stirring under argon to a boiling solution of anhydrous H_3_PO_2_ (33 g, 0.5 mol) in abs. *i*-PrOH (200 mL) over 1 h. The reaction mixture was refluxed for 4 h and cooled. The precipitated material was filtered off, washed with abs. *i*-PrOH, and dried *in vacuo* over P_2_O_5_ to yield crude *rac-*Bn-S-Hcy-P_H_ (13.5 g). The combined filtrates were adjusted to pH ∼ 4.5 with Et_3_N and left at 20°C for 16 h to give additional amount of crude *rac-*Bn-S-Hcy-P_H_ (3.06 g). The pure compound was obtained by recrystallization from H_2_O, followed by drying *in vacuo* over P_2_O_5_ to yield *rac-*Bn-S-Hcy-P_H_ (12.9 g, 21%), mp 221°С (*Lit.* mp 221°C (Alferov et al., 2003)), *R*_f_ 0.61 (A), and *R*_f_ 0.46 (B). **ESI-MS (m/z):** expected mass for C_10_H_19_NO_2_PS^+^ = 246.0712 [M + H]^+^, found: 246.0706. Expected mass for C_10_H_17_NO_2_PS^−^ = 244.0567 [M-H]^-^, found: 244.0564. ^**1**^ **H NMR** (600 MHz, D_2_O) δ = 7.4–7.4 (m, 4H), 7.4–7.3 (m, 1H), 6.7 (d, *J* = 505.3, 1H), 3.8 (s, 2H), 2.7–2.7 (m, 2H), 2.6–2.5 (m, 1H), 2.0–1.9 (m, 1H), and 1.7–1.6 (m, 1H). ^**13**^ **C NMR** (151 MHz, D_2_O) δ = 141.3, 131.7, 131.5, 130.0, 52.5 (d, *J* = 98.8), 37.6, 31.4, and 30.2 (d, *J* = 14.1). ^**31**^ **P NMR** (243 MHz, D_2_O) δ = 32.6.

##### 5.1.3.4 1-Amino-1-methyl-3-(methylthio)propyl-*H*-phosphinic acid (α-CH_3_-Met-P_H_)

Methyl (2-methylthioethyl) ketone oxime (13.3 g, 0.1 mol) was added dropwise with stirring under Ar atmosphere to a boiling solution of anhydrous H_3_PO_2_ (13.2 g, 0.2 mol) in abs. *i*-PrOH (50 mL) over 30 min. The reaction mixture was refluxed for 16 h, cooled, and concentrated *in vacuo*. The residue was dissolved in 15% aq. *i*-PrOH and purified on Dowex 50W-X8 resin (H^+^-form, V = 300 mL) eluting with 15% aq. *i*-PrOH. Recrystallization from aq. *i*-PrOH yielded pure α-CH_3_-Met-P_H_ (7.2 g, 39%), mp 213°С, *R*_f_ 0.66 (A). **ESI-MS (m/z):** expected mass for C_5_H_15_NO_2_PS^+^ = 184.0556 [M + H]^+^, found: 184.0552. Expected mass for C_5_H_13_NO_2_PS^−^ = 182.041 [M-H]^-^, found: 182.0407. ^**1**^ **H NMR** (600 MHz, D_2_O) δ = 6.9 (d, *J* = 529.0, 1H), 2.8–2.6 (m, 2H), 2.1 (s, 3H), 2.1 (ddd, *J* = 11.7, 9.3, 7.4, 2H), and 1.4 (d, *J* = 14.4, 3H). ^**13**^ **C NMR** (151 MHz, D_2_O) δ = 57.4 (d, *J* = 94.9), 35.4, 29.4 (d, *J* = 7.1), 19.6 (d, *J* = 2.5), and 16.8. ^**31**^ **P NMR** (243 MHz, D_2_O) δ = 25.6.

#### 5.1.4 Synthesis of racemic *H*-phosphinic analogs of Hcy, Pro-S-Hcy-P_H_, SAH, and SAM

##### 5.1.4.1 1-Amino-3-mercaptopropyl-*H*-phosphinic acid (*rac-*Hcy-P_H_)

Sodium metal (2.53 g, 0.11 mol) was added in small pieces to a solution of *rac-*Bn-S-Hcy-P_H_ (12.3 g, 0.05 mol) in boiling liquid NH_3_ (250 mL) until the blue color remained unchanged for 10 min. The reaction mixture was stirred for 30 min, and then, solid NH_4_Cl was added until the disappearance of the blue color. The ammonia was allowed to evaporate, and the residue was co-evaporated several times with oxygen-free H_2_O *in vacuo*. The residue was dissolved in oxygen-free 15% aq. *i*-PrOH, and Hcy-P_H_ was purified on a Dowex 50W-X8 resin (H^+^-form, V = 500 mL) eluting with oxygen-free 15% aq. *i*-PrOH. Fractions containing *rac-*Hcy-P_H_ were concentrated *in vacuo*, and the residue was dried *in vacuo* over P_2_O_5_ to give *rac-*Hcy-P_H_ (3.63 g, 47%) as a colorless solid, *R*_f_ 0.39 (A), *R*_f_ 0.23 (B). Determination of the thiol groups with Ellman reagent (Ellman, 1959) gave 98.7% yield of the theoretical value.

##### 5.1.4.2 1-Amino-3-(propargylthio)propylphosphinic acid (*rac-*Pro-S-Hcy-P_H_)

A solution of propargyl chloride (300 mg, 4 mmol) in MeOH (4 mL) was added to the solution of *rac-*Hcy-P_H_ (330 mg, 2 mmol) in the mixture of MeOH (16 mL) and 2 M NaOH (4 mL). The reaction was left for 72 h at 20°C under Ar atmosphere and then concentrated *in vacuo*. The residue was dissolved in 15% aq. *i*-PrOH (5 mL) and purified on a Dowex 50W-X8 resin (H^+^-form, V = 50 mL) eluting with 15% aq. *i*-PrOH. Fractions containing *rac-*Pro-S-Hcy-P_H_ were concentrated *in vacuo*, the residue was recrystallized from aq. *i*-PrOH and dried *in vacuo* over P_2_O_5_ to yield *rac-*Pro-S-Hcy-P_H_ (270 mg, 67%). **ESI-MS (m/z):** expected mass for C_6_H_13_NO_2_PS^+^ = 194.0399 [M + H]^+^, found: 194.0397. Expected mass for C_6_H_11_NO_2_PS^−^ = 192.0254 [M-H]^-^, found: 192.0252. ^**1**^ **H NMR** (600 MHz, D_2_O) δ = 7.0 (dd, *J* = 535.2, 1.4, 1H), 3.4 (dd, *J* = 2.7, 1.1, 2H), 3.4–3.3 (m, 1H), 3.0 (ddd, *J* = 14.1, 8.5, 5.9, 1H), 2.9 (ddd, *J* = 13.9, 8.2, 6.9, 1H), 2.7 (t, *J* = 2.6, 0H), 2.3–2.2 (m, 1H), and 2.0 (d, *J* = 534.7, 1H). ^**13**^ **C NMR** (151 MHz, D_2_O) δ = 83.1, 74.9, 52.0 (d, *J* = 91.2), 29.9 (d, *J* = 9.7), 28.4, and 20.7 ^**31**^ **P NMR** (243 MHz, D_2_O) δ = 20.2.

##### 5.1.4.3 5′-[3-Amino-3-(hydrohydroxyphosphoryl)propylthio]-5′-deoxyadenosine (*rac-*SAH-P_H_)

To a solution of *rac-*Hcy-P_H_ (0.89 g, 5.75 mmol) in 2 M NaOH (5.8 mL) were subsequently added freshly distilled DMSO (12 mL), maintaining at 20°C, and a solution of 5′-deoxy-5′chloroadenosine (1.0 g, 3.5 mmol) in freshly distilled DMSO (12 mL). The reaction mixture was incubated for 3 days at 20°C and pooled into water (150 mL). The resulting solution was applied on a Dowex 50W-X8 column (H^+^-form, V = 55 mL), and it was washed with water and then with 1 M NH_4_OH to obtain *rac-*SAH-P_H_ contaminated with some (Hcy-P_H_)_2_. Fractions containing crude *rac-*SAH-P_H_ were concentrated *in vacuo*, dissolved in 1% aq. Py, applied on a Dowex 50W-X8 column (Py-form, V = 250 mL), and eluted with 1% aq. Py. Fractions containing *rac-*SAH-P_H_ were evaporated to dryness *in vacuo* and co-evaporated *in vacuo* several times with water and then with EtOH. The residue was dried *in vacuo* over P_2_O_5_, ground with abs. EtOH, and filtered and dried *in vacuo* over P_2_O_5_ to afford *rac-*SAH-P_H_ (1.17 g, 83%). An additional amount of pure *rac-*SAH-P_H_ (0.13 g, 9%) was obtained from the mother liquid, *R*_f_ 0.47 (A), *R*_f_ 0.17 (B), and *R*_f_ 0.26 (C). **ESI-MS (m/z):** expected mass for C_13_H_22_N_6_O_5_PS^+^ = 405.1105 [M + H]^+^, found: 405.1095. Expected mass for C_13_H_20_N_6_O_5_PS^−^ = 403.0959 [M-H]^-^, found: 403.0956. ^**1**^ **H NMR** (600 MHz, D_2_O) δ = 8.4 (s, 1H), 8.3 (s, 1H), 7.0 (d, *J* = 534.9, 1H), 6.1 (d, *J* = 4.9, 1H), 4.9 (t, *J* = 5.2, 1H), 4.5–4.4 (m, 1H), 4.4–4.3 (m, 1H), 3.3–3.3 (m, 1H), 3.1 (dt, *J* = 14.2, 4.7, 1H), 3.0 (ddd, *J* = 14.2, 6.9, 1.7, 1H), 2.9–2.7 (m, 2H), 2.2–2.1 (m, 1H), and 2.0–1.9 (m, 1H). ^**13**^ **C NMR** (151 MHz, D_2_O) δ = 155.4, 151.4, 151.3, 144.2, 121.5, 90.7, 86.3 (d, *J* = 18.7), 76.3 (d, *J* = 3.5), 75.1 (d, *J* = 9.6), 52.0 (d, *J* = 90.9), 36.1 (d, *J* = 7.8), 31.0 (d, *J* = 8.5), and 29.0. ^**31**^ **P NMR** (243 MHz, D_2_O) δ = 20.1.

##### 5.1.4.4 5′-{*S*-[3-Amino-3-(hydrohydroxyphosphoryl)propy]-*S*-methylthionia}-5′-deoxyadenosine hydrochloride (*rac-*SAM-P_H_)

Freshly distilled methyl iodide (1.5 mL, 24 mmol) was added to a stirred solution of *rac-*SAH-P_H_ (315 mg, 0.78 mmol) in 99.5% HCOOH (5 mL) and abs. dioxane (3 mL) containing anhydrous H_3_PO_2_ (100 mg, 1.5 mmol). The reaction mixture was stirred at 20°C in the dark for 2 days, concentrated *in vacuo*, and the residue was dissolved in 0.05 M HCl and applied on a Dowex 50W-X8 column (H^+^-form, V = 5.5 mL). The column was eluted subsequently with 0.5 M HCl and 1.0 M HCl, and *rac-* SAM-P_H_ was eluted with 2.0 M HCl. Fractions containing *rac-*SAM-P_H_ were evaporated to dryness *in vacuo* and co-evaporated *in vacuo* several times with water. The residue was dissolved in a minimal volume of boiling MeOH, and boiling EtOH was slowly added to the resulting solution until the beginning of crystallization. After 16 h at 4°C, crystals were filtered and dried *in vacuo* over P_2_O_5_/KOH to yield *rac-*SAM-P_H_ (220 mg, 62%). *R*_f_ 0.12 (A), *R*_f_ 0.04 (B), *R*_f_ 0.25 (C). **UV** (H_2_O): λ_max_/nm 259, λ_min_/nm 228. **ESI-MS (m/z):** expected mass for C_14_H_24_N_6_O_5_PS^+^ = 419.1261 M^+^, found: 419.1248. ^**1**^ **H NMR** (600 MHz, D_2_O) δ = 8.4 (s, 1H), 8.3 (s, 1H), 7.0 (d, *J* = 534.9, 1H), 6.1 (d, *J* = 4.9, 1H), 4.9 (t, *J* = 5.2, 1H), 4.5–4.4 (m, 1H), 4.4–4.3 (m, 1H), 3.3–3.3 (m, 1H), 3.1 (dt, *J* = 14.2, 4.7, 1H), 3.0 (ddd, *J* = 14.2, 6.9, 1.7, 1H), 2.9–2.7 (m, 2H), 2.2–2.1 (m, 1H), and 2.0–1.9 (m, 1H). ^**13**^ **C NMR** (151 MHz, D_2_O) δ = 153.2, 151.0–150.8 (m), 148.0–147.7 (m), 146.4–146.0 (m), 122.2, 92.9–92.7 (m), 81.9–81.1 (m), 76.1–75.8 (m), 75.8–75.4 (m), 51.7–50.9 (m), 47.4–46.4 (m), 42.3–41.5 (m), 26.8–25.8 (m), and 24.9–24.1 (m). ^**31**^ **P NMR** (243 MHz, D_2_O) δ = 18.2, 18.1.

#### 5.1.5 Synthesis of racemic phosphonic analogs of Met, ethionine, SAH, and SAM

##### 5.1.5.1 1-Amino-3-(methylthio) propylphosphonic acid (*rac-*Met-P_5_)

To the solution of *rac-*Met-P_H_ (1.69 g, 10 mmol) in 1 M aq. HI (20 mL) and EtOH (20 mL) at 40°C was added dropwise 0.4 M I_2_ in EtOH (totally ∼ 25.5 mL) until the slight yellow color remained unchanged within 15 min. The reaction mixture was concentrated *in vacuo*, and the residue was dissolved in 80% aq. EtOH (20 mL), neutralized with propylene oxide, and kept at 4°C for 4 h. The precipitated material was filtered, recrystallized from aq. EtOH, and dried *in vacuo* over P_2_O_5_ to yield *rac-*Met-P_5_ (1.65 g, 89%), mp 256°C–257°C (lit.: 270–272°С (Kudzin and Stec, 1980)). **ESI-MS (m/z):** expected mass for C_4_H_13_NO_3_PS^+^ = 186.0348 [M + H]^+^, found: 186.0344. Expected mass for C_4_H_11_NO_3_PS^−^ = 184.0203 [M-H]^-^, found: 184.02. ^**1**^ **H NMR** (600 MHz, D_2_O) δ = 3.5–3.4 (m, 1H), 2.8–2.6 (m, 2H), 2.3–2.2 (m, 1H), 2.1 (s, 3H), and 2.1–2.0 (m, 1H). ^**13**^ **C NMR** (151 MHz, D_2_O) δ = 50.7 (d, *J* = 142.8), 32.4 (d, *J* = 10.1), 30.3, and 16.5. ^**31**^ **P NMR** (243 MHz, D_2_O) δ = 13.8.

##### 5.1.5.2 1-Amino-3-(ethylthio) propylphosphonic acid (*rac-*ethionine-P_5_)

*rac-*Ethionine-P_5_ was obtained as described above for *rac-*Met-P_5_ starting from *rac-*Ethionine-P_H_ (1.83 g, 10 mmol). The recrystallization from aq. EtOH yielded *rac-*ethionine-P_5_ (1.7 g, 85%). **ESI-MS (m/z):** expected mass for C_5_H_15_NO_3_PS^+^ = 200.0505 [M + H]^+^, found: 200.05. Expected mass for C_5_H_13_NO_3_PS^−^ = 198.0359 [M-H]^-^, found: 198.0358. ^**1**^ **H NMR** (600 MHz, D_2_O) δ = 3.4 (ddd, J = 13.6, 8.5, 5.4, 1H), 2.8–2.7 (m, 2H), 2.6 (q, J = 7.4, 2H), 2.3–2.2 (m, 1H), 2.1–2.0 (m, 1H), and 1.2 (t, J = 7.4, 3H). ^**13**^ **C NMR** (151 MHz, D_2_O) δ = 50.8 (d, J = 142.5), 30.9, 29.8 (d, J = 10.0), 27.5, and 16.6. ^**31**^ **P NMR** (243 MHz, D_2_O) δ = 13.7.

##### 5.1.5.3 5′-[3-Amino-3-(hydroxyphosphoryl)propylthio]-5′-deoxyadenosine (*rac-*SAH-P_5_)

0.2 M I_2_ in EtOH was added dropwise at 50°C to the solution of *rac-*SAH-P_H_ (100 mg, 0.22 mmol) in 0.5 М HI (2.0 mL) and EtOH (1.0 mL) until the slight yellow color remained unchanged within 10 min (titration takes approximately 30–35 min). H_2_O was added to the reaction mixture, and the resulting solution was applied on a Dowex 50W-X8 column (H^+^-form, V = 4.0 mL). The column was washed with water, and the crude *rac-*SAH-P_5_ containing traces of *rac-*SAH-P_H_ was eluted with 2.5% NH_4_OH. The corresponding fractions were evaporated to dryness *in vacuo*, dissolved in MeOH-25% NH_4_OH (7:3) mixture (2.0 mL), and applied on a SiO_2_ column (35 g). *rac-*SAH-P_5_ was eluted with MeOH-25% NH_4_OH (7:3), the appropriate fractions were concentrated *in vacuo*, and the residue was co-evaporated several times with water and dried *in vacuo* over P_2_O_5_ to yield *rac-*SAH-P_5_ (72 mg, 78%), *R*_*f*_ 0.14 (A), *R*_*f*_ 0.03 (B), and *R*_*f*_ 0.20 (C). **ESI-MS (m/z):** Expected mass for C_13_H_22_N_6_O_6_PS^+^ = 421.1054 [M + H]^+^, found: 421.1043. Expected mass for C_13_H_20_N_6_O_6_PS^−^ = 419.0908 [M-H]^-^, found: 419.0907. ^**1**^ **H NMR** (600 MHz, D_2_O) δ = 8.4 (s, 1H), 8.3 (s, 1H), 6.1 (d, *J* = 5.0, 1H), 4.8 (t, *J* = 5.2, 1H), 4.4 (q, *J* = 4.7, 1H), 4.3 (q, *J* = 4.6, 1H), 3.4–3.4 (m, 1H), 3.1–3.0 (m, 1H), 3.0–2.9 (m, 1H), 2.9–2.7 (m, 2H), 2.3–2.2 (m, 1H), and 2.1–1.9 (m, 1H). ^**13**^ **C NMR** (151 MHz, D_2_O) δ = 156.7, 153.2, 151.4, 143.6, 121.5, 90.5, 86.6–85.6 (m), 76.2, 75.2–74.9 (m), 50.6 (d, *J* = 142.3), 36.1 (d, *J* = 8.2), and 31.3–30.9 (m). ^**31**^ **P NMR** (243 MHz, D_2_O) δ = 16.1–13.1 (m).

##### 5.1.5.4 5′-{*S*-[3-Amino-3-(hydroxyphosphoryl)propyl]-*S*-methylthionia}-5′-deoxyadenosine hydrochloride (*rac-*SAM-P_5_)

Freshly distilled methyl iodide (0.3 mL, 4.8 mmol) was added to a stirred solution of *rac-*SAH-P_5_ (60 mg, 0.14 mmol) in 99.5% HCOOH (0.75 mL) and abs. dioxane (0.75 mL) containing anhydrous H_3_PO_2_ (10 mg, 0.15 mmol). The reaction mixture was stirred at 20°C in the dark for 2 days and concentrated *in vacuo*. The residue was dissolved in 0.05 M HCl (1 mL) and applied on a Dowex 50W-X8 column (H^+^-form, V = 1.5 mL). The column was eluted subsequently with 0.5 M HCl and 1.0 M HCl, and SAM-P_5_ was eluted with 2.0 M HCl. Fractions containing *rac-*SAM-P_5_ were evaporated to dryness *in vacuo* and co-evaporated several times with water *in vacuo*. The residue was dried *in vacuo* over P_2_O_5_/KOH to yield *rac-*SAM-P_5_ (34 mg, 50%) and *R*_f_, 0.20 (C). **ESI-MS (m/z):** expected mass for C_14_H_24_N_6_O_6_PS^+^ = 435.121 [M^+^], found: 435.1202. ^**1**^ **H NMR** (600 MHz, D_2_O) δ = 8.5–8.5 (m, 2H), 6.2 (d, *J* = 3.8, 1H), 4.9–4.9 (m, 1H), 4.7–4.6 (m, 2H), 4.1–3.9 (m, 2H), 3.8–3.5 (m, 2H), 3.4–3.3 (m, 1H), 3.1–3.0 (m, 3H), 2.5–2.4 (m, 1H), and 2.4–2.2 (m, 1H). ^**13**^ **C NMR** (151 MHz, D_2_O) δ = 152.8, 150.8, 147.3 (d, *J* = 5.3), 146.3, 122.2, 92.8, 81.7 (d, *J* = 5.5), 81.1 (d, *J* = 15.4), 76.0–75.3 (m), 50.6–49.3 (m), 47.0–46.6 (m), 42.1–41.5 (m), and 26.8–25.7 (m). ^**31**^ **P NMR** (243 MHz, D_2_O) δ = 11.9–11.7 (m).

#### 5.1.6 Stability measurement of SAM, *rac-*SAM-P_H_, and *rac-*SAM-P_5_

The reaction mixtures (550 μL) contained SAM, *rac-* **SAM-P**_**H**_, or *rac-* **SAM-P**_**5**_ (4 mM), DSS-d_6_ as an internal standard (10 μL of 2 mg/mL stock), and Tris-d_11_ buffer (100 mM, pH 8.0) prepared on deuterated water. Immediately after preparation, the reaction mixtures were placed into the NMR spectrometer and incubated at 37°C. ^1^H NMR spectra were recorded every 2 min for the first 4 h and then after each 10–15 h (Supplementary Figure S2). The degradation of SAM or its analog was monitored by the decrease in the signals of its adenine part on NMR spectra.

The concentration was measured by the integration of NMR signals relative to the internal standard DSS-d_6_. The assay was repeated several times. The half-life time (t_1/2_) was determined using GraphPad Prism 9.5.0 (Supplementary Figure S1, GraphPad Software Inc., San Diego, CA, United States).

### 5.2 Biology

#### 5.2.1 Protein expression and purification

Protocols for protein expression, isolation, and purification of MAT2A, *At*HMT^V140T^, COMT, WBSCR27, and MTAN are presented in detail in the Supplementary Material.

#### 5.2.2 Enzymatic reactions

Protocols for carrying out enzymatic reactions of MAT2A, *At*HMT^V140T^, COMT, and MTAN are described in detail in the Supplementary Material.

### 5.3 NMR analysis of protein–ligand interactions

Interactions of WBSCR27 with an excess of SAM, *rac-* **SAM-P**_**H**_, and *rac-* **SAM-P**_**5**_ were studied using NMR titration experiments. Spectra were recorded on a Bruker Avance-700 MHz spectrometer equipped with a nitrogen cryoprobe (Bruker Corporation, Karlsruhe, Germany). ^15^N-labeled apo-WBSCR27 samples at 0.2 mM concentration were used. The ligand concentration increased from 1:1 to 1:5 protein–ligand ratio. For each titration point, a^15^N,^1^H HSQC spectrum was recorded (Supplementary Figures S15, S16).

The resonance assignments of WBSCR27 in complex with **SAM-P**_**H**_ and **SAM-P**_**5**_ were made based on previously published assignments for the apo-form of WBSCR27 (BMRB ID 27578) and the SAH-binding complex of WBSCR27 (BMRB ID 27417).

The chemical shift difference between the ligand-bound and apo forms was calculated using the following equation:

Δi=12·ΔδHi12+ΔδNi15225,

where Δδ(^1^H^*i*^) and Δδ(^15^N^*i*^) are the chemical shift differences for the H^N^ and N-atoms of amid group for each residue, respectively, between the complex and apo-forms.

Residues with a large chemical shift difference (Δ^i^ > 0.1) between the apo-form and the ligand-bound state were mapped onto the WBSCR27 methyltransferase structure using the PyMOL Molecular Graphics System version 3.0 from Schrödinger LLC.

## Data Availability

The original contributions presented in the study are included in the article/Supplementary Material; further inquiries can be directed to the corresponding author.
