# Supplementary material for: Organophosphorus S-adenosyl-L-methionine mimetics: synthesis, stability, and substrate properties
Source: Front Chem. 2024 Aug 1;12:1448747. doi: 10.3389/fchem.2024.1448747 (PMC11325224; doi:10.3389/fchem.2024.1448747)
Supplement: Supplementary file 1 [file DataSheet1.PDF]

Supplementary Material

Organophosphorus *S*-adenosyl-*L*-methionine mimetics: synthesis, stability, and substrate properties

Alexander Yu. Rudenko[1,2], Sofia S. Mariasina[1,2,3,4], Anastasia K. Bolikhova[1], Maxim V. Nikulin[1], Ratislav M. Ozhiganov[1,5], Vasiliy G. Vasil'ev[4], Yuri A. Ikhalaynen[3], Anastasia L. Khandazhinskaya[6], Maxim A. Khomutov[6], Peter V. Sergiev[1,3], Alex R. Khomutov[6], Vladimir I. Polshakov[2]*

[1] Belozersky Institute of Physico-Chemical Biology, M.V. Lomonosov Moscow State University, Moscow, Russia

[2]Faculty of Fundamental Medicine, M.V. Lomonosov Moscow State University, Moscow, Russia

[3]Chemical Department, M.V. Lomonosov Moscow State University, Moscow, Russia

[4]Research and Educational Resource Center "Pharmacy", RUDN University, Moscow, Russia

[5]Mendeleev University of Chemical Technology, Moscow, Russia

[6]Engelhardt Institute of Molecular Biology, Russian Academy of Sciences, Moscow, Russia

Table of Contents

1 Chemistry 3

1.1 Small-molecules synthesis 3

1.2 Stability measurement of SAM, *rac*-SAM-P_H and *rac*-SAM-P_5 4

2 Protein expression and purification........ 6

2.1 Plasmids for protein expression........ 6

2.2 Protein sequences........ 6

2.3 MAT2A, HMT, COMT: expression and purification........ 7

2.4 MTAN: expression and purification........ 8

2.5 ^{15}N-labelled WBSCR27: expression and purification........ 9

3 Enzymatic reactions 11

3.1 MAT2A catalyzes synthesis of *(R,S)*-SAM-P_H and and its S-substituted analogue. 11

3.2 HMT catalyzes synthesis of *(R,S)*-SAM-P_H and its *S*-substituted derivatives........ 12

3.3 Michaelis-Menten kinetics for *At*HMT^{V140T}-catalysed *(R,S)*-SAM-P_H synthesis 13

3.4 Preparative scale synthesis of *(R,S)*-SAM-P_H hydrochloride using *At*HMT^{V140T} 15

3.5 Preparative scale synthesis of *(R,S)*-SAM-P_H and *(S,S)*-SAM-P_H mixture using *At*HMT^{V140T}........ 17

3.6 NMR study of COMT catalyzed methylation of protocatechuic aldehyde by phosphorus-containing mimetics of SAM........ 21

3.7 NMR analysis of protein-ligand interactions 23

3.8 The MTAN catalyzed degradation of organophosphorus mimetics of SAH studied by HPLC 25

4 NMR spectra of small-molecules........ 29

5 HR-ESI mass spectra of small molecules........ 70

6 References 78

1 Chemistry

1.1 Small-molecules synthesis

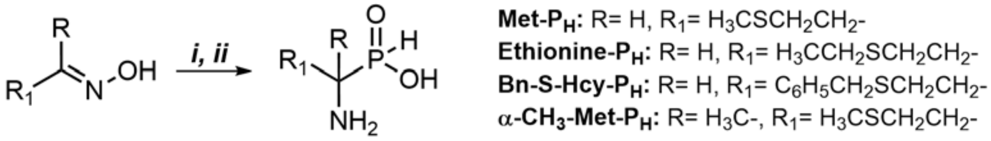

Supplementary Scheme S1. ***i-*** H_3PO_2/ROH/Δ; ***ii-*** Dowex 50W-X8, H^+-form, elution with 15% aqueous *i*-PrOH

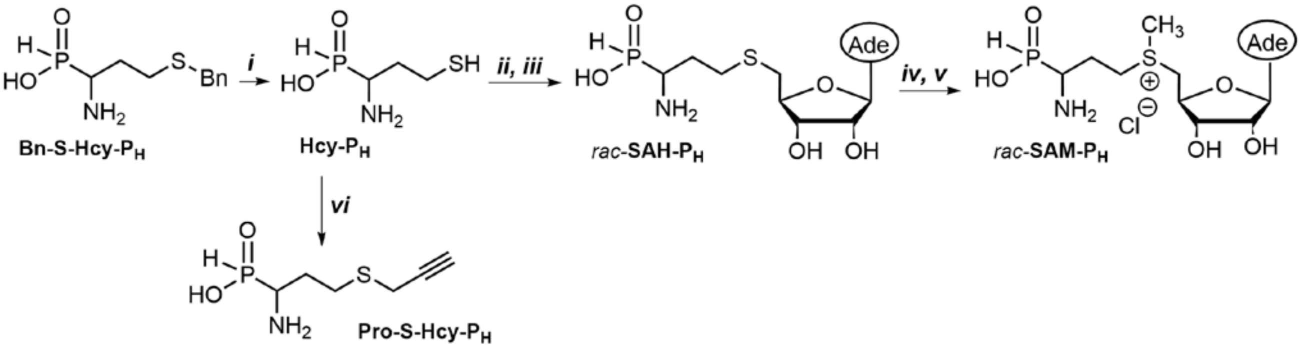

Supplementary Scheme S2. ***i-*** Na/liquid. NH_3; ***ii-*** 5'-Cl-adenosine/DMSO/NaOH/H_2O; ***iii-*** Dowex 50W-X8, H^+-form, elution with 2.5% NH_4OH, then Dowex 50W-X8, Py^+-form, elution with 1.0% aqueous Py; ***iv-*** MeI/HCOOH/dioxane; ***v-*** Dowex 50W-X8, H^+-form, stepwise elution with 0.5 M HCl, 1.0 M HCl, and 2.0 M HCl; ***vi-*** Pro-Cl/MeOH/NaOH/H_2O.

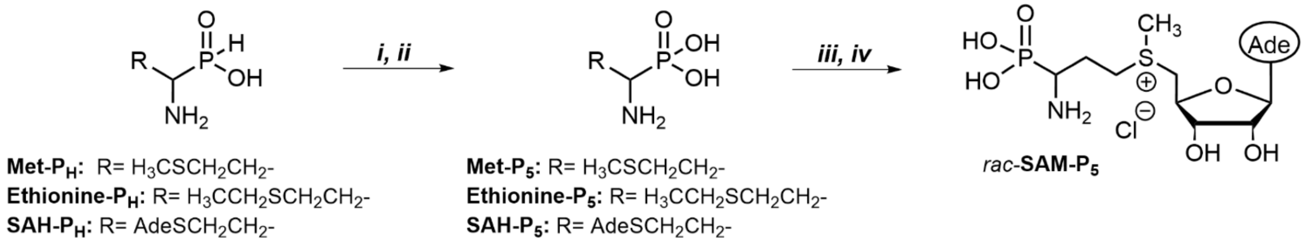

Supplementary Scheme S3. ***i-*** I_2/HI/EtOH/H_2O/40-50°C; ***ii-*** A) crystallization for Met-P_5 and Et-P_5, B) Dowex 50W- X8, H^+-form, elution with 2.5% NH_4OH, then Kieselgel 40-63 μm, elution with MeOH-25% NH_4OH = 7:3; ***iii-*** MeI/HCOOH/ dioxane; ***iv-*** Dowex 50W-X8, H^+-form, stepwise elution with 0.5 M HCl, 1.0 M HCl, and 2.0 M HCl.

1.2 Stability measurement of SAM, *rac*-SAM-P_H and *rac*-SAM-P_5

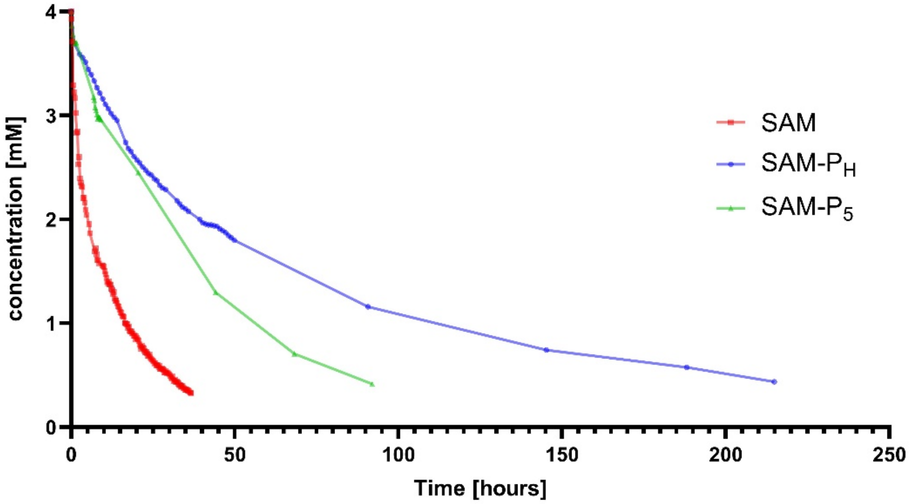

Supplementary Figure S1. Stability of SAM, *rac*-**SAM-P_H**, and *rac*-**SAM-P_5** in 100 mM Tris-d_{11} (D_2O), pH 8.0, and 37°C.

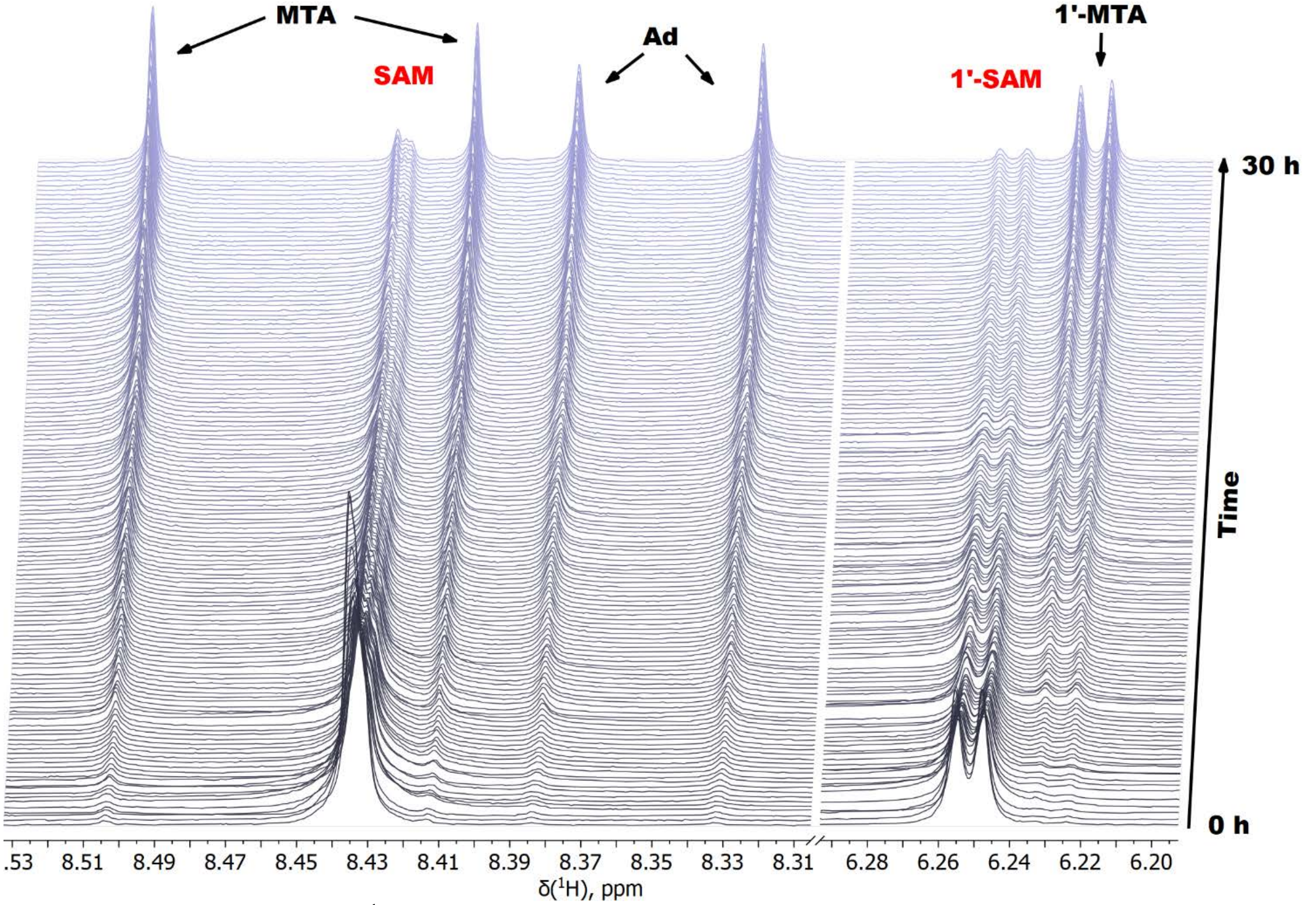

Supplementary Figure S2. ^{1}H NMR spectra of SAM degradation reaction in 100 mM Tris-d_{11} (D_2O), pH 8.0, 37°C.

2 Protein expression and purification

2.1 Plasmids for protein expression

To produce proteins, the following plasmids were used (**Supplementary Table S1**).

Supplementary Table S1. Plasmids used for protein expression.

Short name	Description	Vector	UniProt ID
MAT2A	Human MAT2A (Addgene #164820)	Modified pET	B4DEX8
*At*HMT^{V140T}	Mutant Halide methyltranserase HMT form *Arabidopsis thaliana*	pET-28a(+)	Q0WP12
COMT	Human catechol O-methyltransferase, COMT cysteine-free mutant C33S, C69V, C95S, C157S, C173S, C188R and C191A	pET-28a(+)	P21964
WBSCR27	*Mus Musculus* WBSCR27, T2A	pET30aTEV	Q99LP4
MTAN	5'-methylthioadenosine/S-adenosylhomocysteine nucleosidase from *Escherichia coli*	pCA24N-pfs	P0AF12

2.2 Protein sequences

The protein sequences used in this study are listed below.

Residues from affinity tags or modified ones are coloured in grey.

> MAT2A

*MHHHHHHSSGVDLGTENLYFQS*MNGQLNGFHEAFIEEGTFLFTSESVGEGHPDKICDQISDAV
LDAHLQQDPDAKVACETVAKTGMILLAGEITSRAAVDYQKVVREAVKHIGYDDSSKGFDY
KTCNVLVALEQQSPDIAQGVHLDRNEEDIGAGDQGLMFGYATDETEECMPLTIVLAHKLNA
KLAELRRNGTLPWLRPDSKTQVTVQYMQDRGAVLPIRVHTIVISVQHDEEVCLDEMRDALK
EKVIKAVVPAKYLDEDTIYHLQPSGRFVIGGPQGDAGLTGRKIIVDTYGGWGAHGGGAFSG
KDYTKVDRSAAYAARWVAKSLVKGGLCRRVLVQVSYAIGVSHPLSISIFHYGTSQKSERELL
EIVKKNFDLRPGVIVRDLDLKKPIYQRTAAYGHFGRDSFPWEVPKKLKY

> *At*HMT^{V140T}

M*GHHHHHHAENLYFQGSGS*AEEQQNSDQSNGGNVIPTPEEVATFLHKTVEEGGWEKCWEEE
ITPWDQGRATPLIVHLVDTSSLPLGRALVPGCGGGHDVVAMASPERFVVGLDISESALAKAN
ETYGSSPKAEYFSFVKEDVFTWRPTELFDLIFDYVFFCAIEPEMRPAWAKSMYELLKPDGELI
TLMYPITDHVGGPPYKVDVSTFEEVLVPIGFKAVSVEENPHAIPTRKGKEKLGRWKKIN

>COMT no_cys mutant

M*GHHHHHHENLYFQ*GDTKEQRILNHVLQHAEPGNAQSVLEAIDTY*S*EQKEWAMNVGDKK
GKIVDAVIQEHQPSVLLELGAY*V*GYSAVRMARLLSPGARLITIEINPD*S*AAITQRMVDFAGVK
DKVTLVVGASQDIIPQLKKKYDVDTLDMVFLDHWKDRYLPDTLLLEE*S*GLLRKGTVLLADN
VI*S*PGAPDFLAHVRGSS*R*FE*A*THYQSFLEYREVVDGLEKAIYKGPGSEAGP

> WBSCR27

*GA*M*A*QEEAGRLPQVLARVGTSHGITDLACKLRFYDDWAPEYDQDVAALKYRAPRLAVDCL
SRAFRGSPHDALILDVACGTGLVAVELQARGFLQVQGVDGSPEMLKQARARGLYHHLSLCT
LGQEPLPDPEGTFDAVIIVGALSEGQVPCSAIPELLRVTKPGGLVCLTTRTNPSNLPYKETLEA
TLDSLERAGVWECLVTQPVDHWELATSEQETGLGTCANDGFISGIIYLYRKQETV

> MTAN

MRGSHHHHHHKIGIIGAMEEEVTLLRDKIENRQTISLGGCEIYTGQLNGTEVALLKSGIGKVA
AALGATLLLEHCKPDVIINTGSAGGLAPTLKVGDIVVSDEARYHDADVTAFGYEYGQLPGCP
AGFKADDKLIAAAEACIAELNLNAVRGLIVSGDAFINGSVGLAKIRHNFPQAIAVEMEATAIA
HVCHNFNVPFVVVRAISDVADQQSHLSFDEFLAVAAKQSSLMVESLVQKLAHG

2.3 MAT2A, HMT, COMT: expression and purification

Protein expression:

For the expression of MAT2A, HMT and COMT proteins the *E. coli* BL21 (DE3) chemocompetent cells were transformed with the corresponding plasmid (Table S1). Precultures of 5 mL LB media containing 50 µg/mL kanamycin was inoculated by 1 colony of *E. coli* cells and incubated at 37°C, 180 rpm overnight.

For the main culture, 0.5 L of LB-media containing 50 mg/L kanamycin was inoculated from the overnight preculture and incubated at 37°C, 180 rpm until OD_{600} reached 0.6-0.7. Then the cells were cooled down to 17°C and expression was induced by adding isopropyl β-D-thiogalactoside (IPTG) to the final concentration of 1 mM. Protein expression was performed for 20 h at 17°C, 180 rpm. The cells were harvest by centrifugation, frozen and stored at -80°C until purification.

Purification of HMT and COMT:

Protein was purified via His-tag by immobilized metal affinity chromatography in native conditions. Cell pellets were resuspended in buffer A (20 mM sodium phosphate, 500 mM sodium chloride, and 20 mM imidazole, pH 7.5) and lysed by sonication. The cell debris was removed by two consecutive centrifugations each at 20,000×g for 30 min at 4°C. The supernatant was applied on the column containing 2 mL of Nickel SepharoseTM 6 Fast Flow (GE Healthcare, Sweden). The column was washed 5 times with 10 mL of buffer A. Proteins bound to the affinity sorbent were eluted with the same buffer containing 200 mM imidazole. The purification procedure was monitored by SDS-PAGE followed by Coomassie Blue staining. The selected fractions contained only target protein band. It was combined and dialyzed 6 times sequentially against 50 mM phosphate buffer pH 7.5 (each round lasting 6 hours) and stored in the same buffer containing 5% glycerol at 4°C. Protein concentrations were determined by measuring absorbance at 280 nm using a NanoDropTM (Thermo Fisher, Hennigsdorf, Germany). Extinction coefficients (280 nm) for all proteins are given in the section Protein sequences. Protein purities were analyzed by SDS-PAGE.

Purification of MAT2A:

In case of MAT2A the purification protocol was similar to that for HMT and COMT with the exception of the buffers. Elution was performed by Tris-HCl (25 mM, pH 8) and imidazole (300 mM). Dialysis was performed 3 times with Tris-HCl (25 mM, pH 8). Protein was concentrated and stored at −80°C.

2.4 MTAN: expression and purification

5'-methylthioadenosine/S-adenosylhomocysteine nucleosidase from *Escherichia coli* (MTAN) was expressed using the E.coli K-12 AG1 cells from ASKA(-) collection.(Kitagawa et al., 2006)

Glycerol stock JW0155 (pfs) was used to inoculate 5 mL of LB medium supplemented with 30 μg/ml chloramphenicol and incubated for ∼18 h at 37°C with shaking. A 5 mL aliquot was used to inoculate 800 mL of the same medium. When the A_{600} reached 0.4, MTAN expression was induced with 1 mM of IPTG. Cells were incubated at 17°C for an additional 16 h, after which the mixture was pelleted by centrifugation (5.000 g for 10 min at 5°C).

Purification:

The cell pellet (2 mL) was resuspended in 8 mL of buffer **B** (2 x PBS, 10% glycerol, 0.5 mg/mL lysozyme, 10 mM 2-mercaptoethanol and 1 x cOmplete Protease Inhibitor Cocktail from Roche), and lysed by sonication. Cell debris was pelleted by centrifugation (18,000 × g for 20 min at 4°C). The clarified supernatant was loaded onto a column HIS-SelectTM 1.25 mL (Sigma). The column was washed by two volumes of wash buffer (50 mM NaH_2PO_4/Na_2HPO_4, pH 7.5, 300 mM NaCl, 20 mM imidazole) until the A_{280} of the eluent was zero. MTAN was eluted with an elution buffer (50 mM NaH_2PO_4/Na_2HPO_4, pH 7.5, 300 mM NaCl, 250 mM imidazole). It was combined and dialyzed 3 times sequentially against 50 mM phosphate buffer pH 7.0. Protein concentrations were determined by measuring absorbance at 280 nm using a NanoDropTM (Thermo Fisher, Hennigsdorf, Germany). Extinction coefficients (280 nm) for all proteins are given in the section Protein sequences. Protein purities were analyzed by SDS-PAGE.

2.5 ^{15}N-labelled WBSCR27: expression and purification

Protein expression:

^{15}N uniformly labelled WBSCR27 was expressed using a pET30aTEV vector containing a 6His-tag, an S-tag, and a TEV cleavage site at the N-terminus.(Mariasina et al., 2018) *E. coli* BL21 (DE3) cells were transformed with the pET30aTEV-WBSCR27 vector and preculture of 30 mL of ^{15}N-isotopically labelled M9 minimal media containing $^{15}NH_4Cl$ (1 g/L, Cambridge Isotope Laboratories Inc.) and 50 µg/mL kanamycin was inoculated by 1 colony of cells and cultivated at 37°C, 180 rpm overnight.

For the main culture, 3 L of ^{15}N-labelled M9 media was inoculated from the overnight preculture and incubated at 37°C, 180 rpm until OD_{600} reached 0.6. The expression was induced with 1 mM IPTG for 16 h at 17°C. The cells were harvested by centrifugation (around 12 mL of cell pellets was obtained) and stored at -20 C until purification in two 50 mL tubes.

Purification in denaturation conditions:

As previously shown(Mariasina et al., 2020), when expressed in *E. coli*, WBSCR27 is co-purified as a complex with SAM and/or SAH. To remove endogenous SAM and SAH bound to WBSCR27, we purified protein in denaturing conditions with the following refolding during dialysis procedure. The refolding of protein to its native structure was approved using 2D NMR.

The frozen pellets were resuspended in 60 mL (30 mL in each tube) of a chilled buffer **B** (2 x PBS, 10% glycerol, 0.5 mg/mL lysozyme, 10 mM 2-mercaptoethanol and 1 x cOmplete Protease Inhibitor Cocktail from Roche). The cells were then lysed by sonication and all proteins were denatured by adding solid urea to the final concentration of 6 M. The cell debris was removed by centrifugation at 20,000×g for 15 min and washed 2 times by buffer B (10 and then 5 mL, each time followed by 20 min centrifugation). The supernatant combined with washed fractions was applied on the column containing 5 mL of Nickel Sepharose™ 6 Fast Flow (GE Healthcare, Sweden). The column was washed 5 times by 10 mL of buffer A. The WBSCR27 protein bound to the affinity sorbent was eluted with buffer B containing 350 mM imidazole. The purification procedure was monitored by PAGE (Supplementary Figure S3).

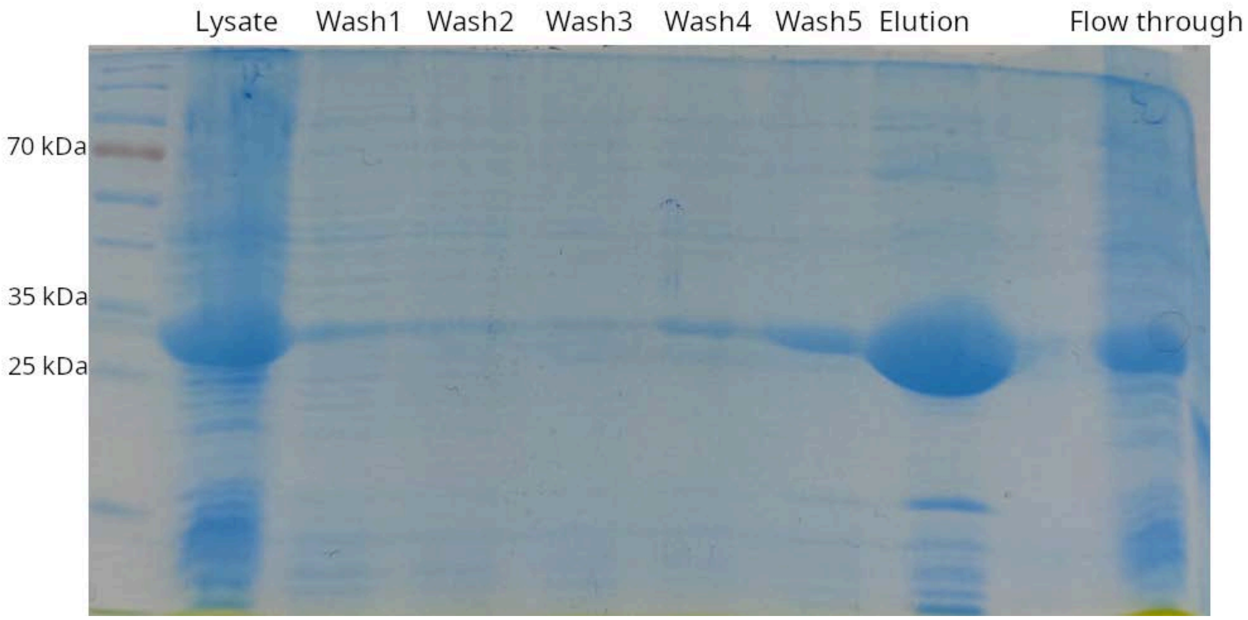

Supplementary Figure S3. SDS-PAGE analysis of ^{15}N labelled WBSCR27 purification in denaturing conditions.

The fractions containing WBSCR27 protein were combined, dialyzed against buffer B, and then the 6His-S-tag was cleaved with His-tagged TEV protease (1:100 by mass accessed on A_{280}) for 14 h at 4°C. Untagged WBSCR27 was separated from TEV protease and cleaved 6His-S-tag using column with 1 mL of Nickel Sepharose™. The final construct contained residues 1–238 of WBSCR27 and 2 residues from TEV cleavage site at N-terminus. The purified protein was concentrated using Amicon Ultra-15 centrifugal units (3 kDa cut-off, 40 min, 5000 g, 4°C) up to 15 mL. The concentrated protein was then 2 times dialyzed overnight against 1 L of buffer containing 8.3 mM sodium phosphate, 8.3 mM NaCl, 1.7 mM DTT, and 0.0033% NaN_3, at pH 7.0 and 4°C. The final protein concentration was 0.1 mM (ε_{280}=26), the total yield was 2.1 µmol (54.7 mg) of ^{15}N WBSCR27.

The protein sample was aliquoted by 960 µL and freeze-died for long-term storage at -60°C. Before NMR measurements, the dried powder was dissolved in 320 µL of mQ water to get sample containing 0.3 mM ^{15}N WBSCR27 in 25 mM NaCl, 25 mM sodium phosphate, 4 mM of DTT, 0.01% NaN_3 at pH 7.0.

3 Enzymatic reactions

3.1 MAT2A catalyzes synthesis of *(R,S)*-SAM-P_H and and its S-substituted analogue

The reaction mixtures (550 µL) contained methionine or its phosphorus-containing analogue (5 mM, Fig. S4), ATP (1 mM), hMAT2A (50 µM), $MgCl_2$ (10 mM), KCl (50 mM), DSS-d_6 as an internal standard (10 µL of 2 mg/ml stock), TRIS-d_{11} buffer (25 mM, pH 8.0) and deuterated water (10 vol %) for the frequency lock.

The reaction mixtures were incubated at 37°C in the NMR spectrometer and the ^{1}H NMR spectra were recorded 20 min, 1, 2, 12 and 24 h after the start of the reaction. The noesypr1d pulseprogram was used for water suppression. The appearance of the target compound was monitored by the rise of its signals on NMR spectra.

The concentration of product was measured by integration of NMR signals relative to the internal standard DSS-d_6. The assays were repeated twice, the average yields (in the case of *rac*-Met-P_H and *rac*-Ethionine-P_H yields are calculated for racemic analogue) are represented on Supplementary Figure S4.

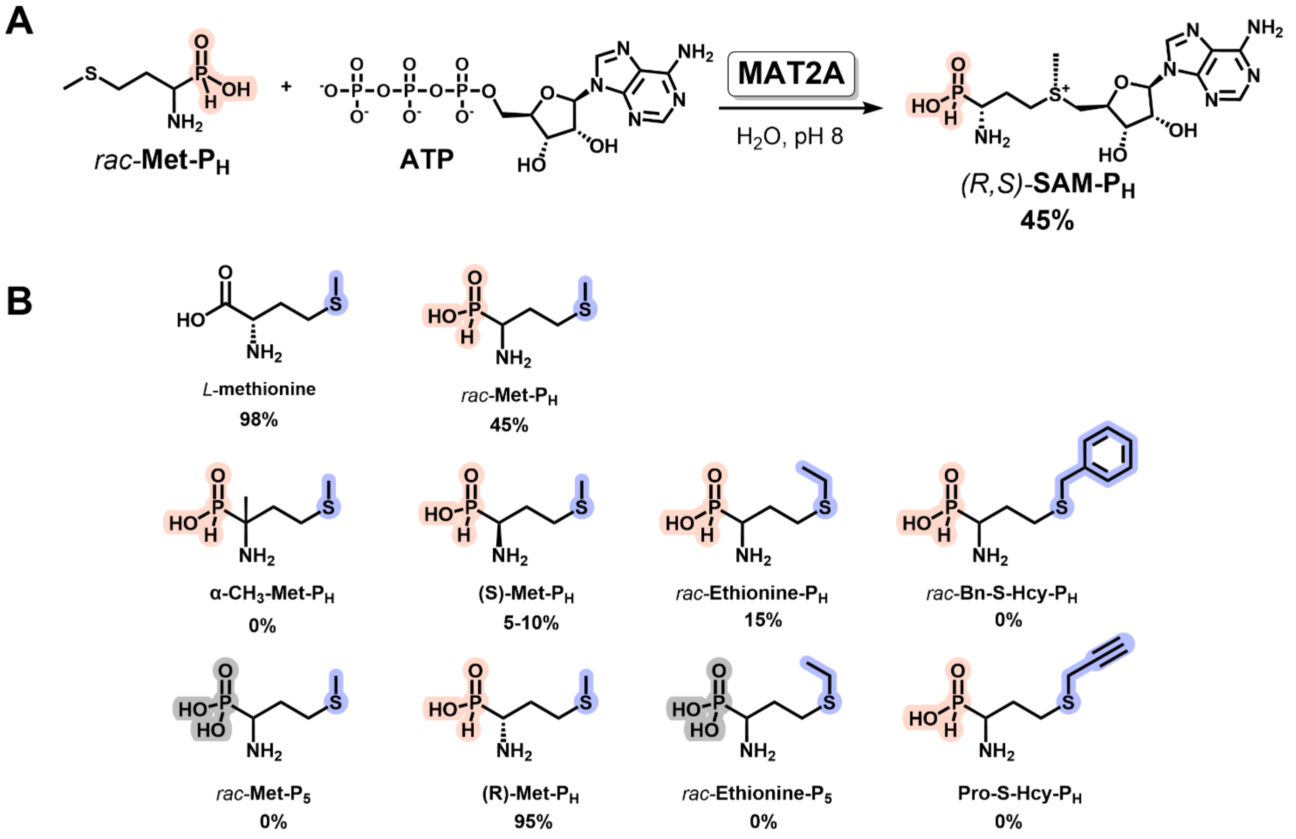

Supplementary Figure S4. MAT2A-catalyzed synthesis of *(R,S)*-**SAM-P_H** and its analogues. A. Synthesis of *(R,S)*-**SAM-P_H** from *rac*-**Met-P_H** and ATP. B. *H*-Phosphinic and phosphonic analogues of methionine as substrates of MAT2A. Reaction conditions: methionine, *H*-phosphinic and phosphonic analogues of methionine 5 mM, 1 mM ATP, 50 µM MAT2A, 50 mM KCl, 10 mM $MgCl_2$, 25 mM Tris-d_6 pH 8.0, 37°C.

3.2 HMT catalyzes synthesis of *(R,S)*-SAM-P_H and its *S*-substituted derivatives

The reaction mixtures (550 µL) contained SAH, *rac*-**SAH-P_H** or *rac*-**SAH-P_5** (2 mM), alkyl iodide (20 mM, stock in DMSO or acetonitrile), *At*HMT^{V140T} (40 µM), DSS-d_6 as an internal standard (10 µL of 2 mg/ml stock), chloride-free potassium phosphate buffer (50 mM, pH 7.5) and deuterated water (10 vol %) for the frequency lock (Supplementary Figure S5).
The reaction mixtures were incubated at 25 or 37°C in the NMR spectrometer and the ^{1}H NMR spectra were recorded 20 min, 1, 2, 12 and 24 h after the start of the reaction. The noesypr1d pulseprogram was used for water suppression. The appearance of the target compound was monitored by the rise of its signals on NMR spectra. The concentration of product was measured by integration of NMR signals relative to the internal standard DSS-d_6. The assays were repeated twice, the average yields (as calculated for *rac*-SAH-P_H) are represented on Supplementary Figure S5.

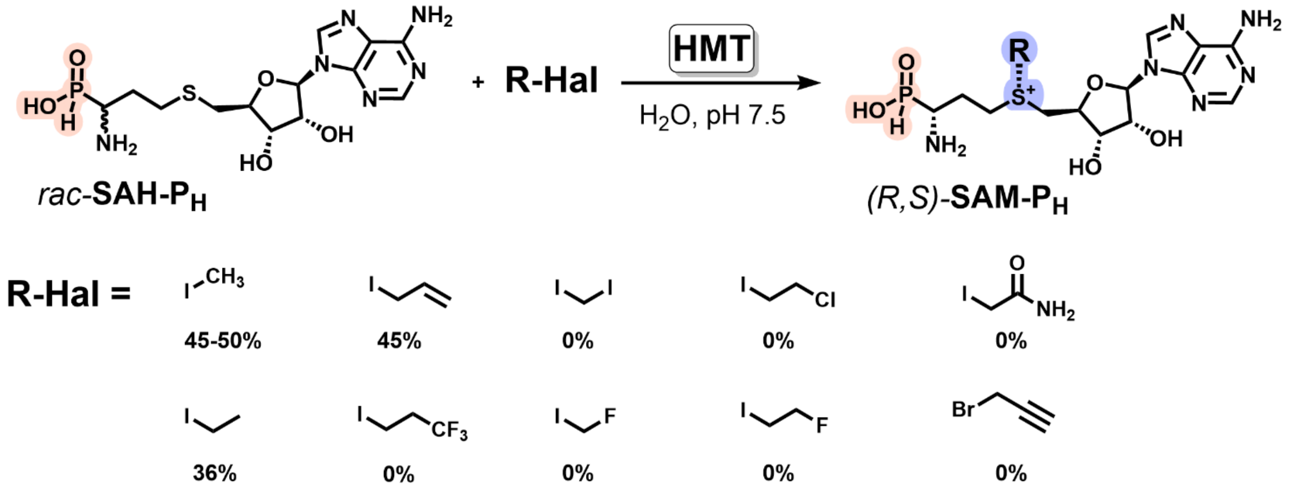

- **Supplementary Figure S5.** *At*HMT^{V140T}-catalyzed synthesis of **SAM-P_H** derivatives from *rac*-**SAH-P_H** and alkyl iodides. Reaction conditions: 2 mM *rac*-**SAH-P_H**, 20 mM alkyl iodides, 40 µM AtHMT^{V140T}, 50 mM chloride-free potassium phosphate buffer, pH 7.5, 37°C.
-

3.3 Michaelis-Menten kinetics for *At*HMT^{V140T}-catalysed *(R,S)*-SAM-P$_H$ synthesis

The kinetics of HMT catalyzed reactions of *rac*-**SAH-P$_H$** methylation was studied by HPLC using the protocol described earlier.(Tang et al., 2021) The reactions were carried out in 96-well plates. Reaction mixtures contained *rac*-**SAH-P$_H$** (1 mM), *At*HMT^{V140T} (11 µM), methyl iodide (0.5-10 mM, stock in DMSO) and chloride-free potassium phosphate buffer (50 mM, pH 7.5) for a final volume of 200 µL. Methyl iodide stocks in DMSO were freshly prepared for each experiment. The reagents were added in the following order: SAH-P$_H$, buffer, methyliodide, and then *At*HMT^{V140T}. Immediately after adding *At*HMT^{V140T}, the reaction mixtures were briefly mixed by pipetting and then after a certain time (15, 40, 80, 160, 300, or 600 sec) 20 uL of reaction mixture were taken and quenched by adding 60 µL of acetonitrile using 8 channel pipette. Thus obtained solutions (30 µL) were transferred to HPLC sample vial inserts, mixed with 970 µL of mQ water and used for HPLC analysis.

Analyses were performed on a Waters Breeze 2 HPLC System (Waters 1525 Binary HPLC Pump Waters 2489 UV/Visible Detector, Waters 2707 Autosampler) with a Kromasil Eternity C18 (4.6 x 250 mm column, 5 µm particle size) reversed-phase column (AkzoNobel, Ireland). For separation of SAH-P$_H$ and SAM-P$_H$, the mixture of 0.1% formic acid and acetonitrile 98:2 (v/v) was used as a mobile phase. The flow rate was 1 mL/min, injection volume – 20 µL, and column thermostat temperature – 30°C. SAH-P$_H$ and SAM-P$_H$, were detected at 260 nm and quantified using the internal normalization method. Retention time of SAM-P$_H$ was 2.5 min, and SAH-P$_H$ – 6.5 min (Supplementary Figure S6). Initial velocities (amount of iodide produced per min) were fit to the Michaelis-Menten model using GraphPad Prism 9.5.0 (GraphPad Software Inc., San Diego, CA, US) to determine the k_{cat} and K_m values (Supplementary Figure S7). All the reactions were performed in triplicate.

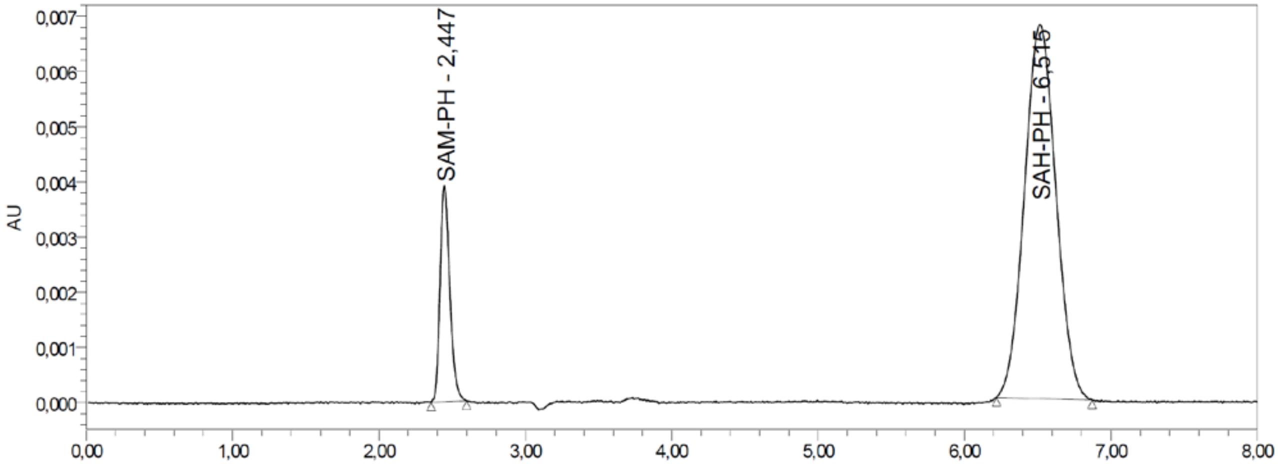

Supplementary Figure S6. Typical HPLC chromatogram of methyltransferase *At*HMT^{V140T} reaction mixture. Retention time of **SAM-P$_H$** is 2.5 min, **SAH-P$_H$** – 6.5 min.

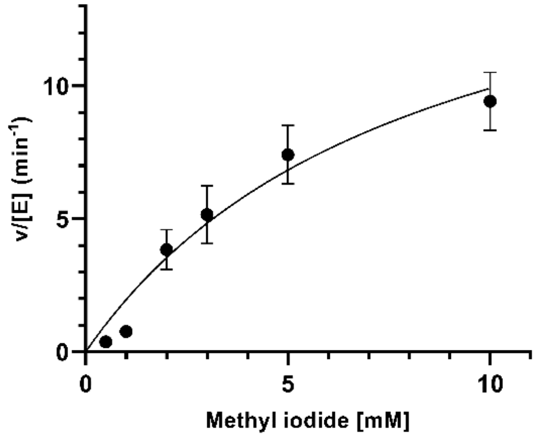

Supplementary Figure S7. Michaelis-Menten kinetics for *At*HMT^{V140T}-catalysed *(R,S)*-**SAM-P$_H$** synthesis. *rac*-**SAH-P$_H$** was used as a substrate. The measurements were made in triplicate. Data plotted are the means and standard deviations calculated from three independent measurements. The K_m value was 5.3 ± 3 mM, and K_{cat} value – 18 ± 2 min^{-1}.

3.4 Preparative scale synthesis of *(R,S)*-SAM-P$_H$ hydrochloride using *At*HMT^{V140T}

Reaction mixtures (25 mL) contained *rac*-**SAH-P$_H$** (39 mg), 0.20 mL of CH_3I/acetonitrile (5.0 M), *At*HMT^{V140T} (final concentration 0.8 mg/mL), chloride-free potassium phosphate buffer (50 mM, pH 7.5) and deuterated water (10 vol %) for the frequency lock. Reaction mixtures were incubated for 5 min at 25°C, and ^{1}H-NMR was recorded (Supplementary Figure S8). Then the reaction was initiated by addition of MTAN (2 μM). After 15 min, methanol (25 mL) was added and the obtained mixture was kept for 30 min at +4°C. The precipitants were separated by centrifugation and washed with 0.01 M HCl (2 x 1.5 mL). The supernatant was acidified with 37% HCl to pH ~ 2.0 and combined with the washings. The resulting solution was concentrated *in vacuo* to 10 mL and applied to a Dowex 50W-X8 column (H^+ form, V = 2.5 mL). The column was washed with 0.5 M HCl, 1.0 M HCl, and *(R,S)*-**SAM-P$_H$** was eluted by 2.0 M HCl. The fractions containing *(R,S)*-**SAM-P$_H$** were evaporated to dryness *in vacuo*, co-evaporated with water several times and dried *in vacuo* over P_2O_5/KOH to give *(R,S)*-**SAM-P$_H$** (22 mg, 46%) as a colourless solid.

^{1}H NMR (600 MHz, D_2O) δ = 8.4 (s, 1H), 8.4 (s, 1H), 6.9 (d, *J*=542.8, 1H), 6.1 (d, *J*=4.0, 1H), 4.9 – 4.8 (m, 1H), 4.6 – 4.5 (m, 1H), 4.6 – 4.5 (m, 1H), 4.0 (dd, *J*=13.8, 9.5, 1H), 3.9 (dd, *J*=13.8, 2.8, 1H), 3.7 – 3.6 (m, 1H), 3.6 – 3.5 (m, 1H), 3.3 – 3.2 (m, 1H), 3.0 (s, 3H), 2.4 – 2.3 (m, 1H), 2.3 – 2.1 (m, 1H).

^{13}C NMR (151 MHz, D_2O) δ = 152.6, 150.7, 147.1, 146.2, 122.0, 92.6, 81.0, 75.7, 75.2, 51.2 (d, *J*=92.7), 46.9, 41.5 (d, *J*=7.3), 26.1, 24.5.

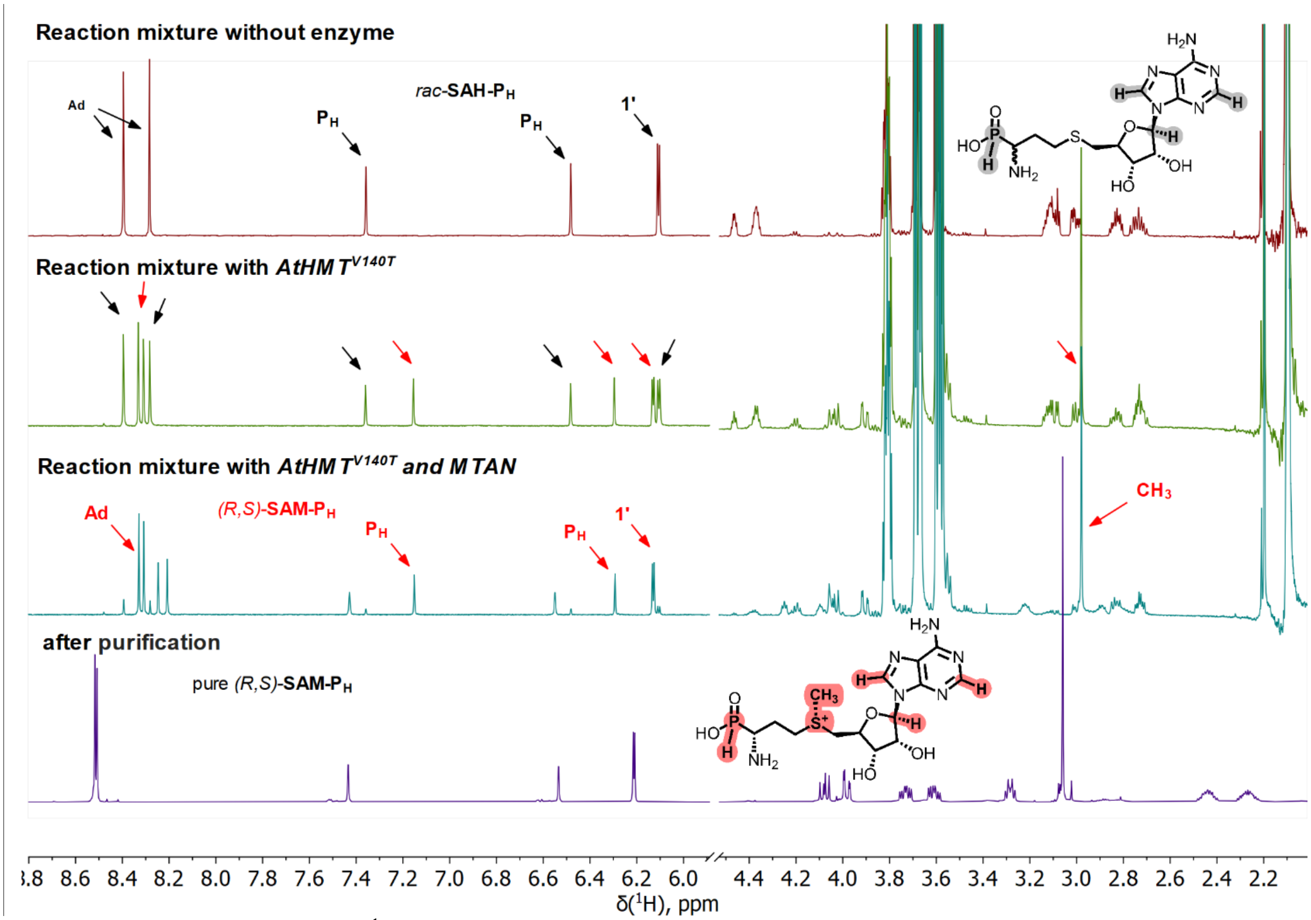

Supplementary Figure S8. ^{1}H NMR monitoring of the preparative synthesis of *(R,S)*-**SAM-P$_H$** using

the *At*HMT^{V140T}-catalyzed reaction. Spectra of reaction mixture are shown in the absence of enzymes, and in presence of *At*HMT^{V140T} or *At*HMT^{V140T}/MTAN. The spectrum at the bottom represents the purified product of the reaction. Rad arrows indicate the resonances of *rac*-**SAH-P$_H$**; black arrows indicate resonances of *(R,S)*-**SAM-P$_H$**.

3.5 Preparative scale synthesis of *(R,S)*-SAM-P_H and *(S,S)*-SAM-P_H mixture using *At*HMT^{V140T}

Reaction mixtures (25 mL) contained *rac*-**SAH-P_H** (57 mg), CH_3I/acetonitrile (0.25 mL, 5.0 M), *At*HMT^{V140T} (first up to 0.9 mg/mL and then to 2 mg/mL), chloride-free potassium phosphate buffer (50 mM, pH 7.5) and deuterated water (10 vol %) for the frequency lock. Reaction mixture was incubated for 20 min at 25°C (control by ^{1}H-NMR, Supplementary Figure S9, S10). Then methanol (25 mL) was added to the reaction mixture, and after 30 min at +4°C the precipitants were separated by centrifugation and washed with 0.01 M HCl (2 x 1.5 ml). The supernatant was adjusted with 37% HCl to pH ~ 2.0 and combined with the washings. The resulting solution was concentrated *in vacuo* to 10 mL and applied to a Dowex 50W-X8 column (H+ form, V = 2.5 ml). The column was eluted with 0.5 M HCl, 1.0 M HCl, and **SAM-P_H** was eluted with 2.0 M HCl. Fractions containing **SAM-P_H** were evaporated to dryness *in vacuo*, co-evaporated with water several times and dried *in vacuo* over P_2O_5/KOH to give mixture of diastereomers *(R,S)*-**SAM-P_H** and *(S,S)*-**SAM-P_H** (39 mg, 61%, Supplementary Figure S11) as a colourless solid.

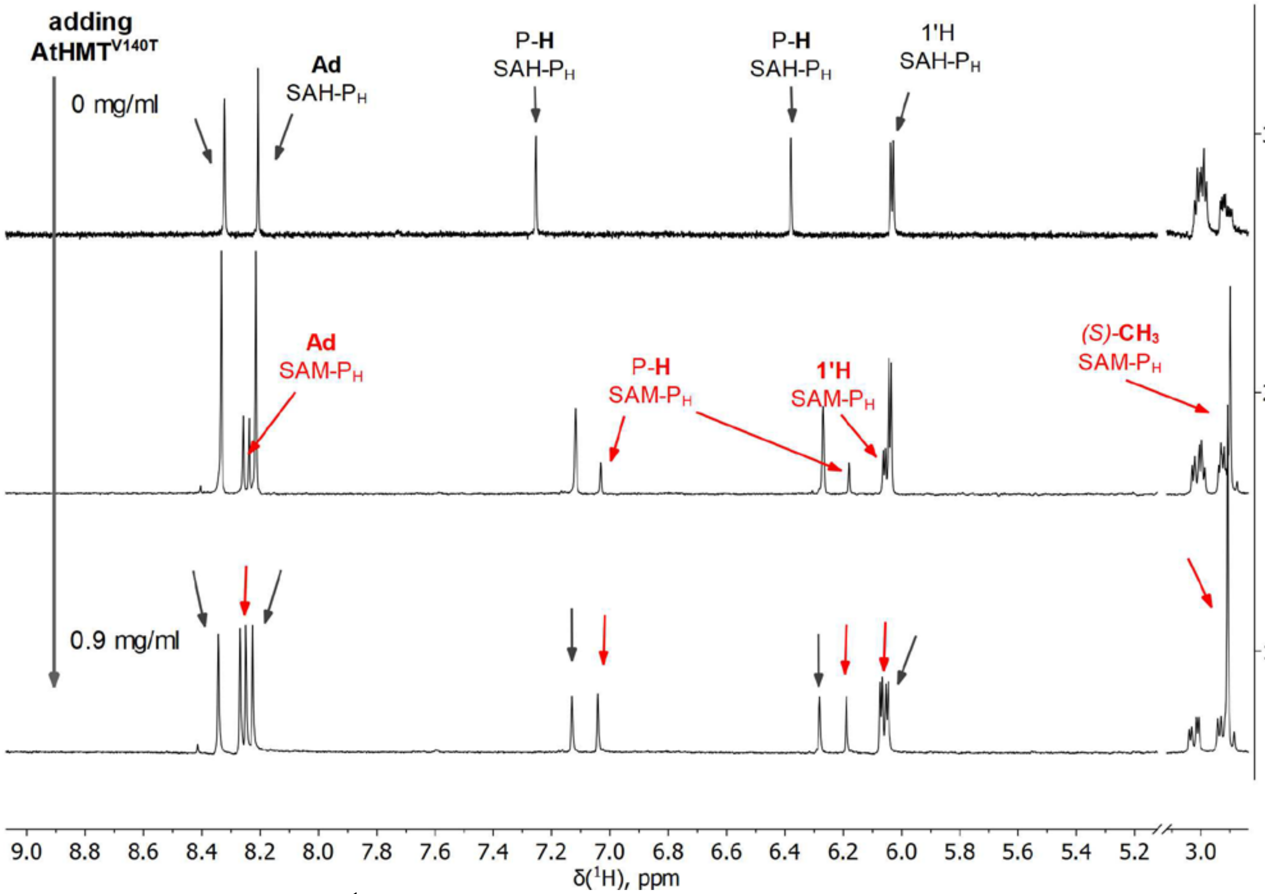

Supplementary Figure S9. ^{1}H NMR monitoring of the preparative enzymatic synthesis of **SAM-P_H.** The *At*HMT^{V140T} concentration increased up to 0.9 mg/mL. The final conversion of *rac*-**SAH-P_H** was 50% resulting in only *(R,S)*-**SAM-P_H**.

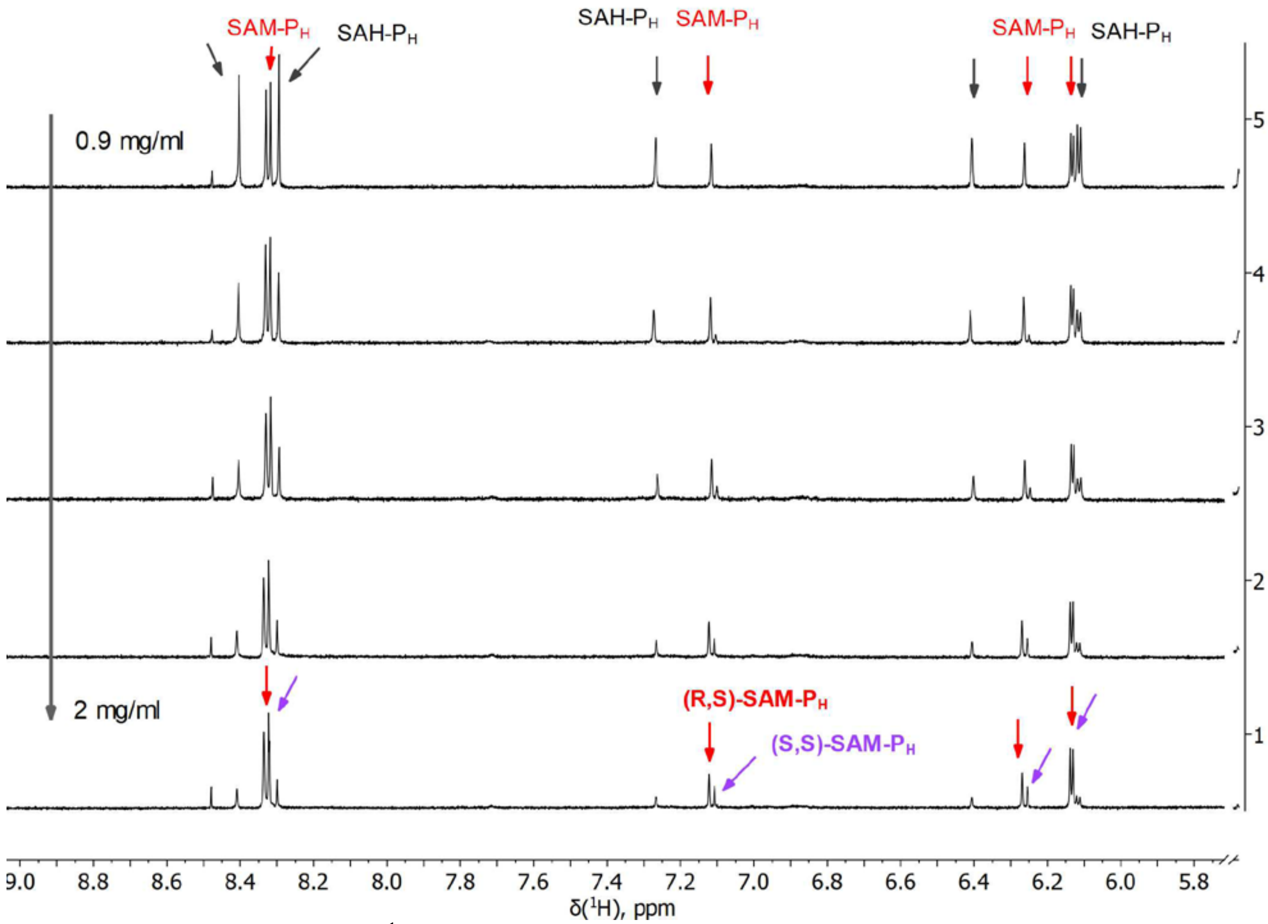

Supplementary Figure S10. ^{1}H NMR monitoring of the preparative enzymatic synthesis of **SAM-P$_{H}$.** The *At*HMT^{V140T} concentration increased up to 2 mg/mL. The final conversion of *rac*-**SAH-P$_{H}$** was more than 50% resulting in mixture of *(R,S)*-**SAM-P$_{H}$** and *(S,S)*-**SAM-P$_{H}$**.

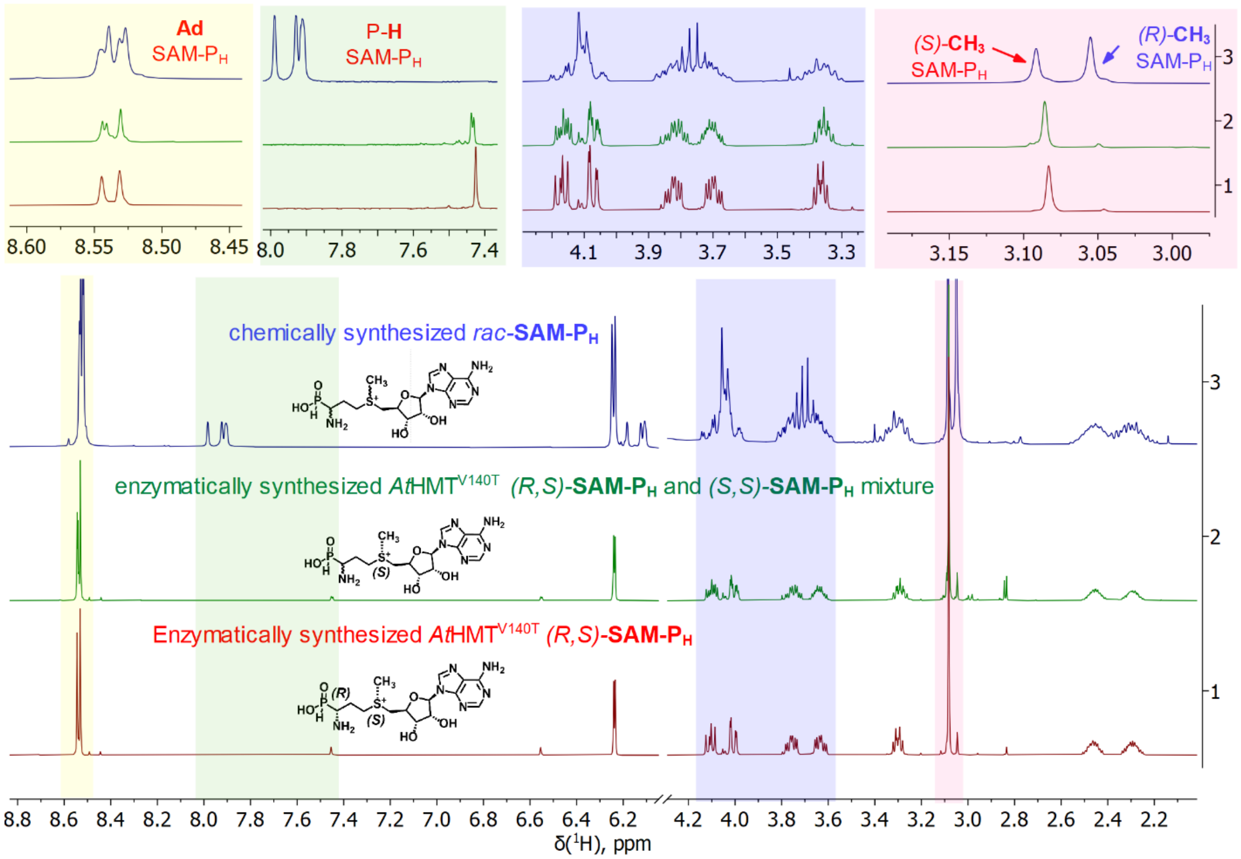

Supplementary Figure S11. ^{1}H NMR spectrum of various stereoisomers of the **SAM-P**$_{\mathbf{H}}$.

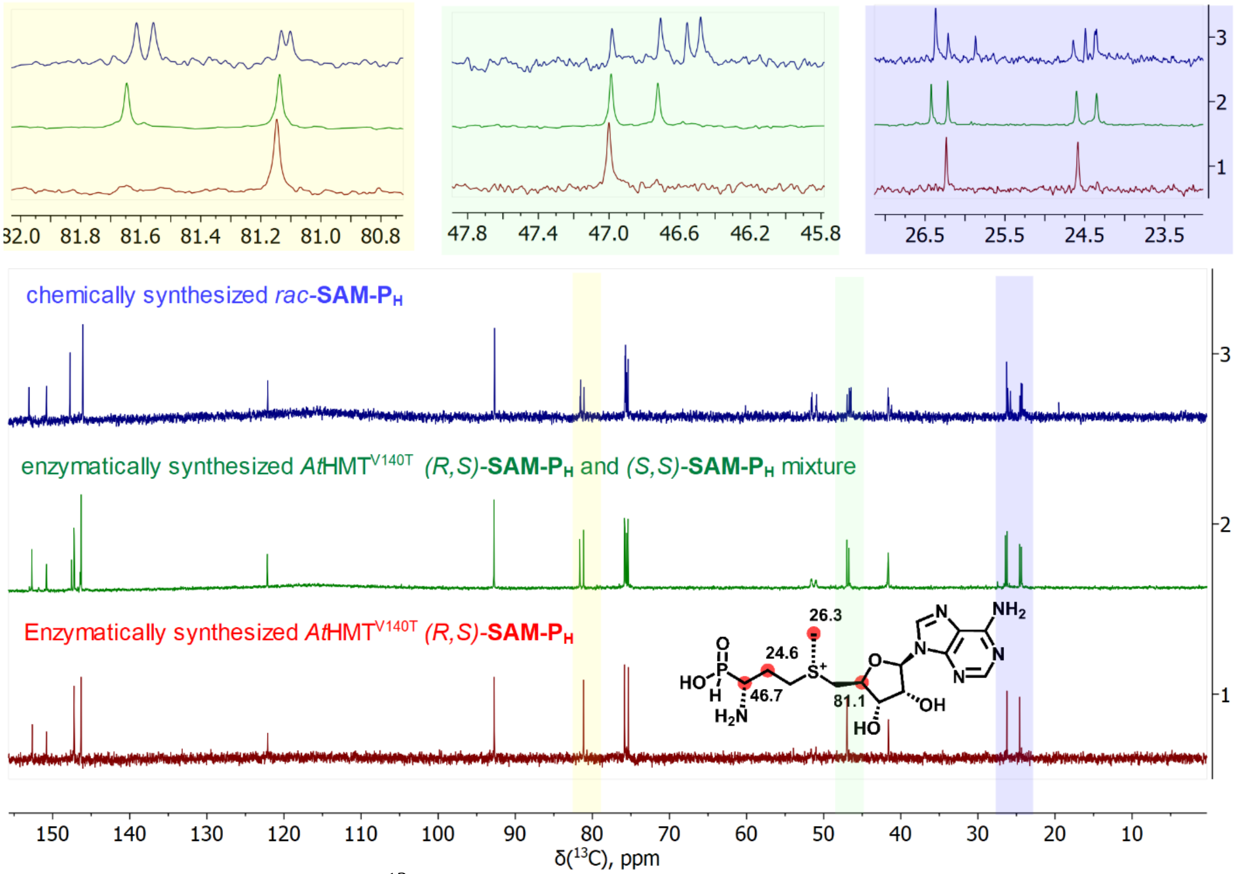

Supplementary Figure S12. ^{13}C NMR spectrum of various stereoisomers of the **SAM-P$_H$**.

3.6 NMR study of COMT catalyzed methylation of protocatechuic aldehyde by phosphorus-containing mimetics of SAM

Reaction mixtures (550 µL) contained protocatechuic aldehyde (3 mM), SAM, *rac*-**SAM-P$_H$**, *(R,S)*-**SAM-P$_H$** or *rac*-**SAM-P$_5$** (3-12 mM), COMT (20 µM), $MgCl_2$ (18 mM), chloride-free potassium phosphate buffer (50 mM, pH 7.5) and deuterated water (10 vol %) for the frequency lock.
The reaction mixtures were incubated at 37°C in the NMR spectrometer and the ^{1}H NMR spectra were recorded every 2 min during the first 4 h. The noesypr1d pulseprogram was used for water suppression. The synthesis of the protocatechuic aldehyde and other products was monitored by the appearance of its signals on NMR spectra (Supplementary Figure S13). The concentration of product was measured by integration of NMR signals relative to the internal standard DSS-d$_6$. The assays were repeated twice, the average values are represented on Supplementary Figure S14.

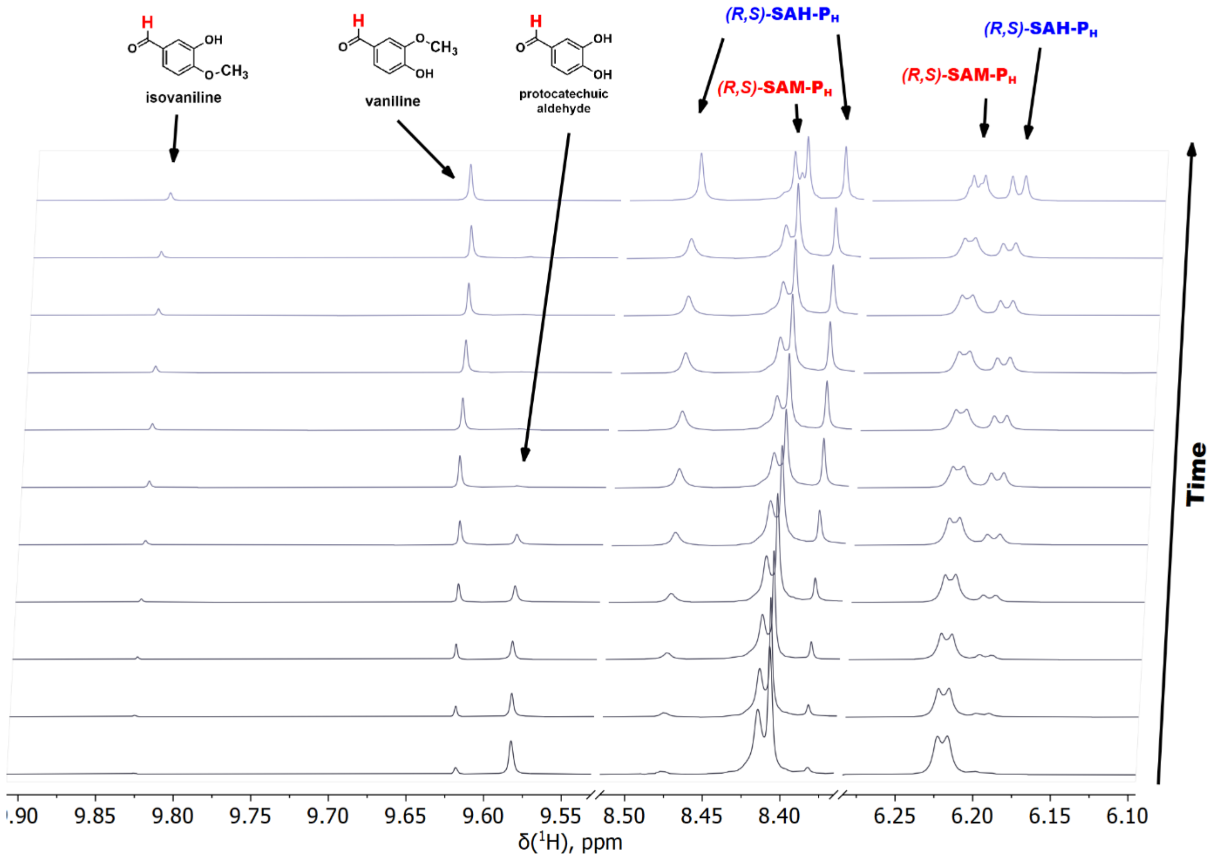

Supplementary Figure S13. A typical set of ^{1}H NMR spectra of enzymatic methylation catalysed by methyltransferase COMT using *(R,S)*-**SAM-P$_H$** as a cofactor.

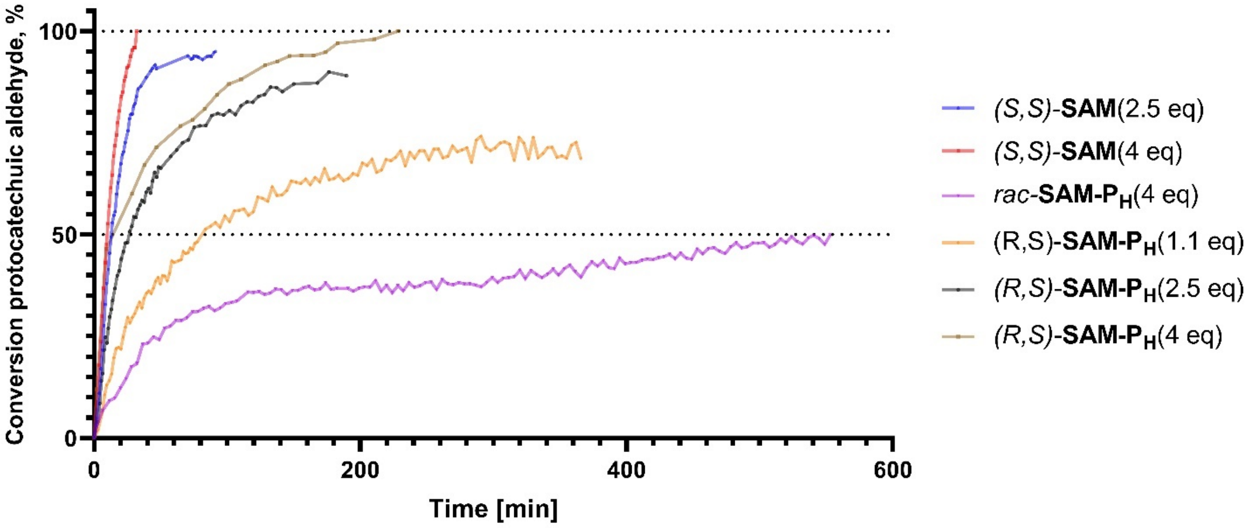

Supplementary Figure S14. COMT catalyzed methylation of protocatechuic aldehyde using different concentrations of SAM and SAM-P_H. Reaction conditions: 3 mM protocatechuic aldehyde, 20 µM COMT, 18 mM $MgCl_2$, 50 mM potassium phosphate, pH 7.5, 37°C.

3.7 NMR analysis of protein-ligand interactions

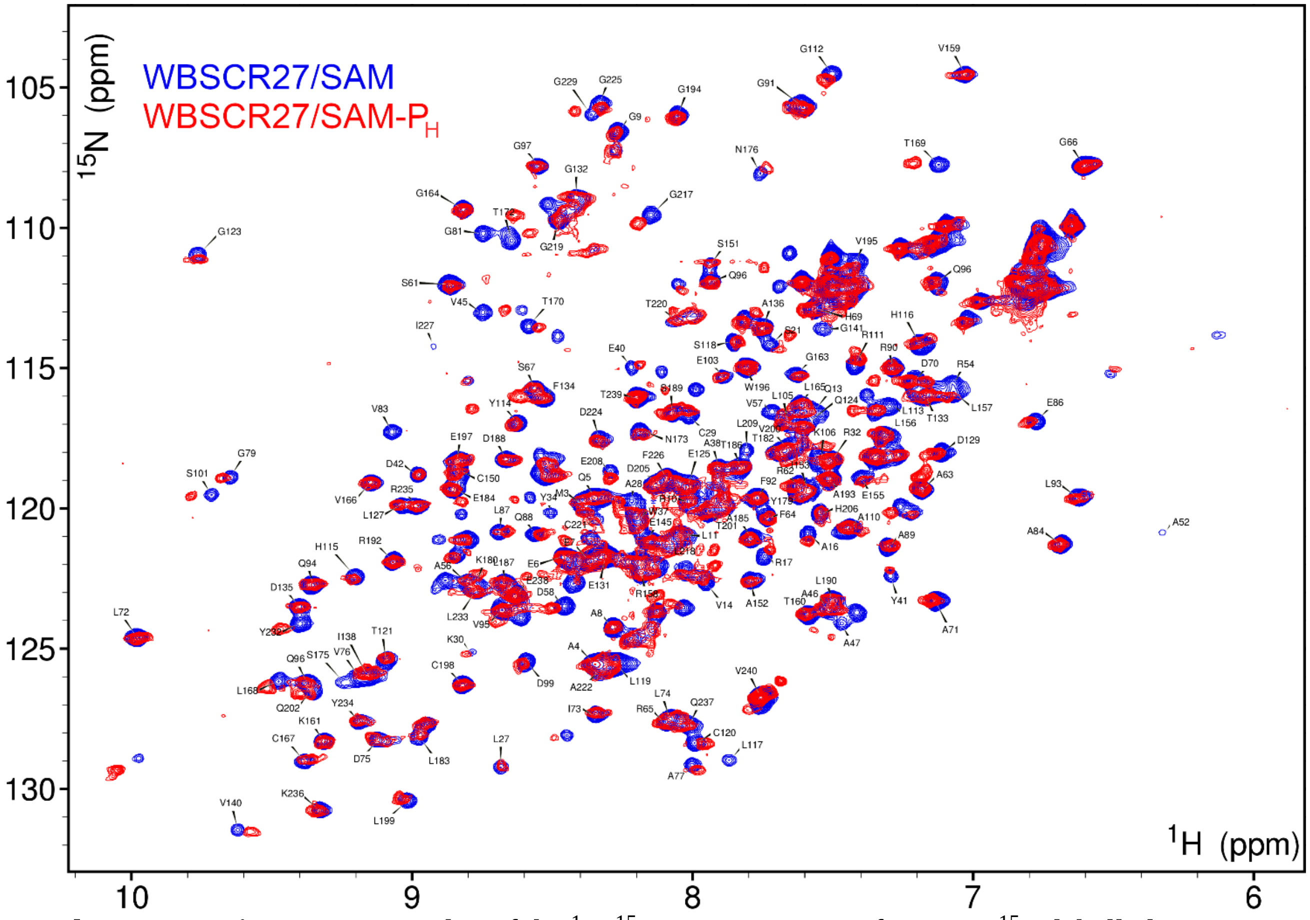

Supplementary Figure S15. Overlay of the ^{1}H-^{15}N HSQC spectra of 0.4 mM ^{15}N-labelled WBSCR27 in the presence of 5 mM SAM (blue) and 5 mM **SAM-P$_H$** (red). Labels indicate the amino acid residues.

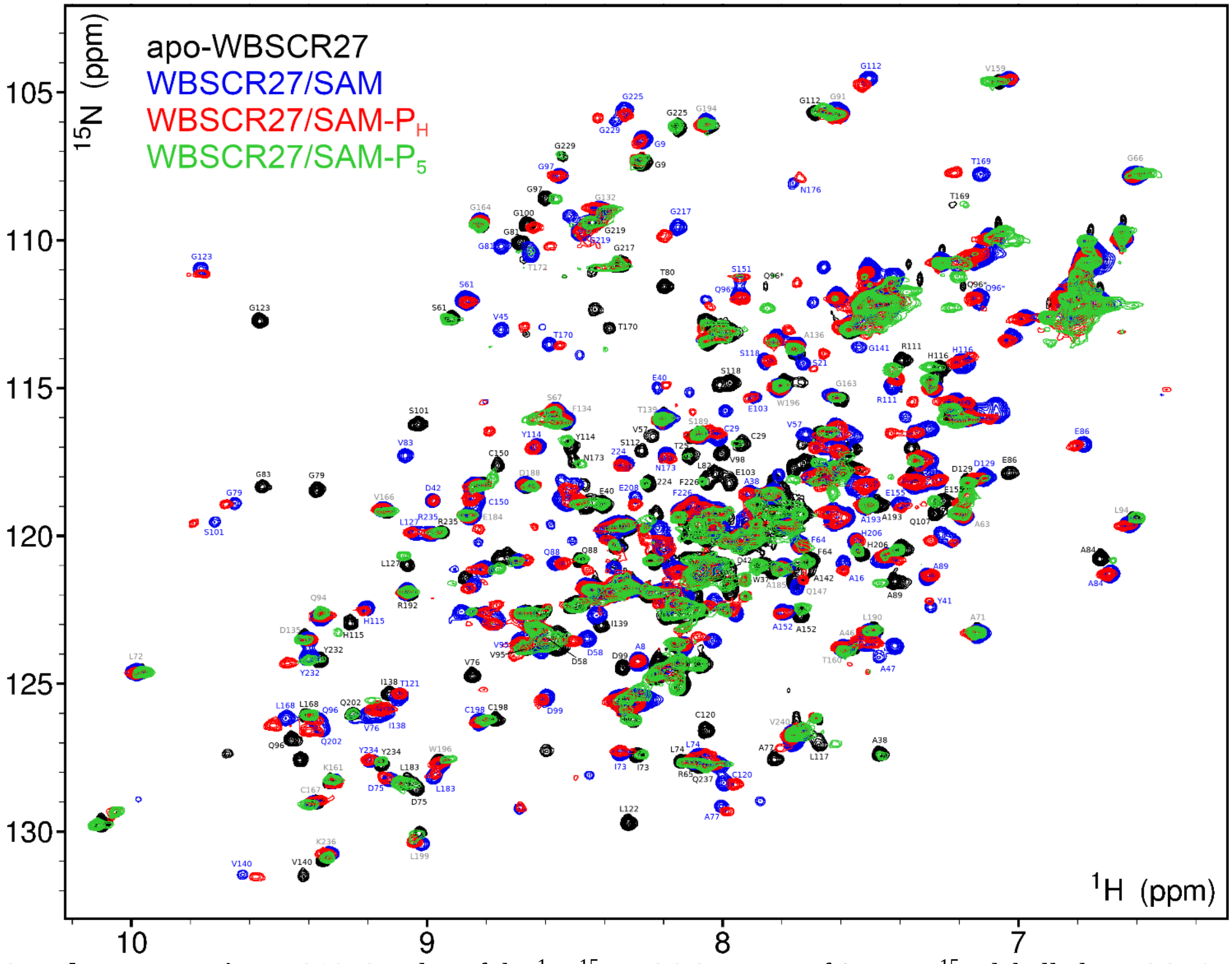

Supplementary Figure S16. Overlay of the ^{1}H-^{15}N HSQC spectra of 0.4 mM ^{15}N-labelled WBSCR27 in the apo state (black), in the presence of 5 mM SAM (blue), 5 mM **SAM-P$_H$**, (red) and 5 mM **SAM-P$_5$** (green). Labels indicate the amino acid residues: black labels for apo-WBSCR27, blue labels for WBSCR27 in presence of SAM, and grey labels – for those peaks which does not change the position by the ligand binding.

3.8 The MTAN catalyzed degradation of organophosphorus mimetics of SAH studied by HPLC

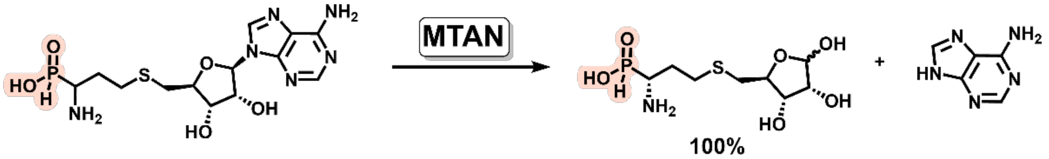

rac-**SAH-P$_H$**

Supplementary Figure S17. The MTAN catalysed irreversible cleavage of the glycoside bond of **SAH-P$_H$**.

Reaction mixtures (200 µL) contained SAH, or *rac*-**SAH-P$_H$**, or *rac*-**SAH-P$_5$** (1 mM), MTAN (0.1 µM) and chloride-free potassium phosphate buffer (50 mM, pH 7.5). Immediately after adding MTAN, the reaction mixtures were briefly mixed by pipetting and then after a certain time period (0 sec, 90 sec, 10 min, 20 min) 20 µL of reaction mixture were taken and quenched by adding to 60 µL of acetonitrile. 30 µL of obtained solutions were transferred to HPLC sample vial inserts, mixed with 970 uL of mQ water and used for HPLC analysis.

Analyses were performed on a Waters Breeze 2 HPLC System (Waters 1525 Binary HPLC Pump Waters 2489 UV/Visible Detector, Waters 2707 Autosampler) with a Kromasil Eternity C18 (4.6 x 250 mm column, 5 µm particle size) reversed-phase column (AkzoNobel, Ireland). For separation of SAH analog and adenine, the mobile phase was the mixture of 0.1% formic acid – acetonitrile (98:2, v/v). The flow rate was 1 mL/min, injection volume 20 µL, and column thermostat temperature was 30°C. **SAH-P$_H$** and adenine were detected at 260 nm and quantified using the internal normalization method (Supplementary Figures S18-S20). All reactions were carried out in triplicate and independently analysed.

NMR measurements gave a similar result to HPLC. SAM, *rac*-**SAM-P$_H$**, and *rac*-**SAM-P$_5$** were not cleaved by MTAN.

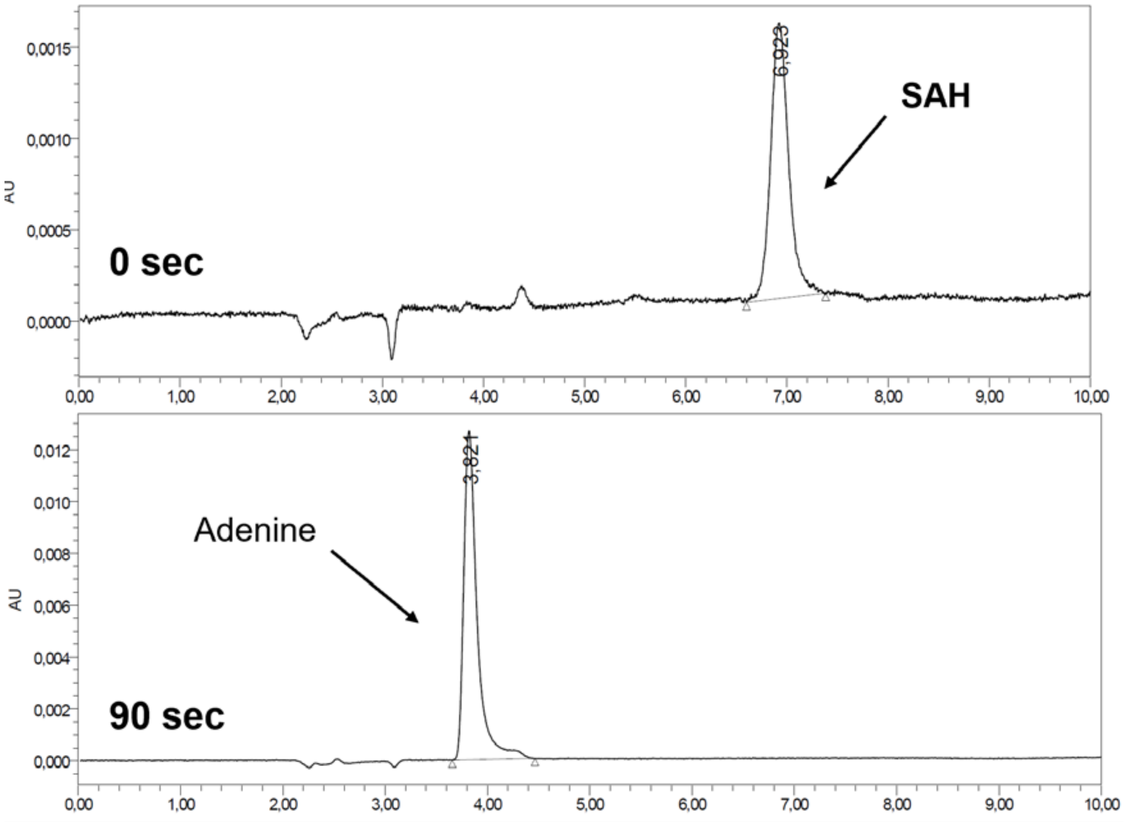

Supplementary Figure S18. HPLC chromatogram of MTAN-catalyzed SAH degradation. The retention times: SAH - 6.9 min, adenine - 3.8 min.

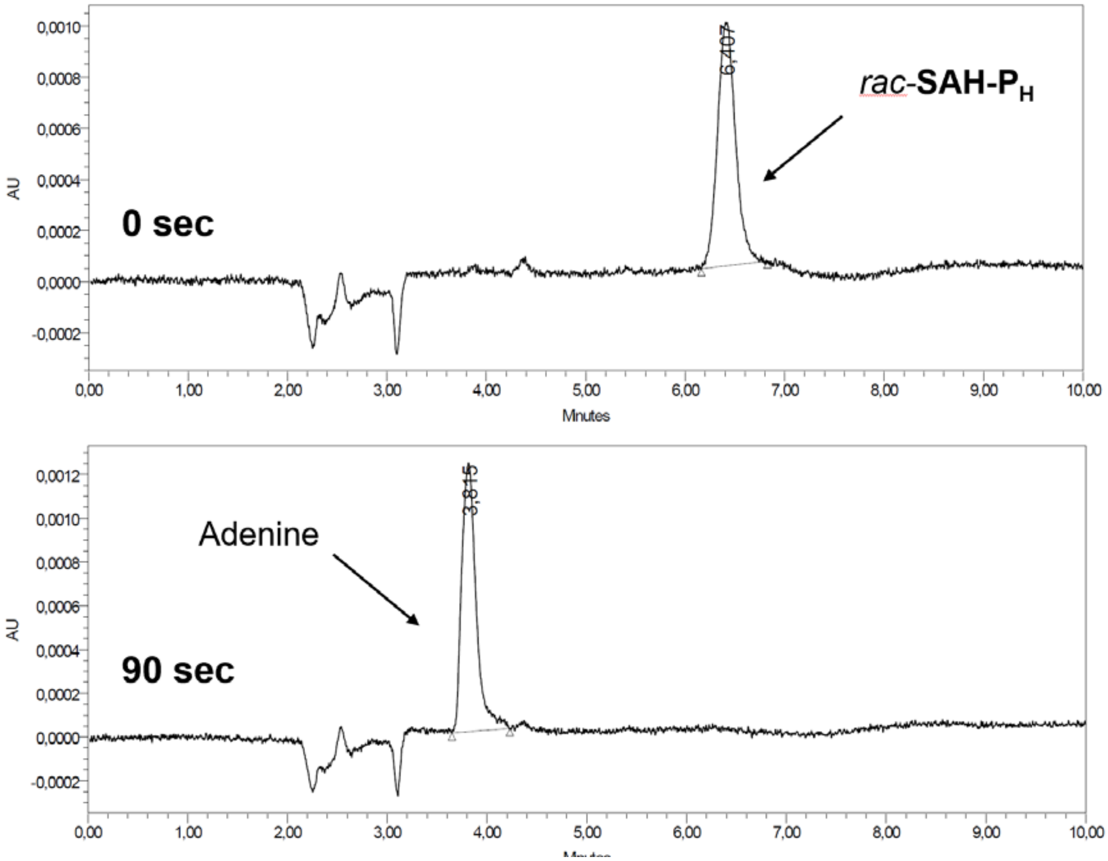

Supplementary Figure S19. HPLC chromatogram of MTAN-catalyzed *rac*-**SAH-P$_H$** degradation. The retention times: *rac*-**SAH-P$_H$ -** 6.4 min, adenine - 3.8 min.

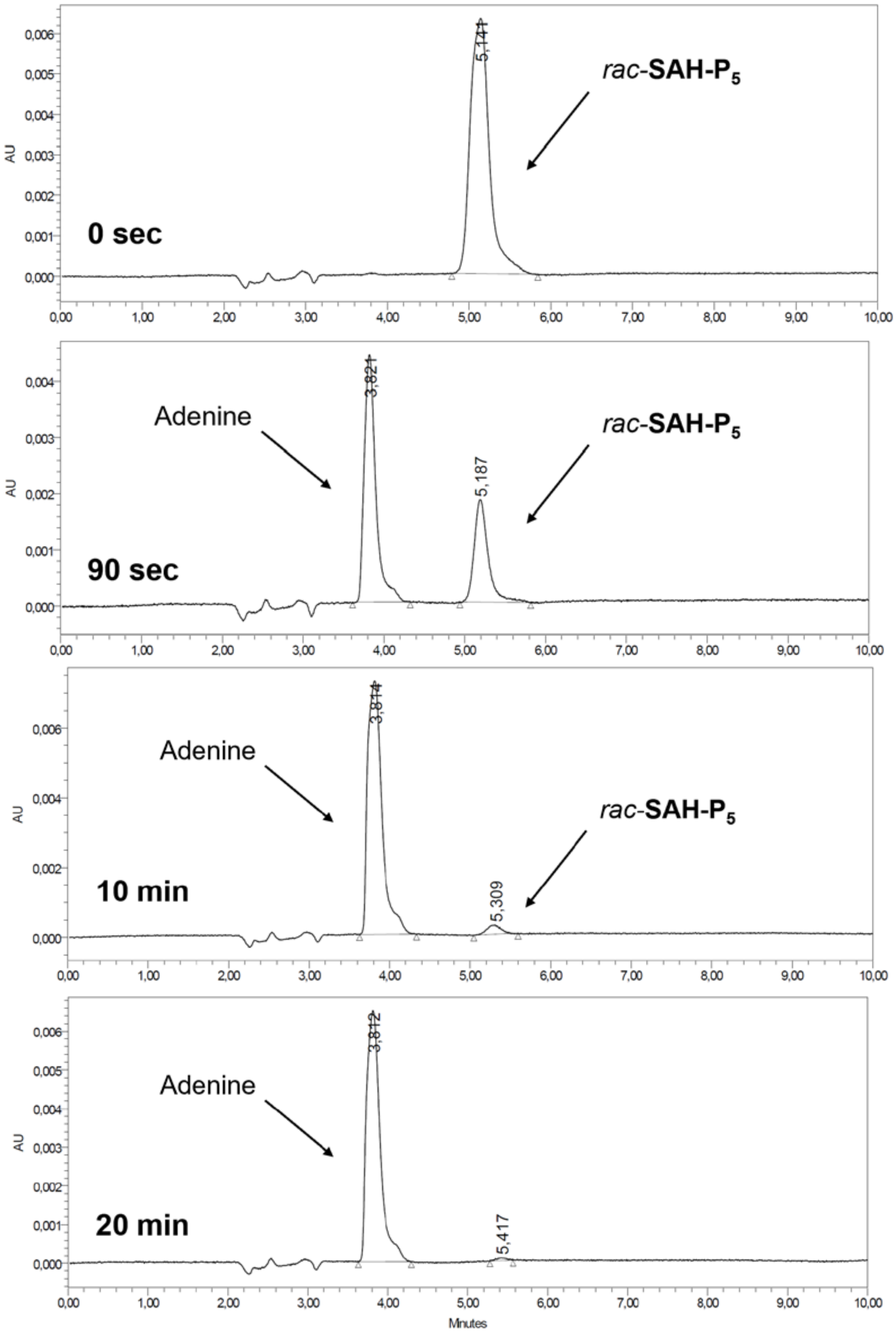

Supplementary Figure S20. HPLC chromatogram of MTAN-catalyzed *rac*-**SAH-P**$_5$ degradation. The retention times: *rac*-**SAH-P**$_5$ **-** 5.2 min, adenine - 3.8 min.

4 NMR spectra of small-molecules

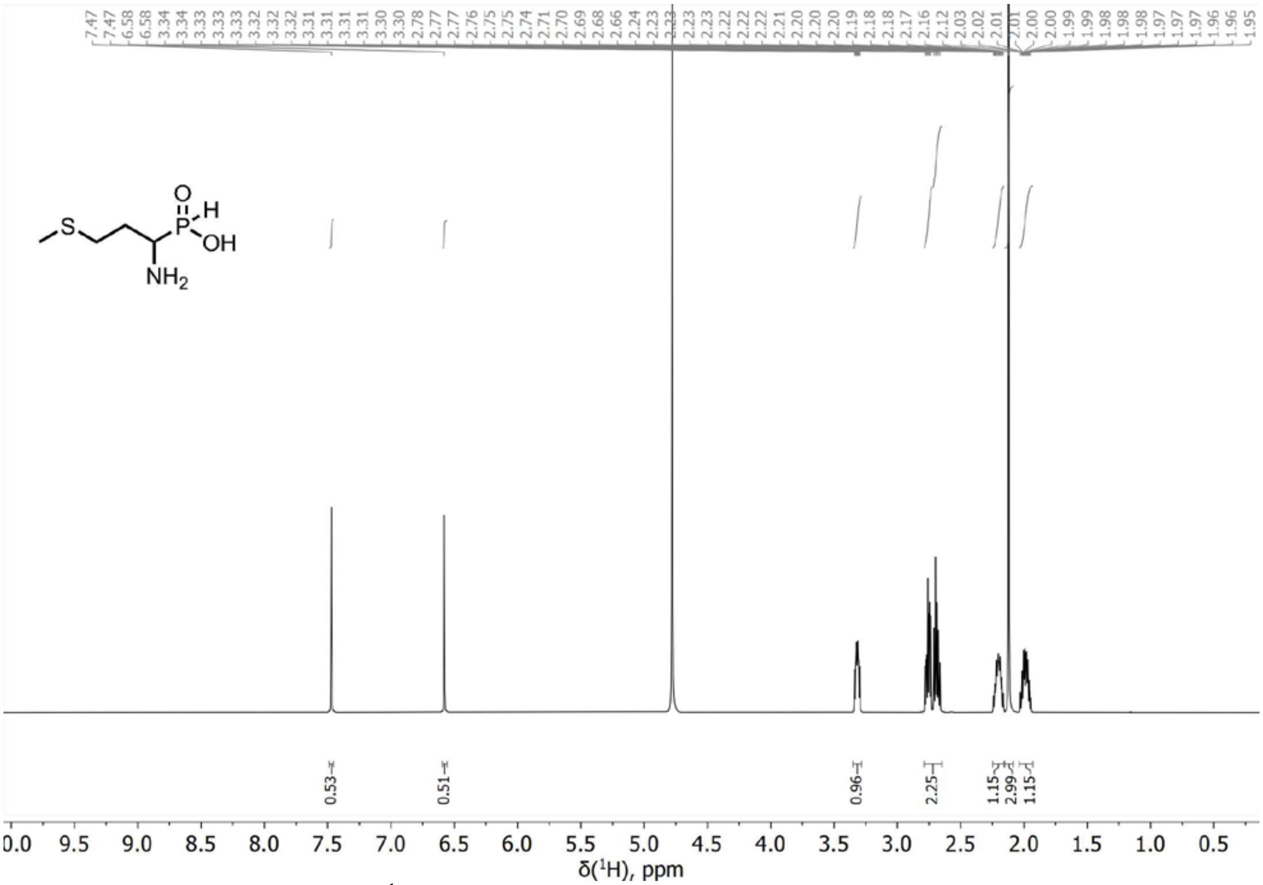

Supplementary Figure S21. ^{1}H NMR spectrum of *rac*-Met-P_H

^{1}H NMR (600 MHz, D_2O) δ = 7.0 (d, $^{1}J_{PH}$=534.8, 1H), 3.4 – 3.3 (m, 1H), 2.8 – 2.6 (m, 2H), 2.3 – 2.1 (m, 1H), 2.1 (s, 3H), 2.0 – 1.9 (m, 1H).

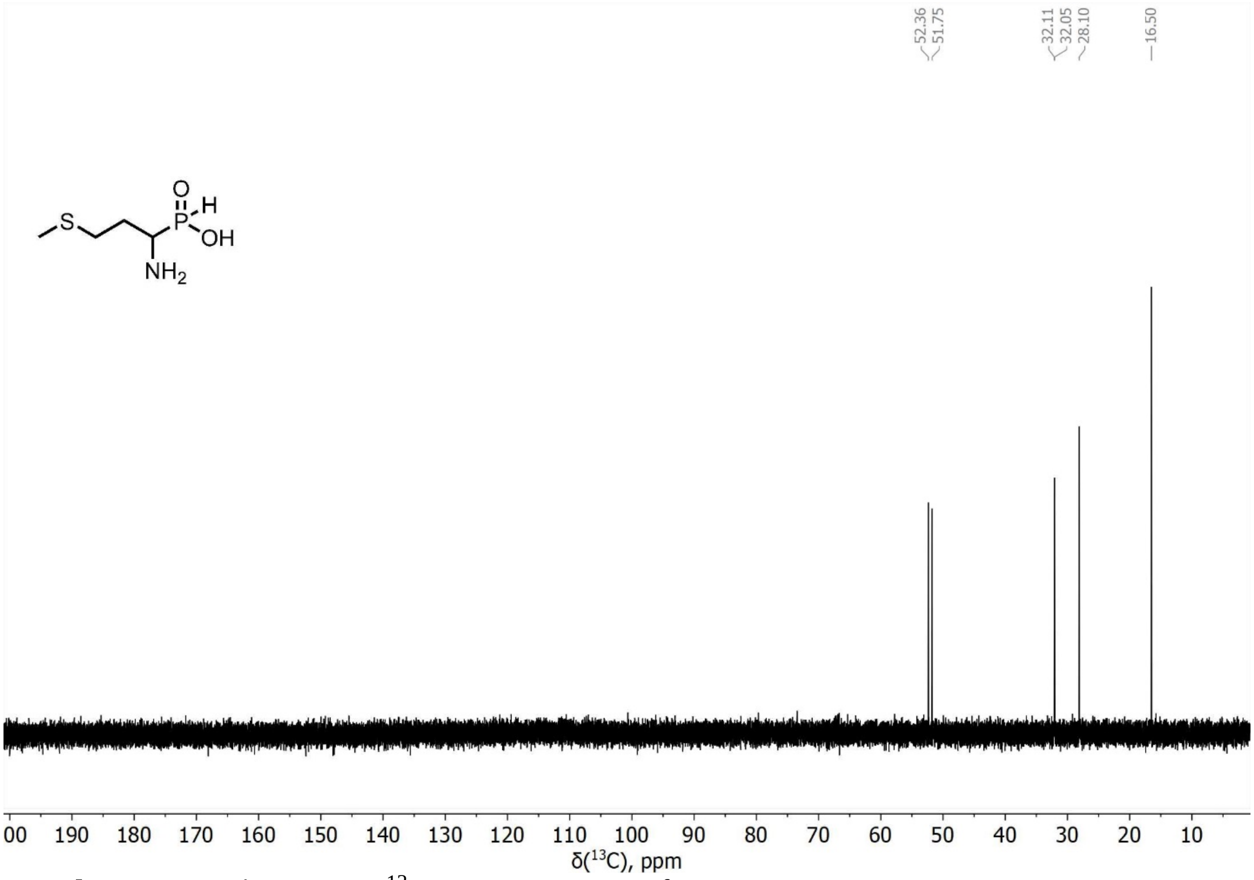

Supplementary Figure S22. ^{13}C NMR spectrum of *rac*-Met-P_H

^{13}C NMR (151 MHz, D_2O) δ = 52.1 (d, *J*=91.5), 32.1 (d, *J*=9.7), 28.1, 16.5

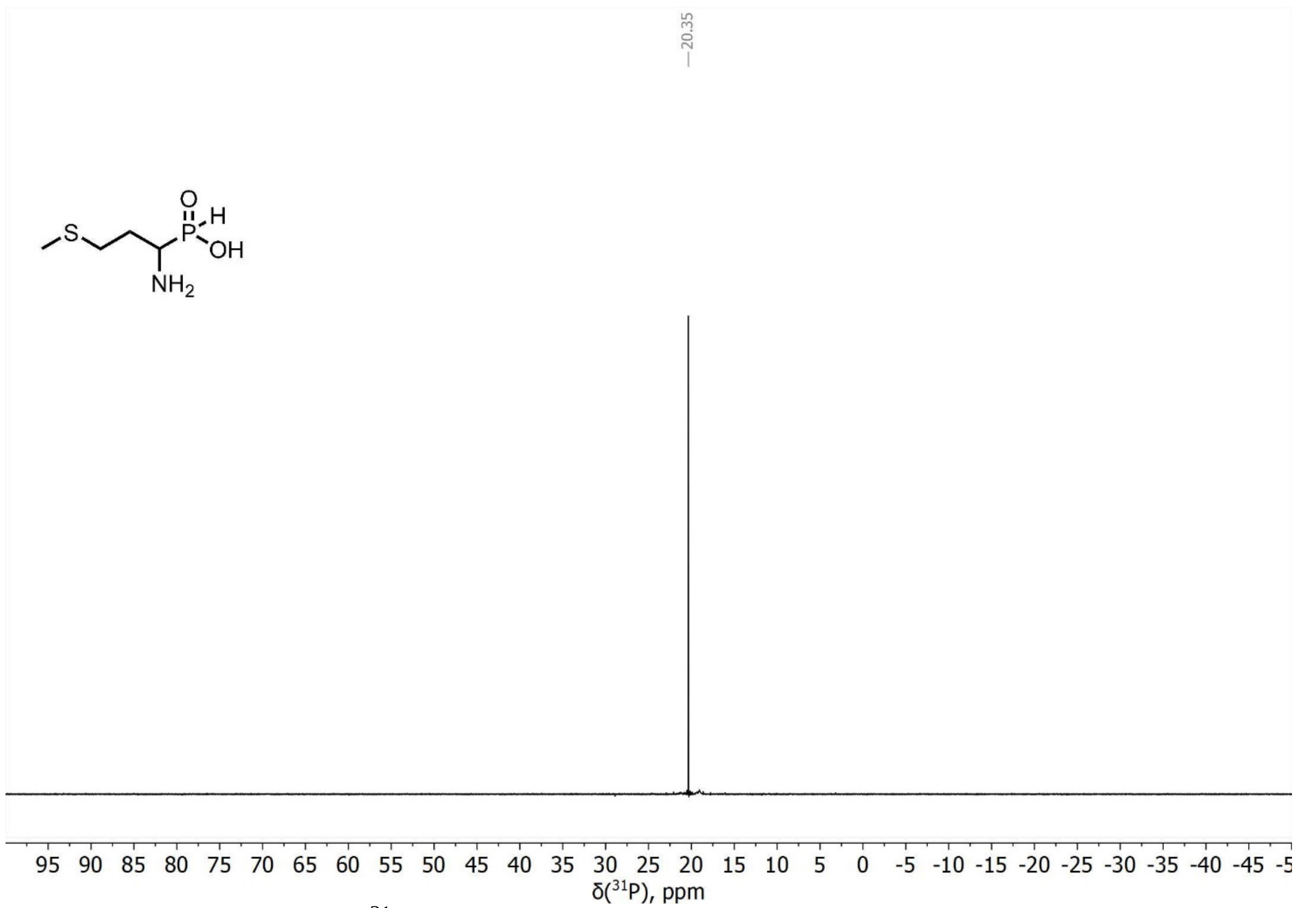

Supplementary Figure S23. ^{31}P NMR spectrum of *rac*-Met-P_H

^{31}P NMR (243 MHz, D_2O) δ = 20.3.

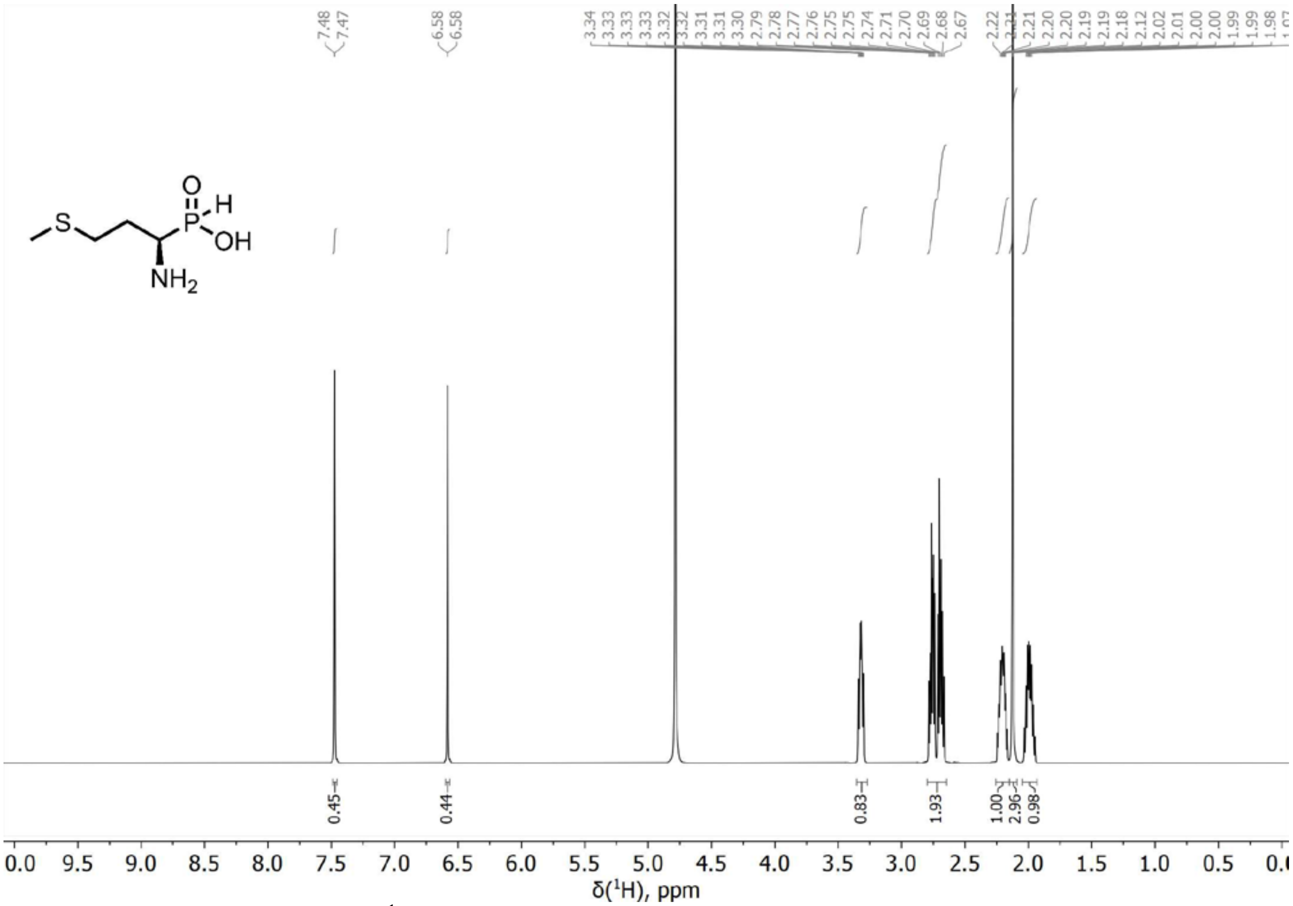

Supplementary Figure S24. ^{1}H NMR spectrum of (*R)*-Met-P_H

^{1}H NMR (600 MHz, D_2O) δ = 7.0 (d, $^{1}J_{PH}$=534.8, 1H), 3.4 – 3.3 (m, 1H), 2.8 – 2.6 (m, 2H), 2.3 – 2.1 (m, 1H), 2.1 (s, 3H), 2.0 – 1.9 (m, 1H).

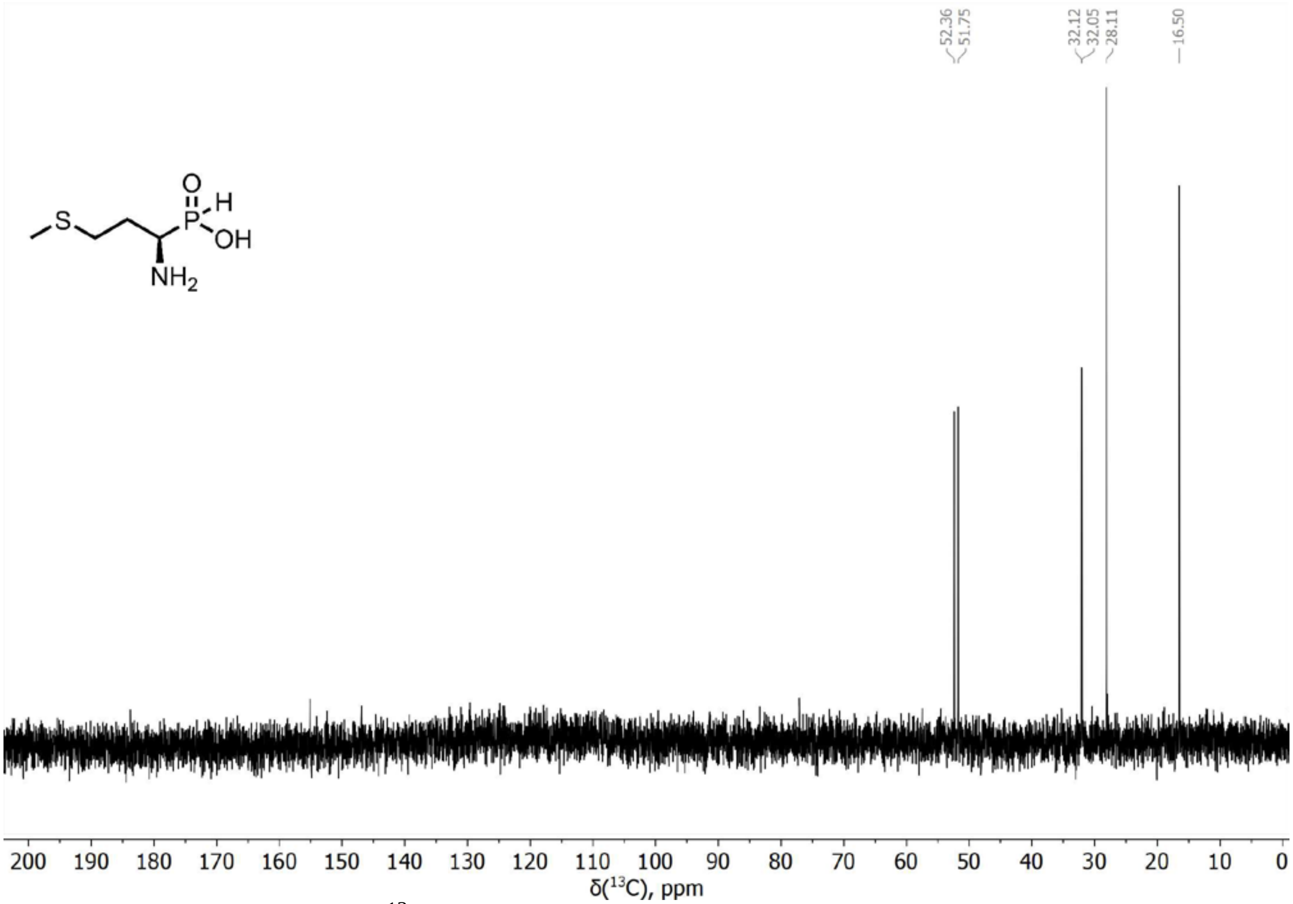

Supplementary Figure S25. ^{13}C NMR spectrum of (*R*)-Met-P_H

^{13}C NMR (151 MHz, D_2O) δ = 52.1 (d, *J*=91.5), 32.1 (d, *J*=9.7), 28.1, 16.5.

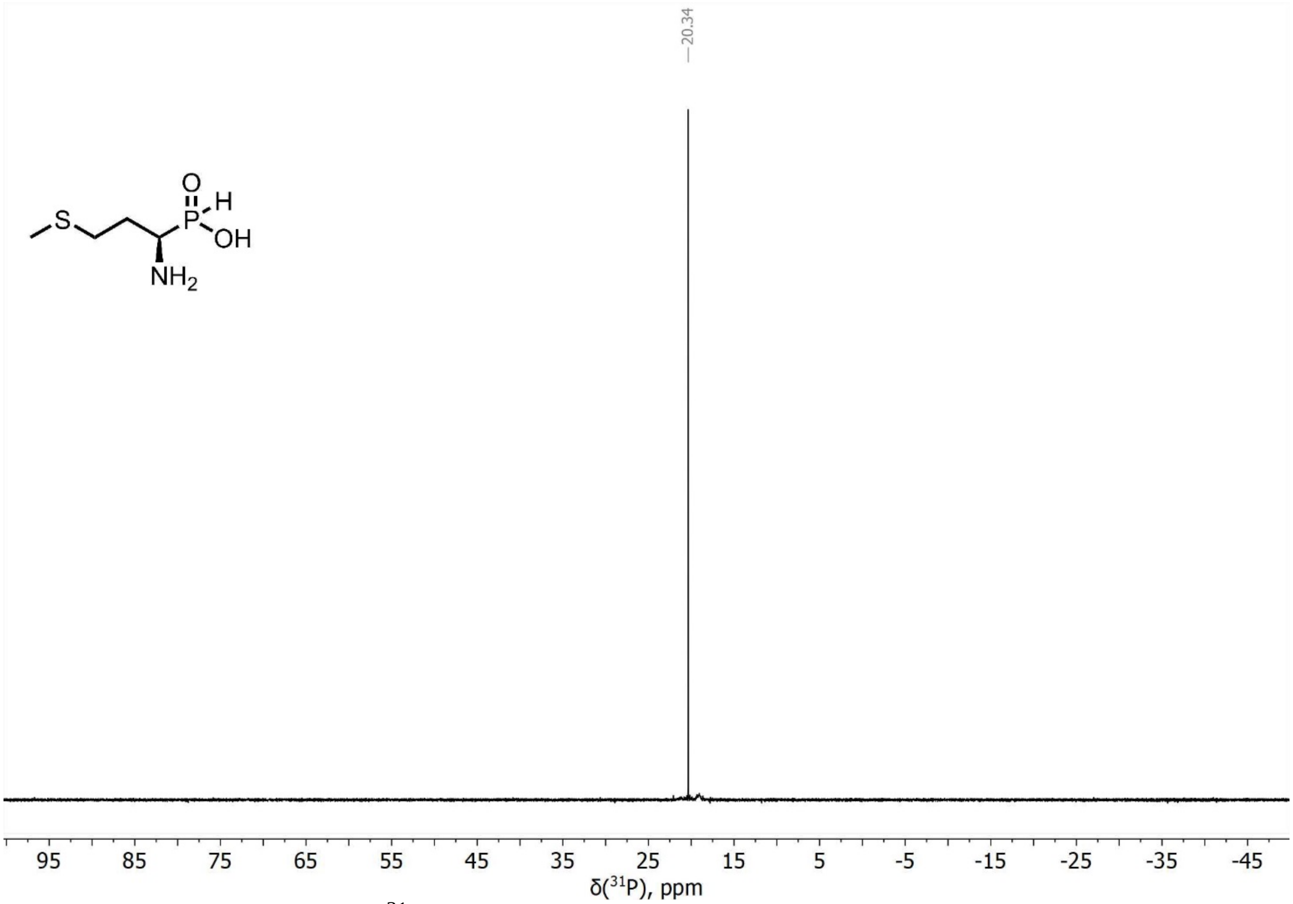

Supplementary Figure S26. ^{31}P NMR spectrum of (*R)*-Met-P_H

^{31}P NMR (243 MHz, D_2O) δ = 20.3.

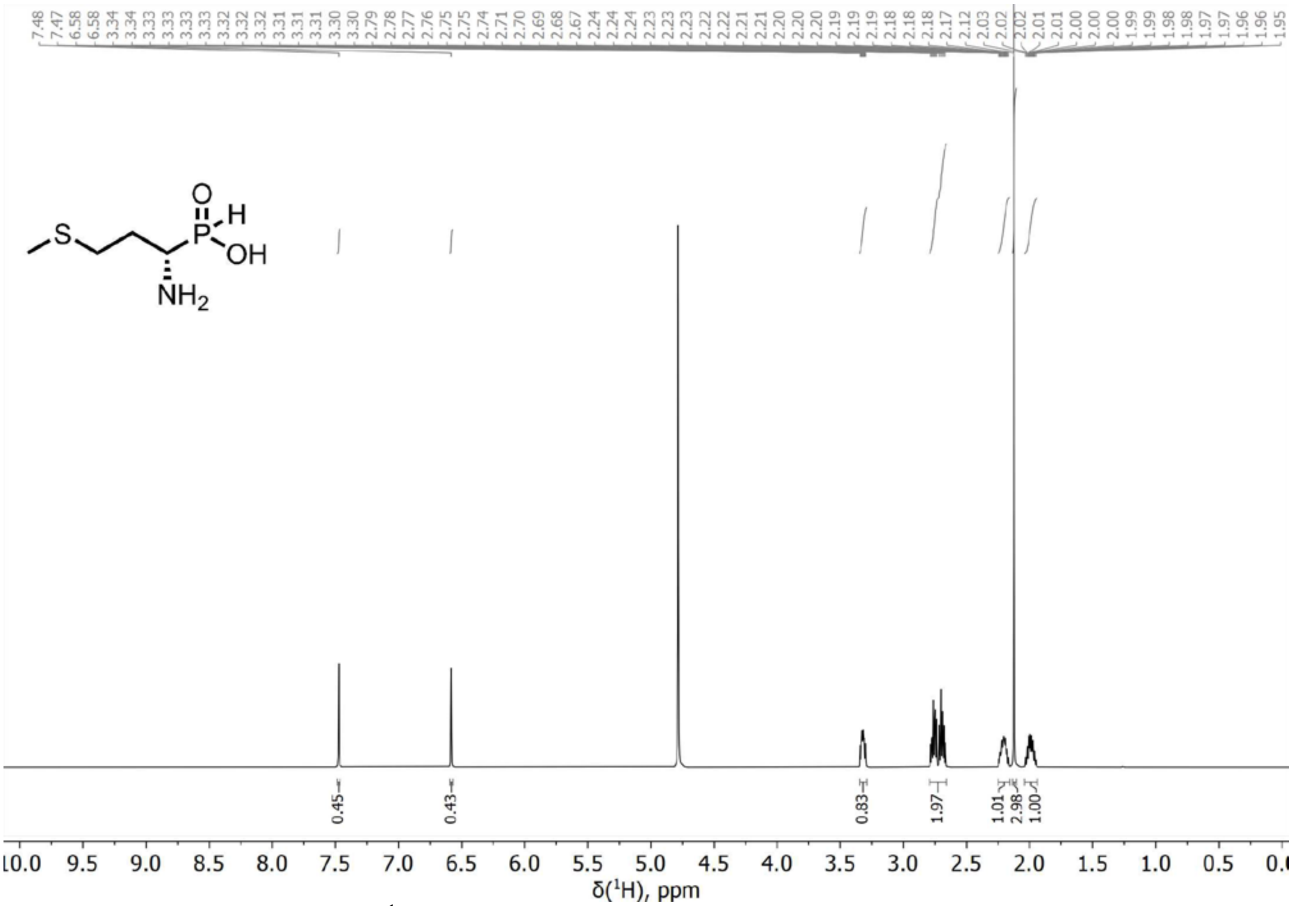

Supplementary Figure S27. ^{1}H NMR spectrum of (*S*)-Met-P_H

^{1}H NMR (600 MHz, D_2O) δ = 7.0 (d, $^1J_{PH}$=534.8, 1H), 3.4 – 3.3 (m, 1H), 2.8 – 2.6 (m, 2H), 2.3 – 2.1 (m, 1H), 2.1 (s, 3H), 2.0 – 1.9 (m, 1H).

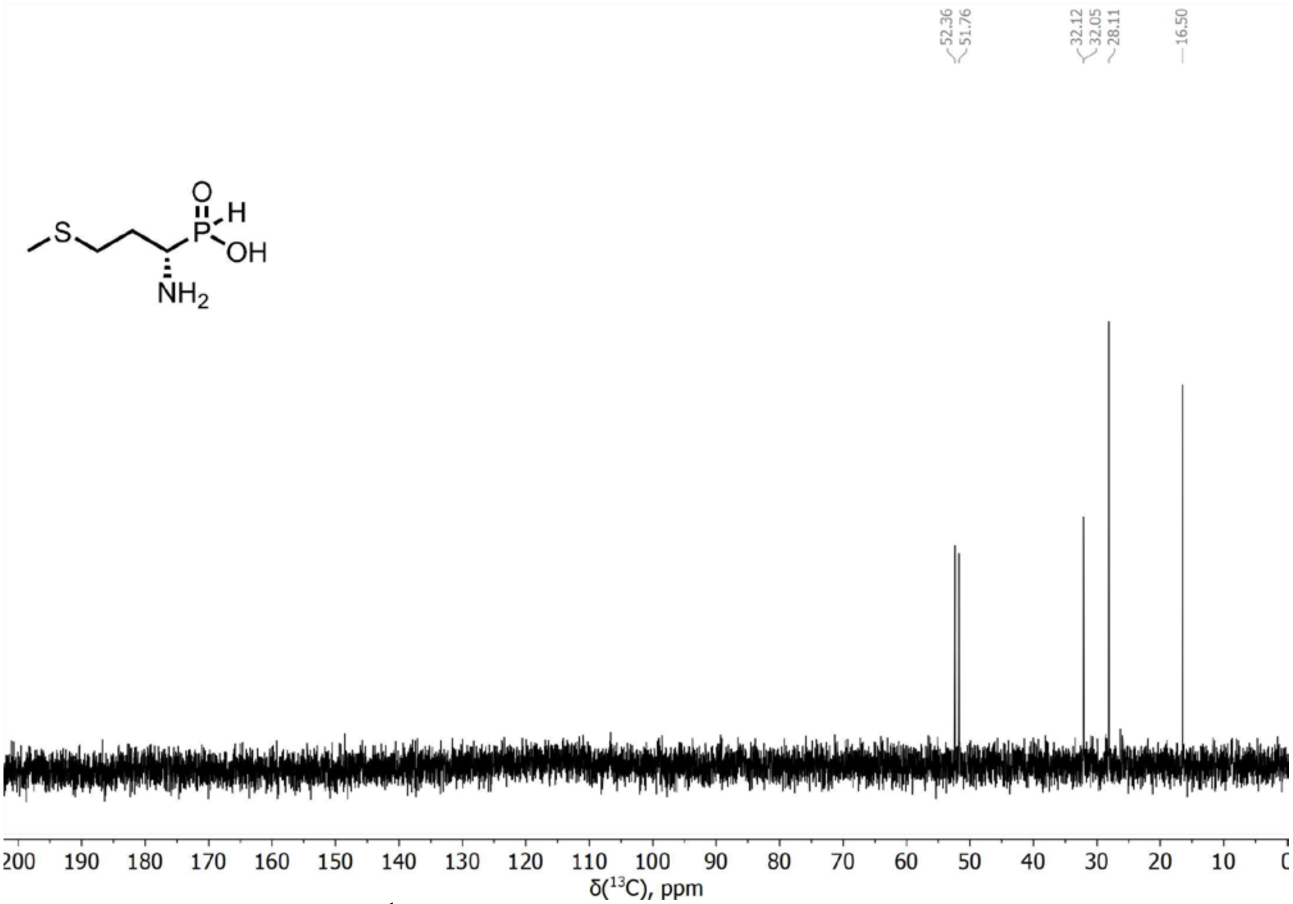

Supplementary Figure S28. ^{1}H NMR spectrum of (*S*)-Met-P_H

^{13}C NMR (151 MHz, D_2O) δ = 52.1 (d, *J*=91.5), 32.1 (d, *J*=9.7), 28.1, 16.5.

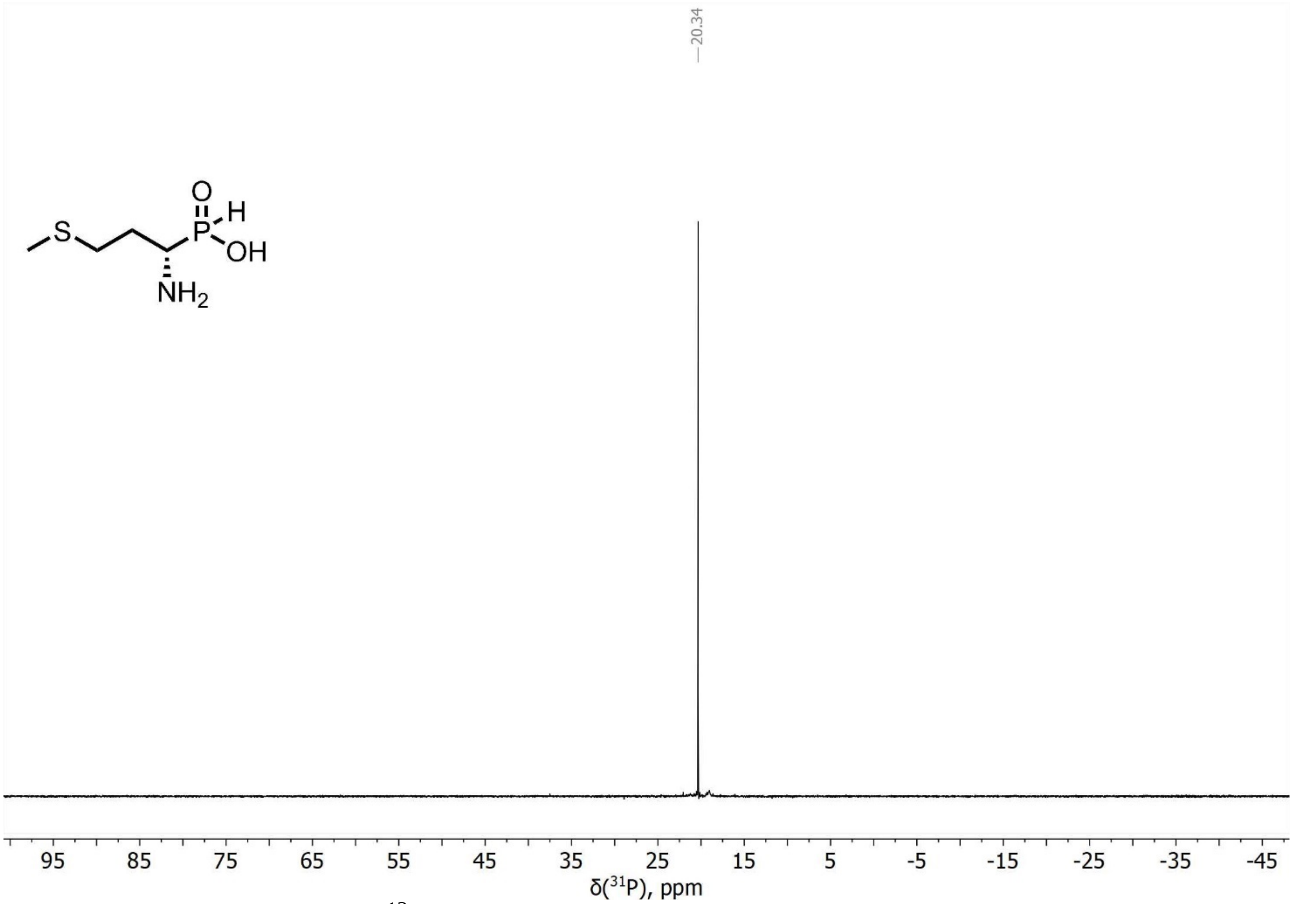

Supplementary Figure S29. ^{13}C NMR spectrum of (*S)*-Met-P_H

^{31}P NMR (243 MHz, D_2O) δ = 20.3.

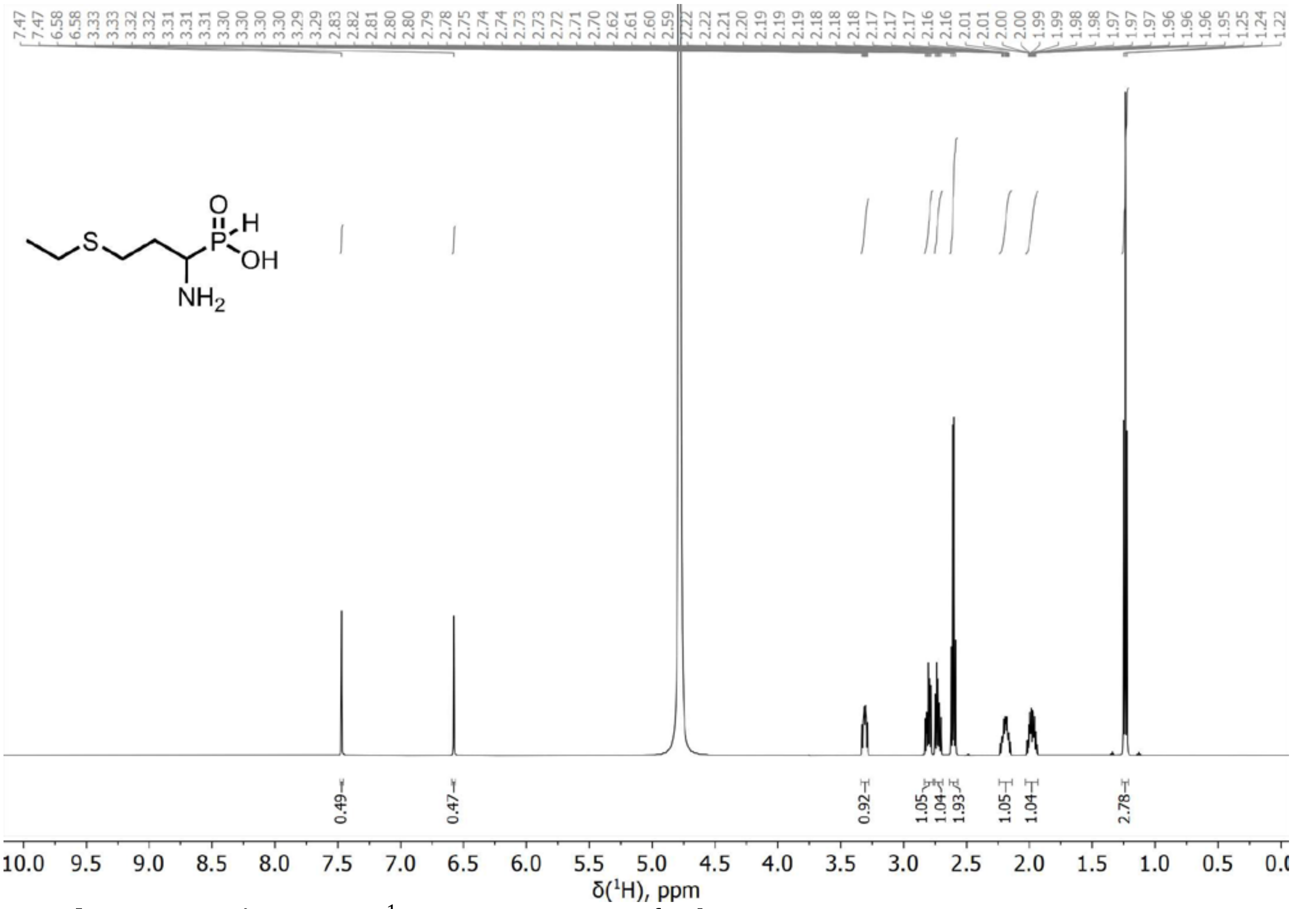

Supplementary Figure S30. ^{1}H NMR spectrum of Ethionine-P_H

^{1}H NMR (600 MHz, D_2O) δ = 7.0 (d, $^{1}J_{PH}$=534.5, 1H), 3.3 – 3.3 (m, 1H), 2.8 – 2.8 (m, 1H), 2.8 – 2.7 (m, 1H), 2.6 (q, *J*=7.4, 2H), 2.2 – 2.1 (m, 1H), 2.0 – 1.9 (m, 1H), 1.2 (t, *J*=7.4, 3H).

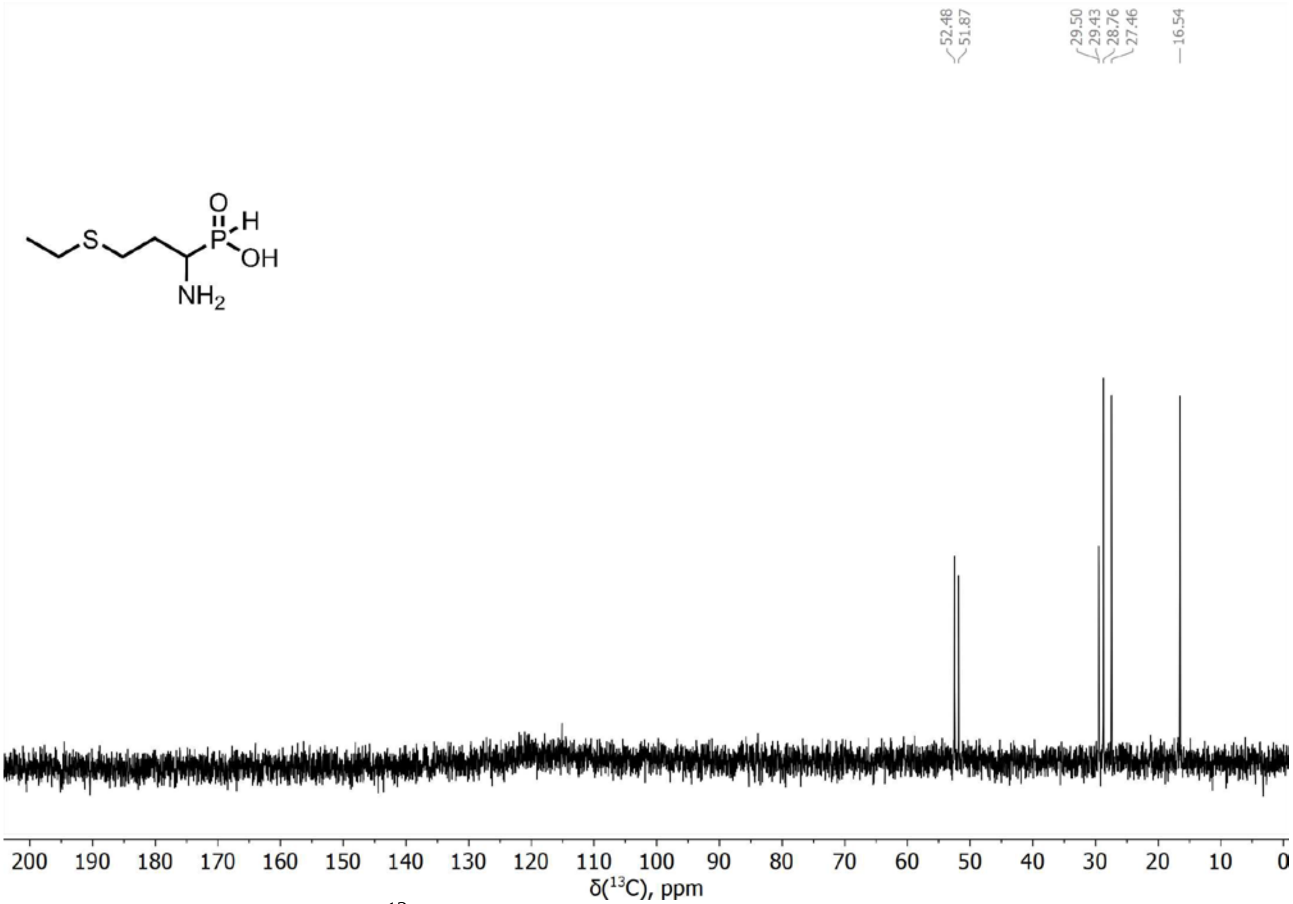

Supplementary Figure S31. ^{13}C NMR spectrum of Ethionine-P$_H$

^{13}C NMR (151 MHz, D_2O) δ = 52.2 (d, *J*=91.5), 29.5 (d, *J*=9.5), 28.8, 27.5, 16.5.

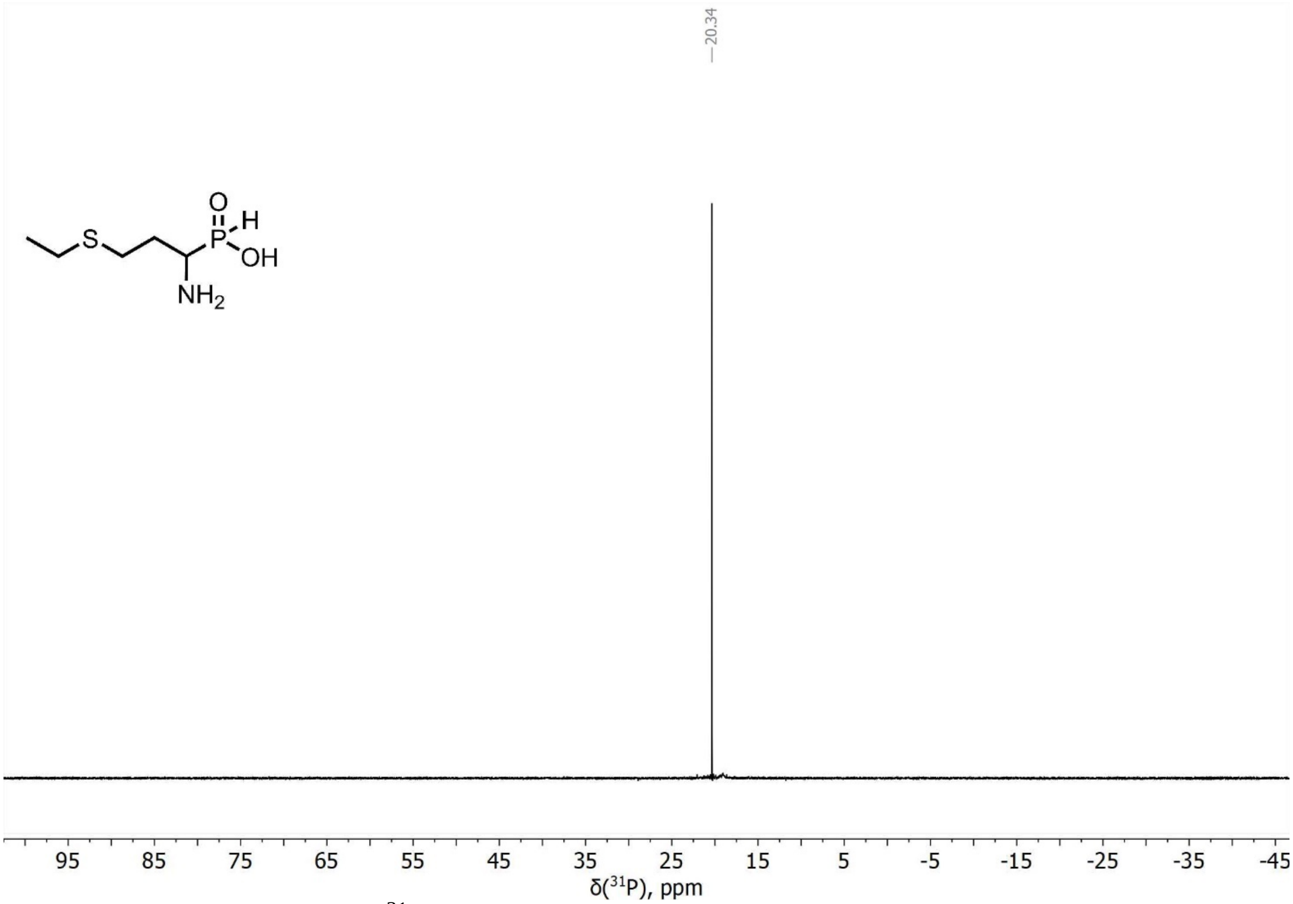

Supplementary Figure S32. ^{31}P NMR spectrum of Ethionine-P_H

^{31}P NMR (243 MHz, D_2O) δ = 20.3.

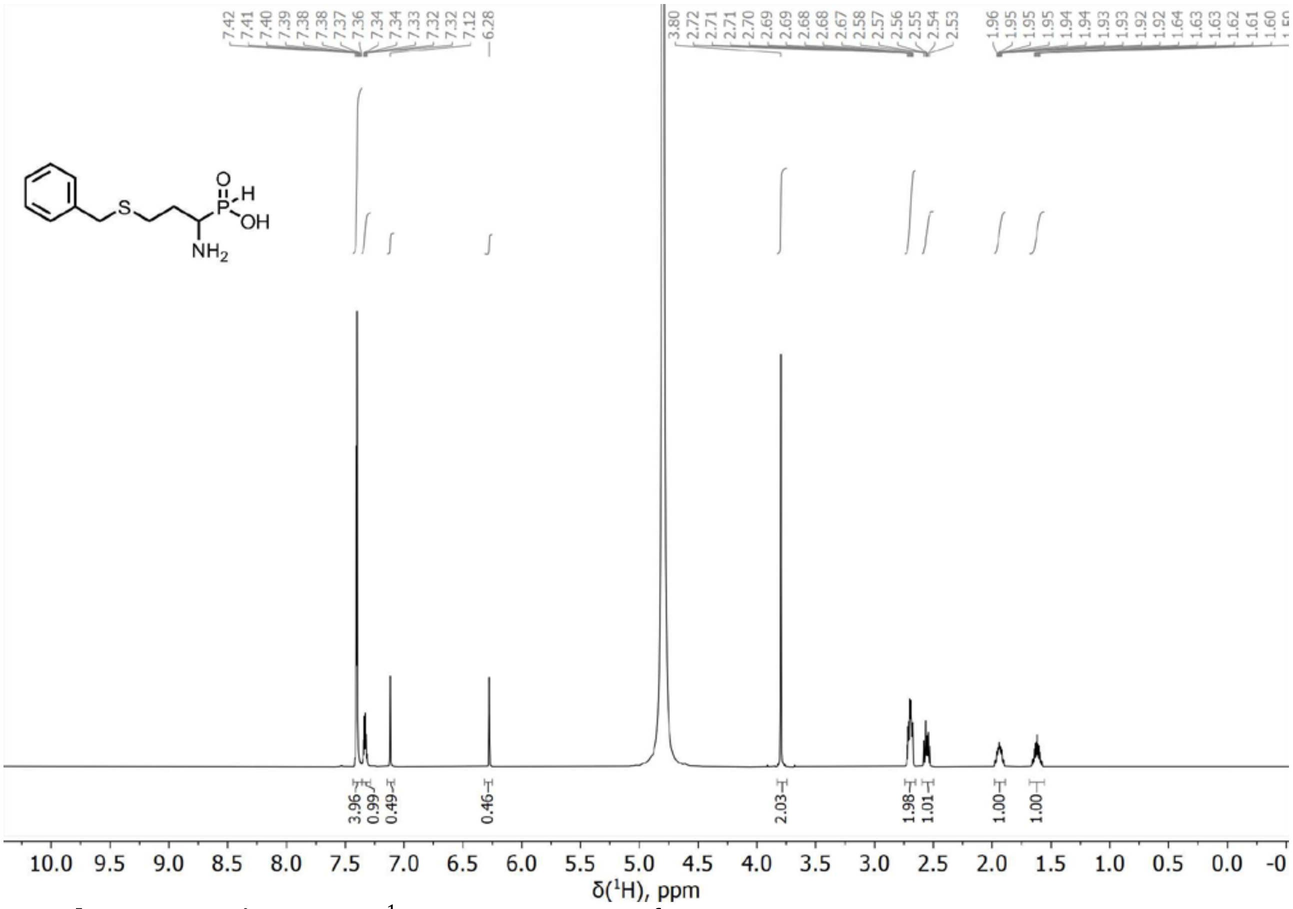

Supplementary Figure S33. ^{1}H NMR spectrum of Bn-S-Hcy-P_H

^{1}H NMR (600 MHz, D_2O) δ = 7.4 – 7.4 (m, 4H), 7.4 – 7.3 (m, 1H), 6.7 (d, $^1J_{PH}$=505.3, 1H), 3.8 (s, 2H), 2.7 – 2.7 (m, 2H), 2.6 – 2.5 (m, 1H), 2.0 – 1.9 (m, 1H), 1.7 – 1.6 (m, 1H).

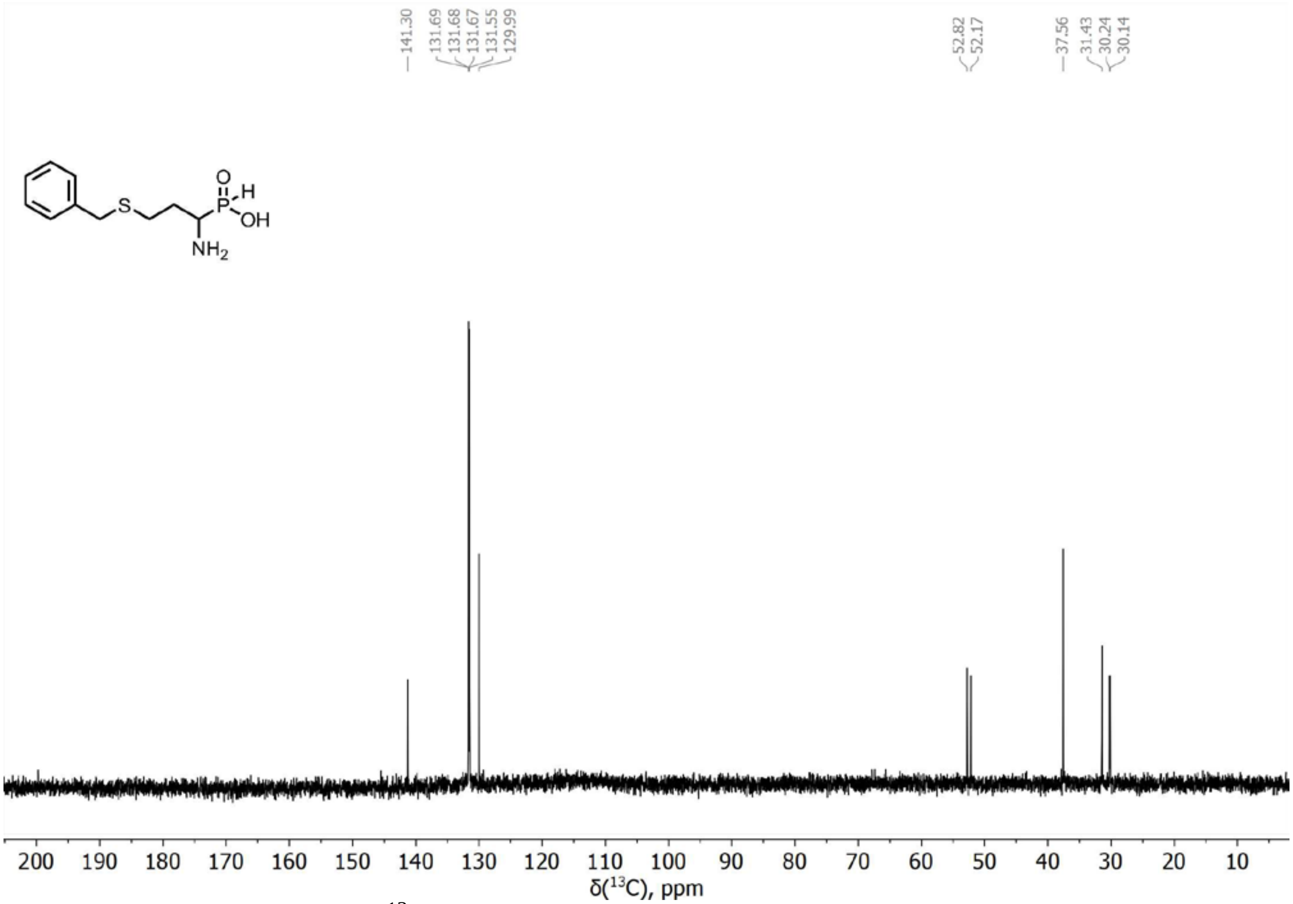

Supplementary Figure S34. ^{13}C NMR spectrum of Bn-S-Hcy-P$_H$

^{13}C NMR (151 MHz, D_2O) δ = 141.3, 131.7, 131.5, 130.0, 52.5 (d, *J*=98.8), 37.6, 31.4, 30.2 (d, *J*=14.1).

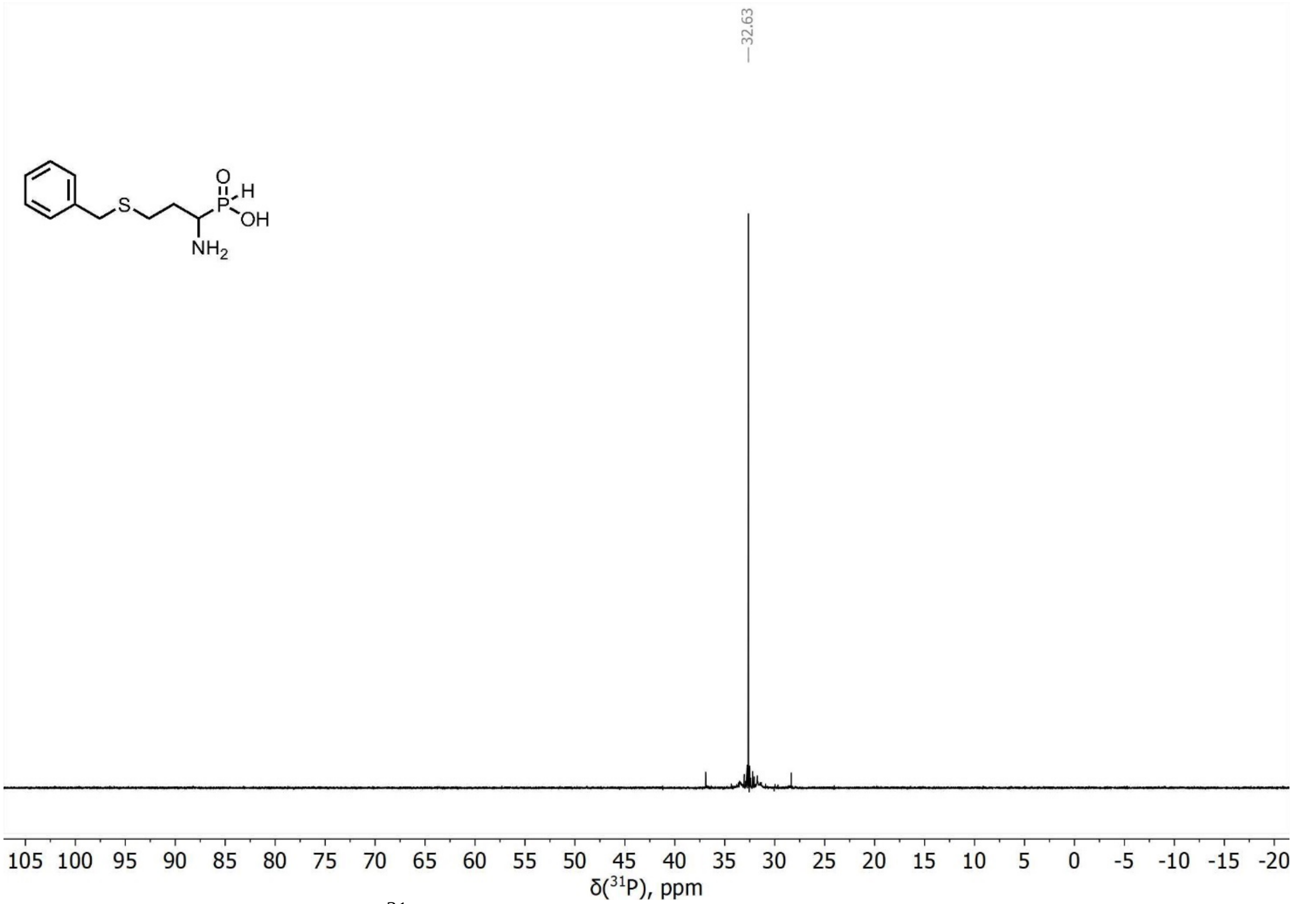

Supplementary Figure S35. ^{31}P NMR spectrum of Bn-S-Hcy-P_H

^{31}P NMR (243 MHz, D_2O) δ = 32.6.

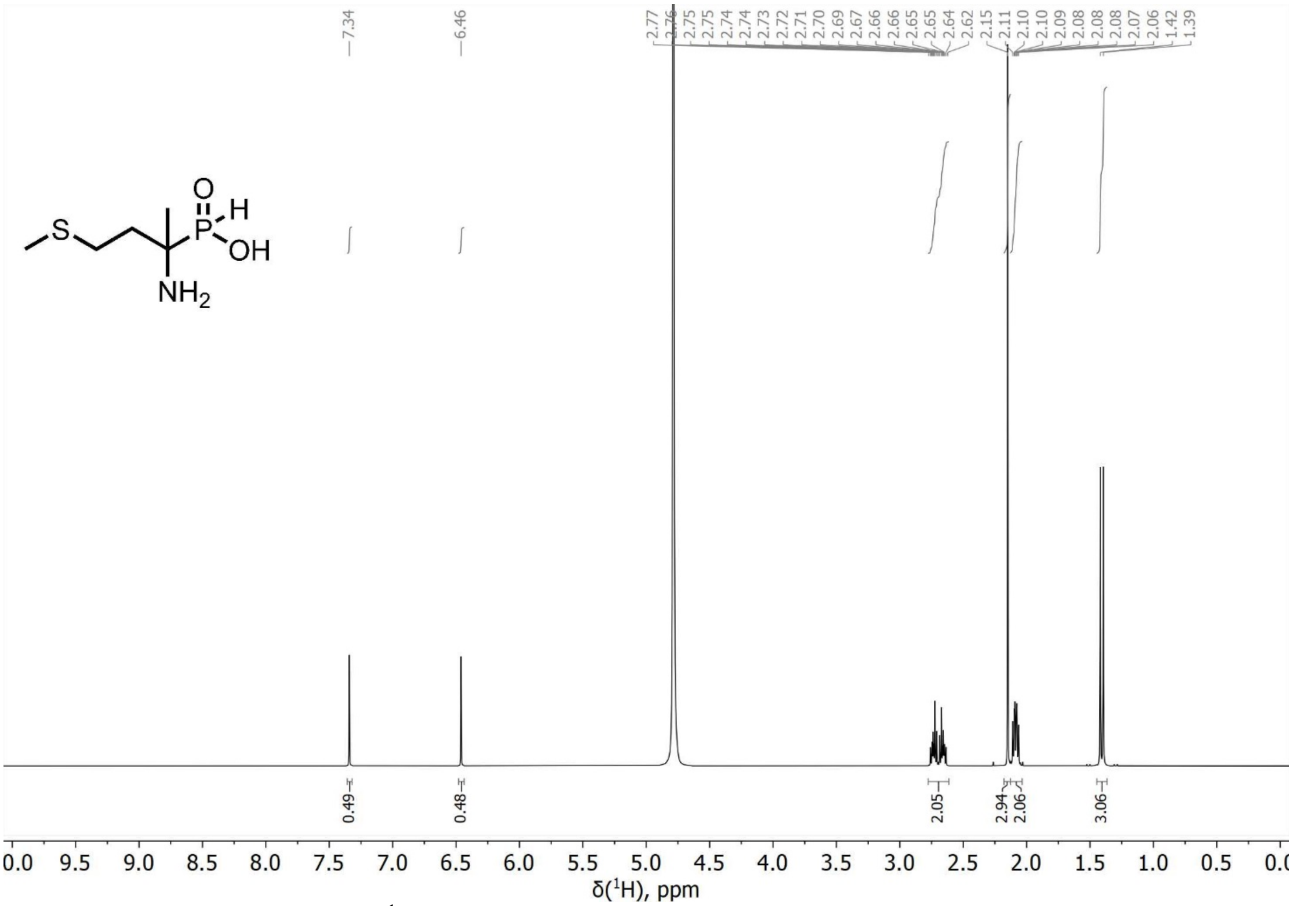

Supplementary Figure S36. ^{1}H NMR spectrum of α-CH_3-Met-P_H

^{1}H NMR (600 MHz, D_2O) δ = 6.9 (d, $^{1}J_{PH}$=529.0, 1H), 2.8 – 2.6 (m, 2H), 2.1 (s, 3H), 2.1 (ddd, *J*=11.7, 9.3, 7.4, 2H), 1.4 (d, *J*=14.4, 3H).

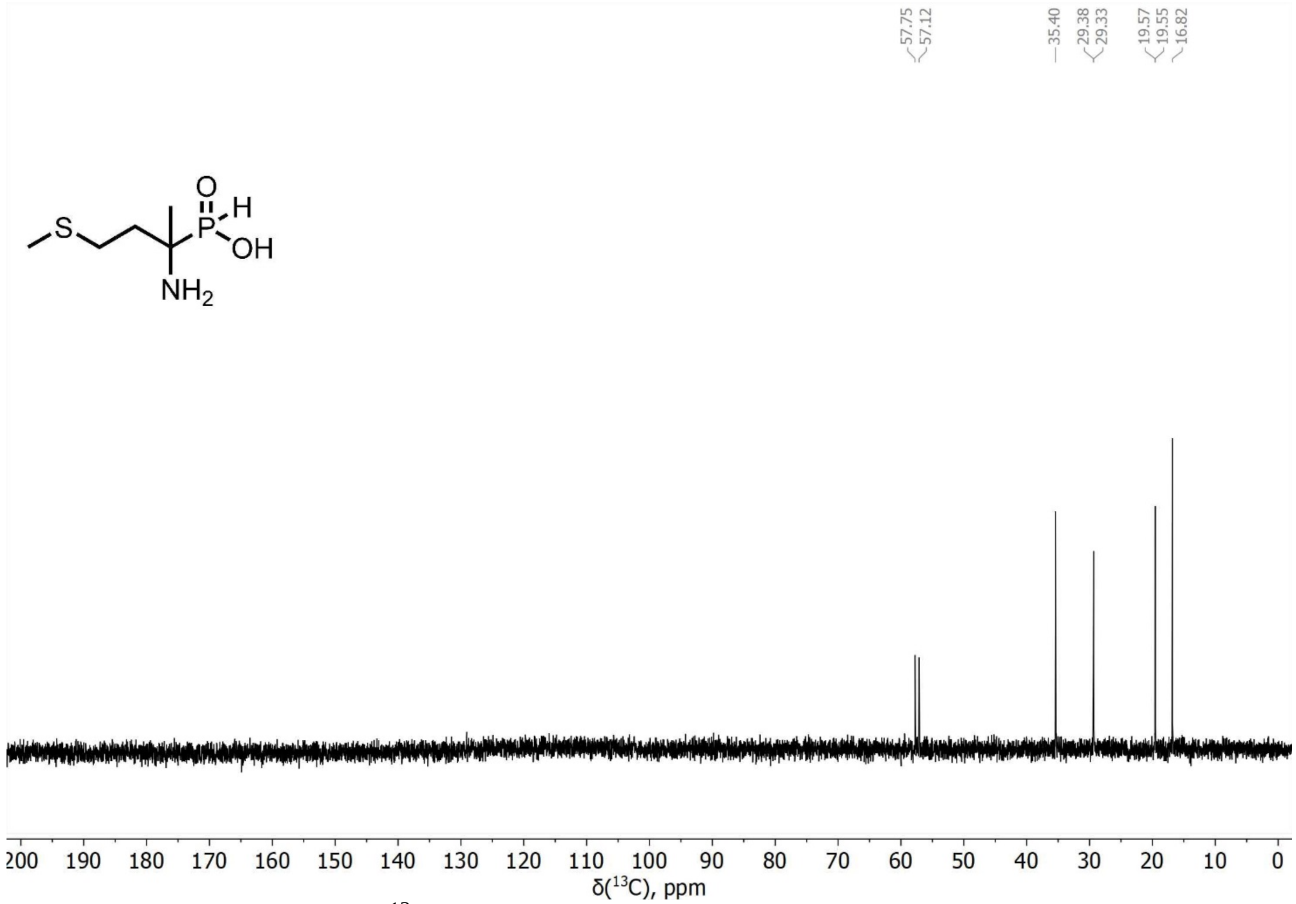

Supplementary Figure S37. ^{13}C NMR spectrum of α-CH_3-Met-P_H

^{13}C NMR (151 MHz, D_2O) δ = 57.4 (d, *J*=94.9), 35.4, 29.4 (d, *J*=7.1), 19.6 (d, *J*=2.5), 16.8.

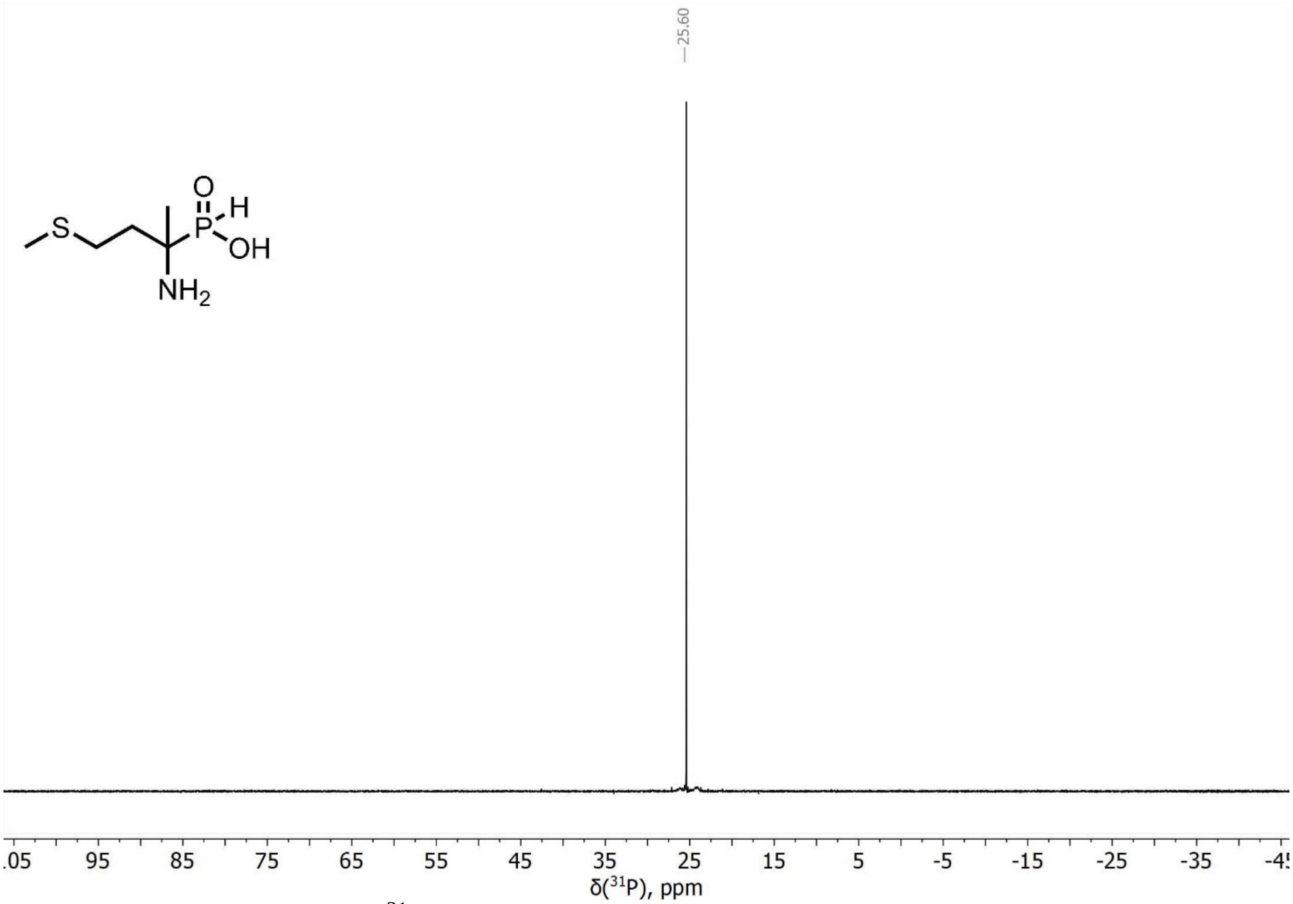

Supplementary Figure S38. ^{31}P NMR spectrum of α-CH_3-Met-P_H

^{31}P NMR (243 MHz, D_2O) δ = 25.6.

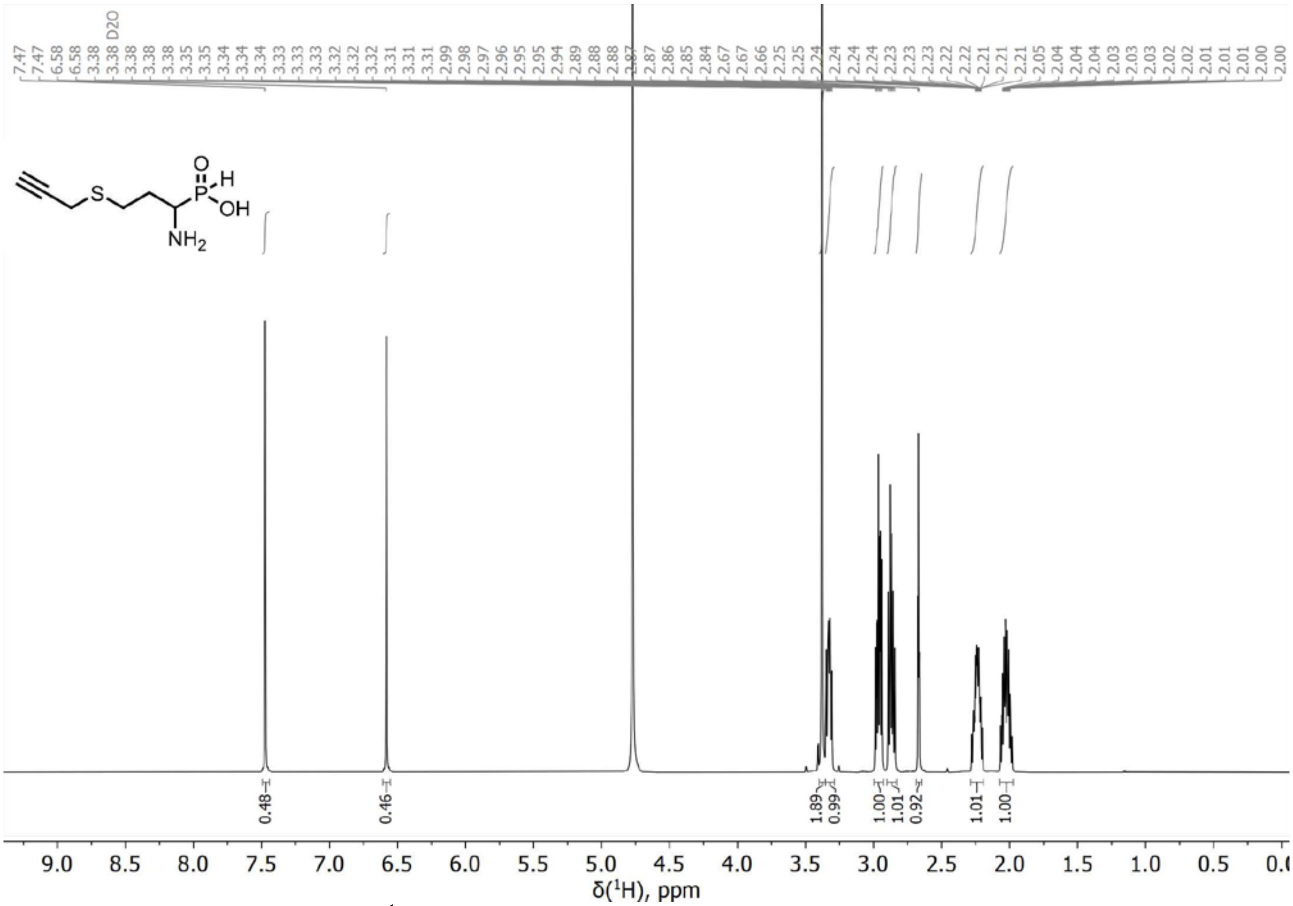

Supplementary Figure S39. ^{1}H NMR spectrum of Pro-S-Hcy-P_H

^{1}H NMR (600 MHz, D_2O) δ = 7.0 (dd, $^1J_{PH}$=535.2, 1.4, 1H), 3.4 (dd, *J*=2.7, 1.1, 2H), 3.4 – 3.3 (m, 1H), 3.0 (ddd, *J*=14.1, 8.5, 5.9, 1H), 2.9 (ddd, *J*=13.9, 8.2, 6.9, 1H), 2.7 (t, *J*=2.6, 0H), 2.3 – 2.2 (m, 1H), 2.0 (d, *J*=534.7, 1H).

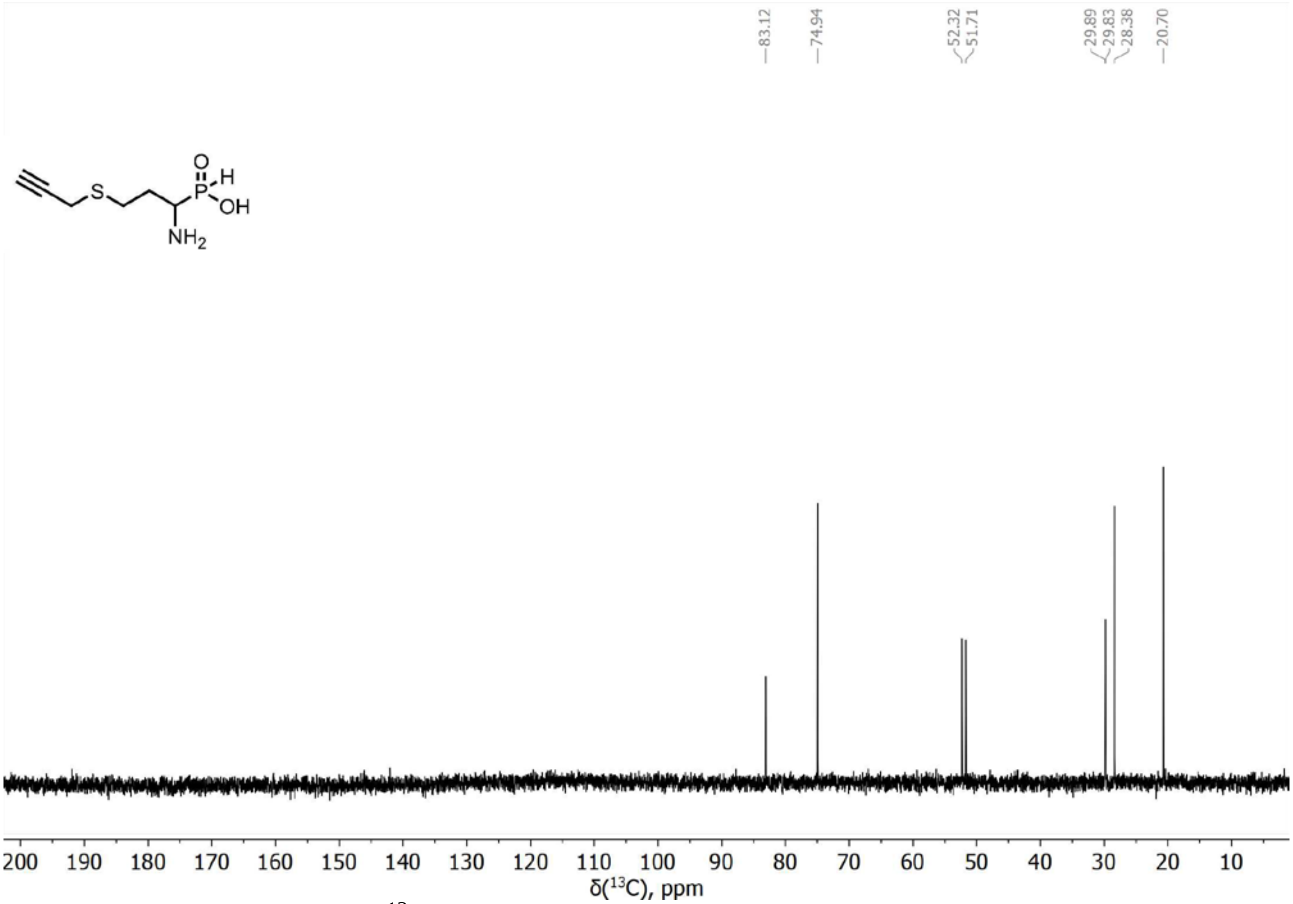

Supplementary Figure S40. ^{13}C NMR spectrum of Pro-S-Hcy-P_H

^{13}C NMR (151 MHz, D_2O) δ = 83.1, 74.9, 52.0 (d, *J*=91.2), 29.9 (d, *J*=9.7), 28.4, 20.7

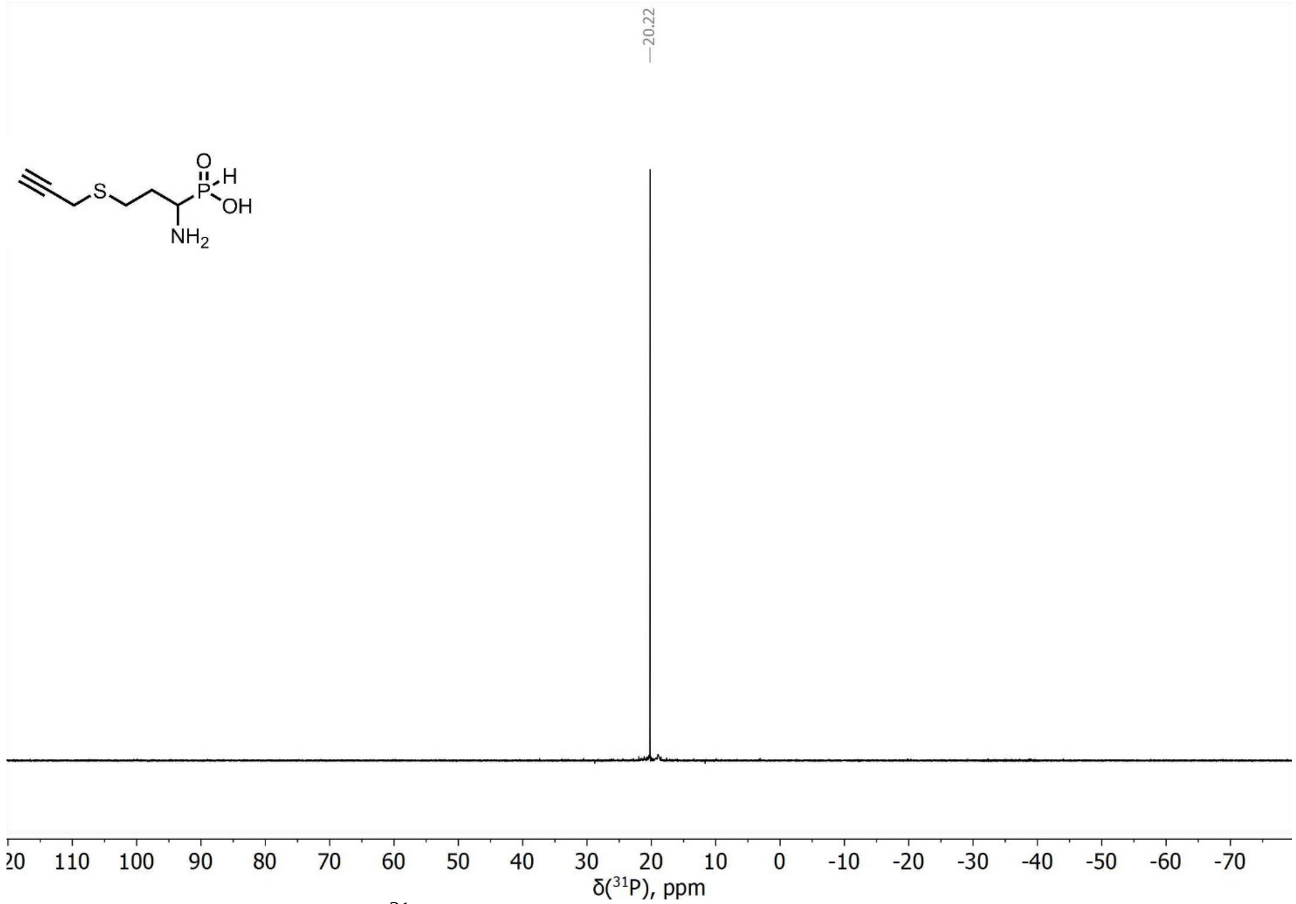

Supplementary Figure S41. ^{31}P NMR spectrum of Pro-S-Hcy-P_H

^{31}P NMR (243 MHz, D_2O) δ = 20.2.

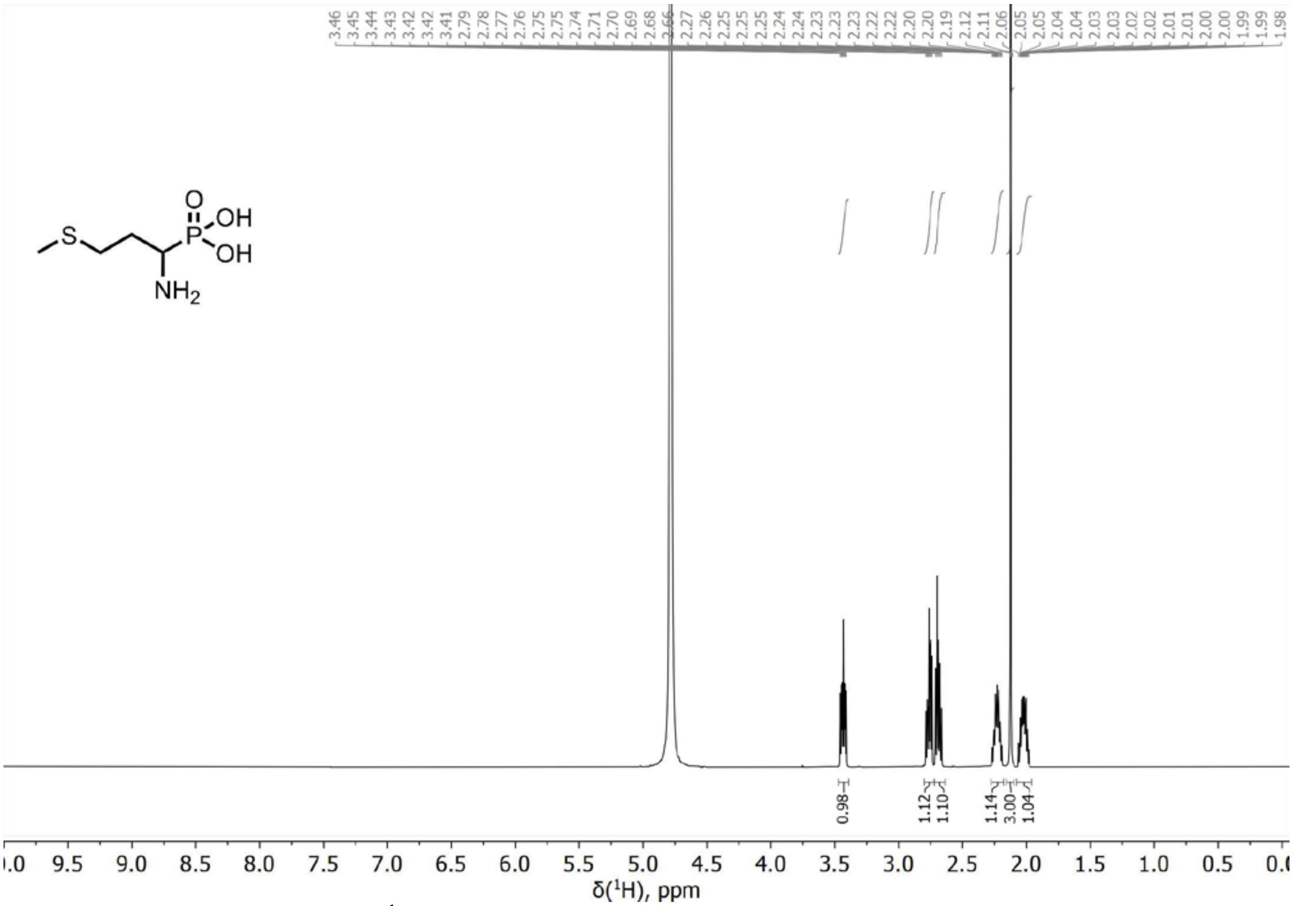

Supplementary Figure S42. ^{1}H NMR spectrum of *rac*-Met-P_5

^{1}H NMR (600 MHz, D_2O) δ = 3.5 – 3.4 (m, 1H), 2.8 – 2.6 (m, 2H), 2.3 – 2.2 (m, 1H), 2.1 (s, 3H), 2.1 – 2.0 (m, 1H).

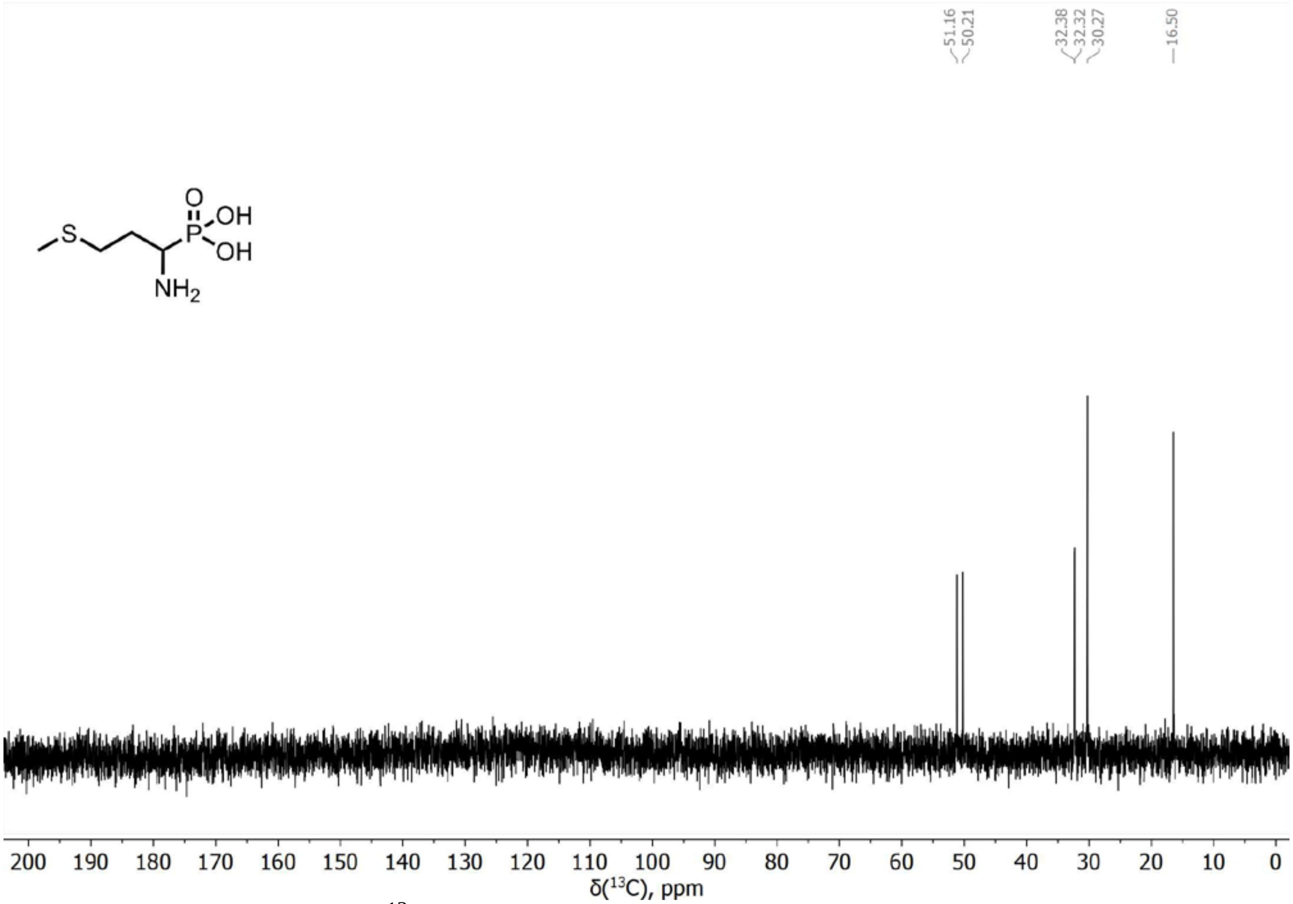

Supplementary Figure S43. ^{13}C NMR spectrum of *rac*-Met-P_5

^{13}**C NMR** (151 MHz, D_2O) δ = 50.7 (d, *J*=142.8), 32.4 (d, *J*=10.1), 30.3, 16.5.

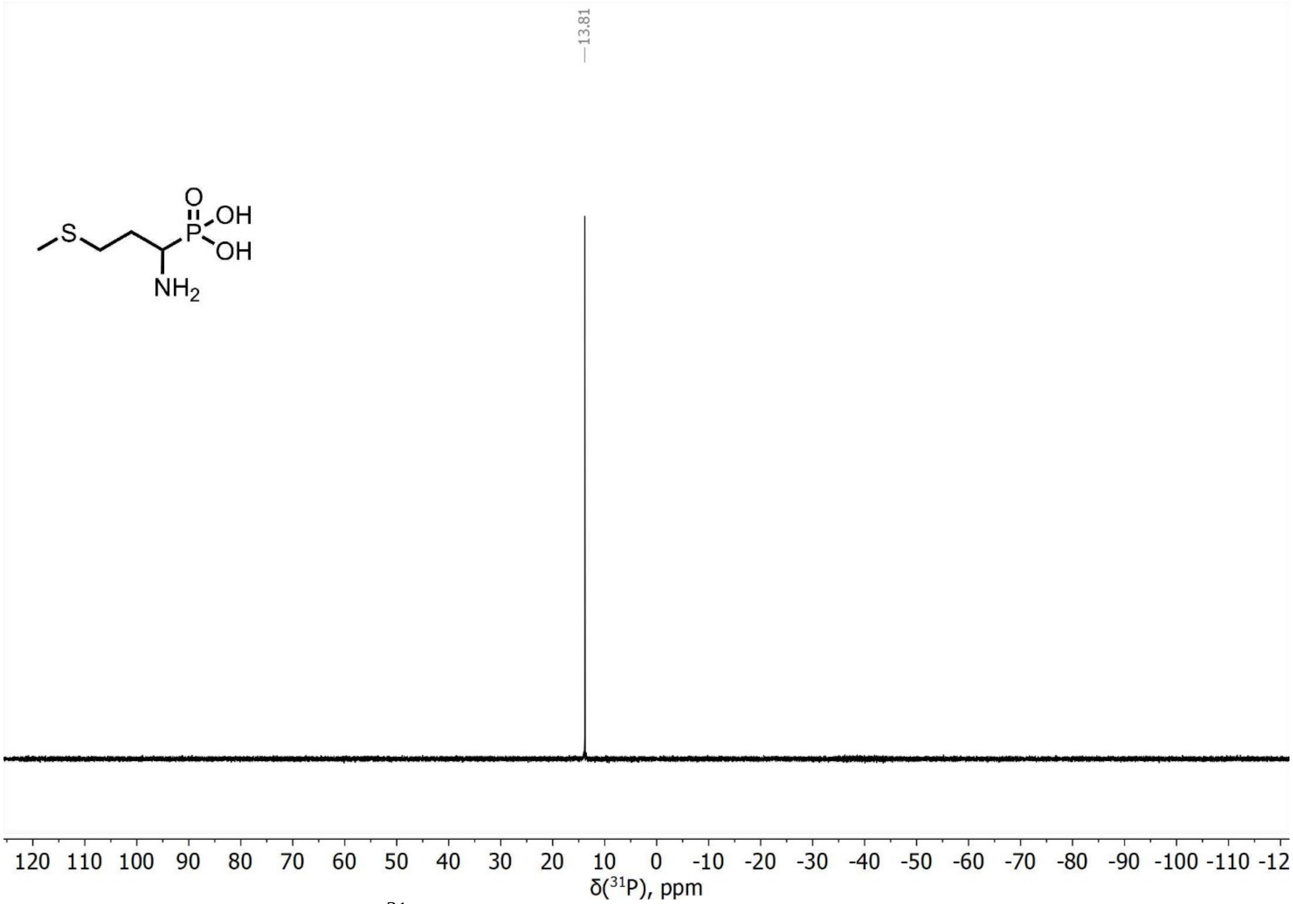

Supplementary Figure S44. ^{31}P NMR spectrum of *rac*-Met-P_5

^{31}P NMR (243 MHz, D_2O) δ = 13.8.

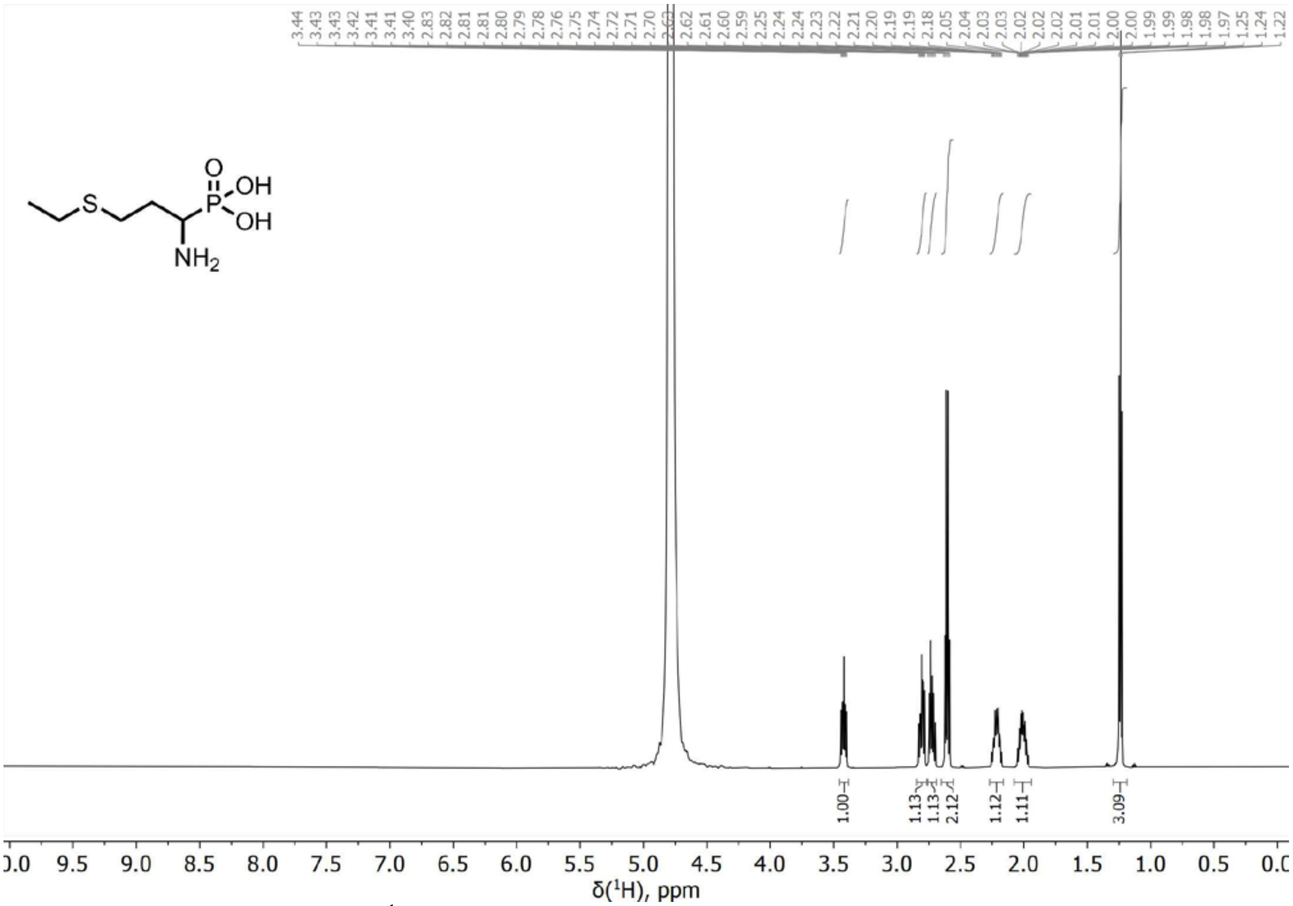

Supplementary Figure S45. ^{1}H NMR spectrum of Ethionine-P_5

^{1}H NMR (600 MHz, D_2O) δ = 3.4 (ddd, J=13.6, 8.5, 5.4, 1H), 2.8 – 2.7 (m, 2H), 2.6 (q, J=7.4, 2H), 2.3 – 2.2 (m, 1H), 2.1 – 2.0 (m, 1H), 1.2 (t, J=7.4, 3H).

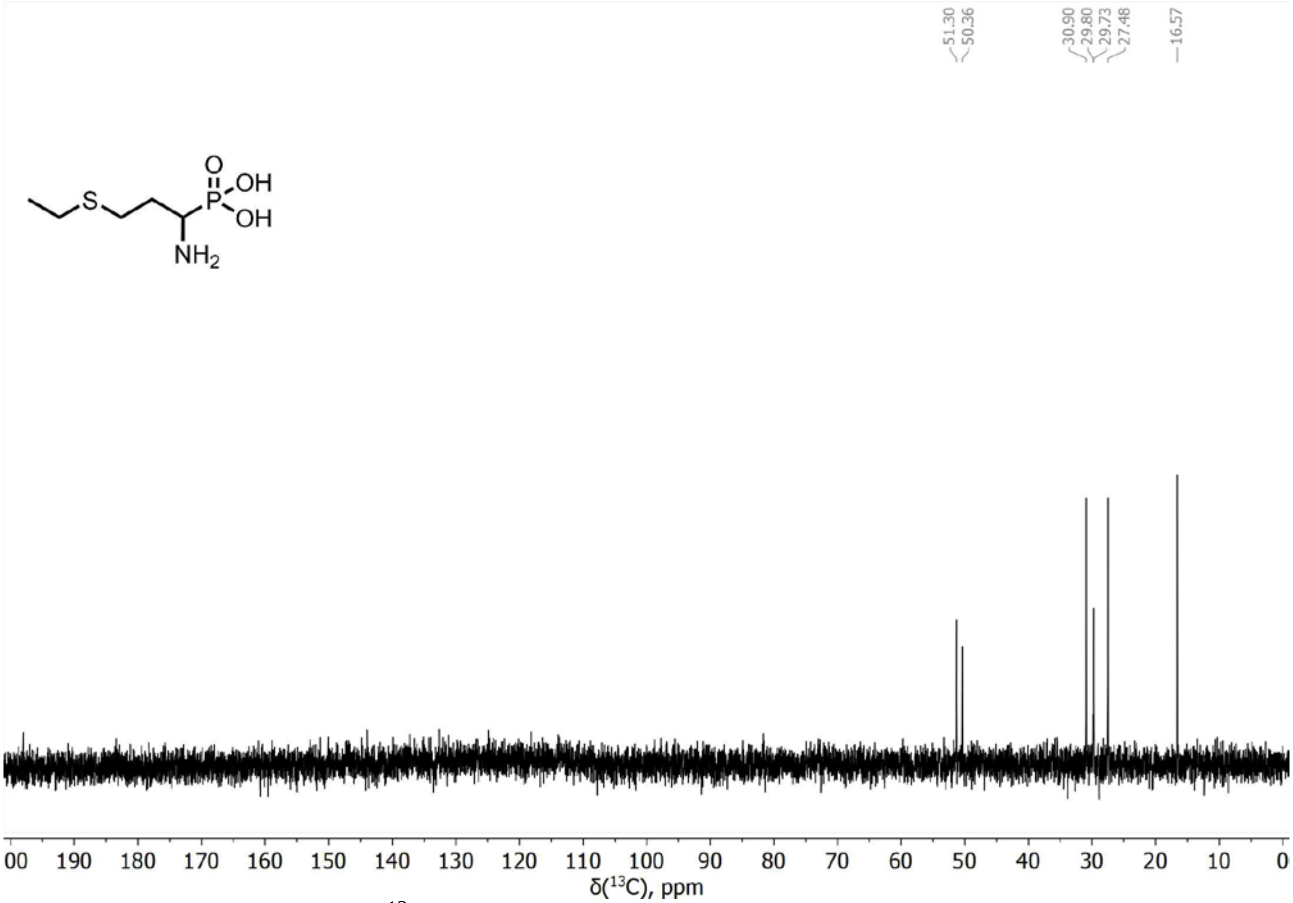

Supplementary Figure S46. ^{13}C NMR spectrum of Ethionine-P_5

^{13}C NMR (151 MHz, D_2O) δ = 50.8 (d, J=142.5), 30.9, 29.8 (d, J=10.0), 27.5, 16.6.

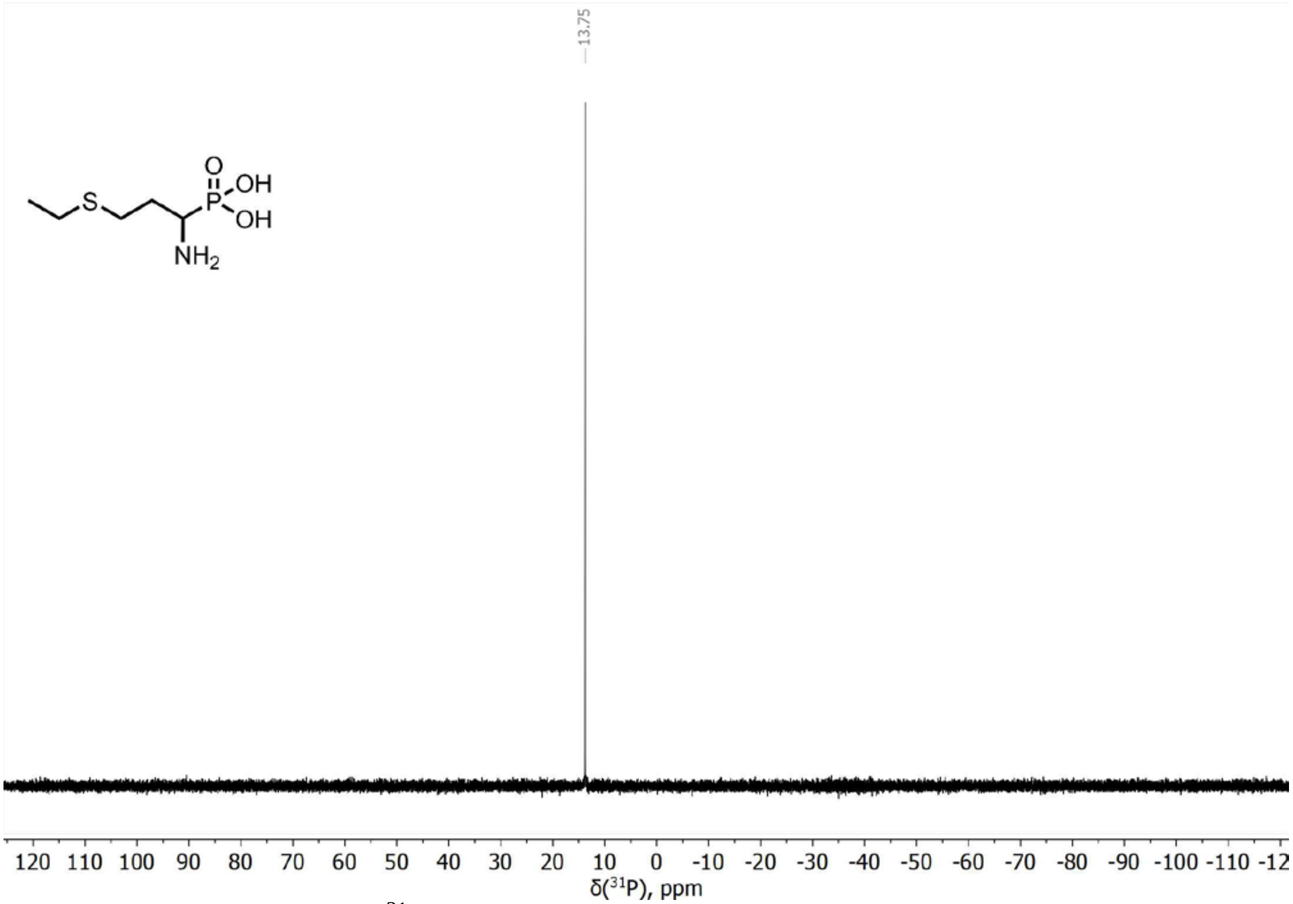

Supplementary Figure S47. ^{31}P NMR spectrum of Ethionine-P_5

^{31}P NMR (243 MHz, D_2O) δ = 13.7.

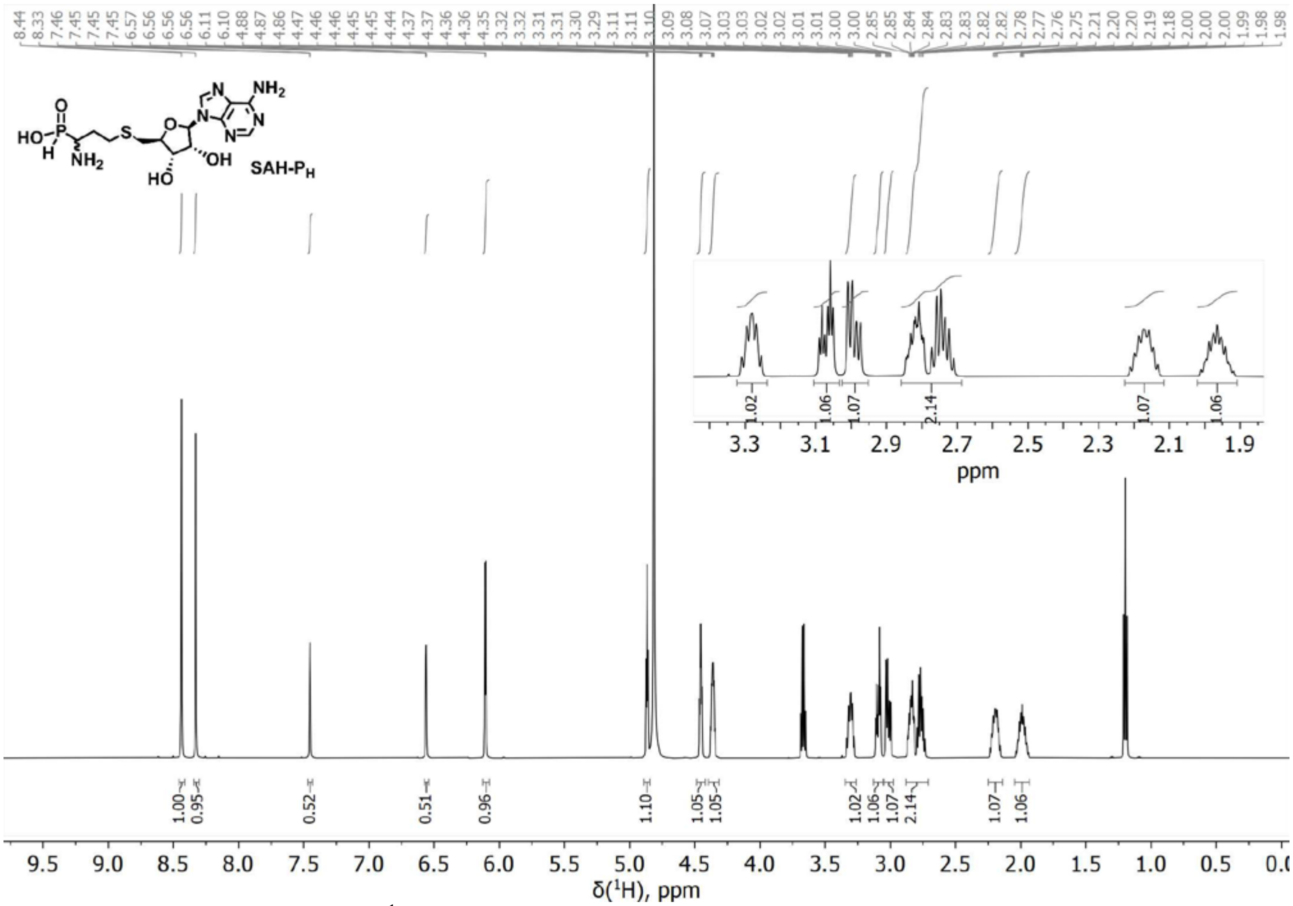

Supplementary Figure S48. ^{1}H NMR spectrum of *rac*-SAH-P_H

^{1}H NMR (600 MHz, D_2O) δ = 8.4 (s, 1H), 8.3 (s, 1H), 7.0 (d, $^{1}J_{PH}$=534.9, 1H), 6.1 (d, *J*=4.9, 1H), 4.9 (t, *J*=5.2, 1H), 4.5 – 4.4 (m, 1H), 4.4 – 4.3 (m, 1H), 3.3 – 3.3 (m, 1H), 3.1 (dt, *J*=14.2, 4.7, 1H), 3.0 (ddd, *J*=14.2, 6.9, 1.7, 1H), 2.9 – 2.7 (m, 2H), 2.2 – 2.1 (m, 1H), 2.0 – 1.9 (m, 1H).

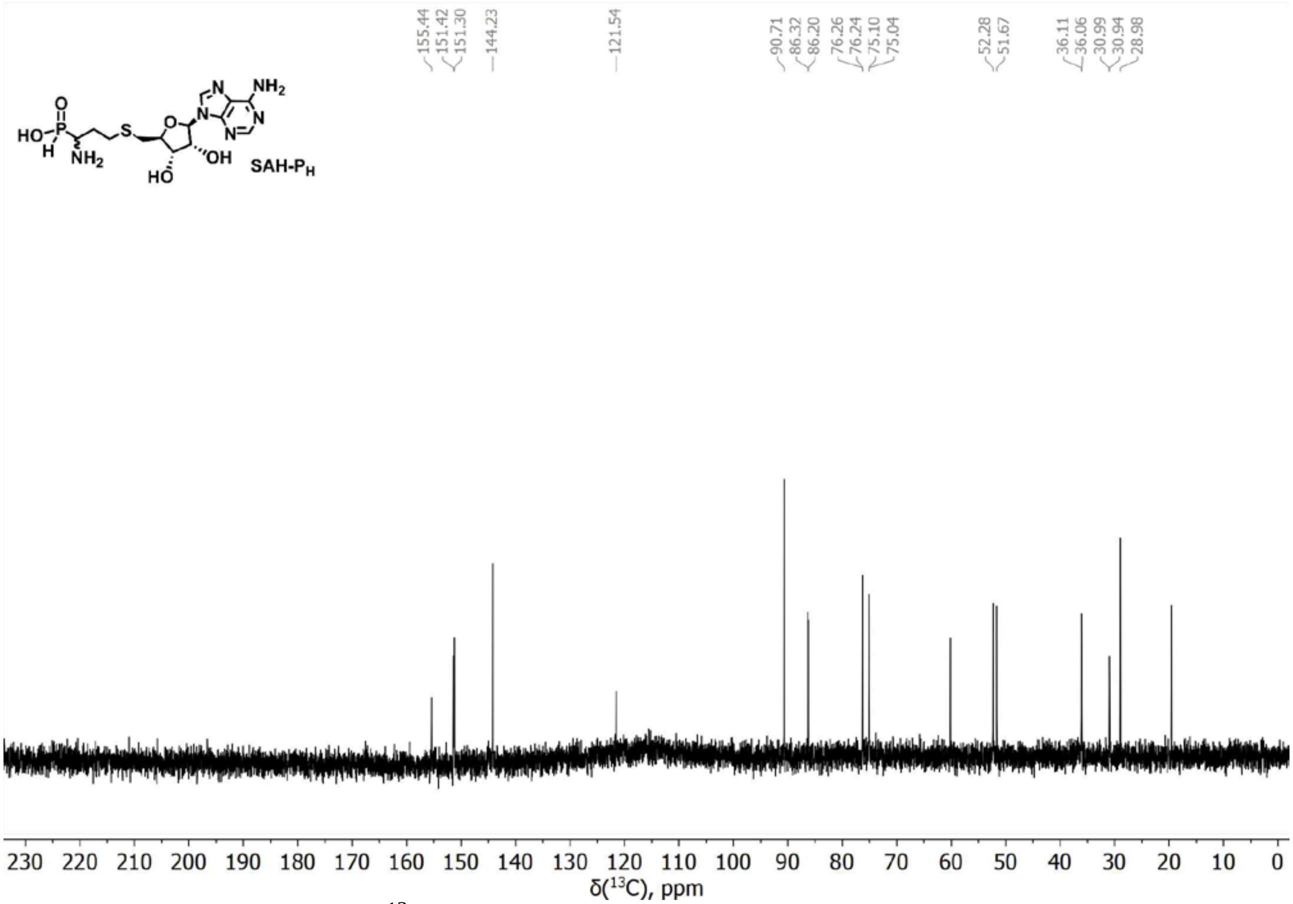

Supplementary Figure S49. ^{13}C NMR spectrum of *rac*-SAH-P_H

^{13}C NMR (151 MHz, D_2O) δ = 155.4, 151.4, 151.3, 144.2, 121.5, 90.7, 86.3 (d, *J*=18.7), 76.3 (d, *J*=3.5), 75.1 (d, *J*=9.6), 52.0 (d, *J*=90.9), 36.1 (d, *J*=7.8), 31.0 (d, *J*=8.5), 29.0.

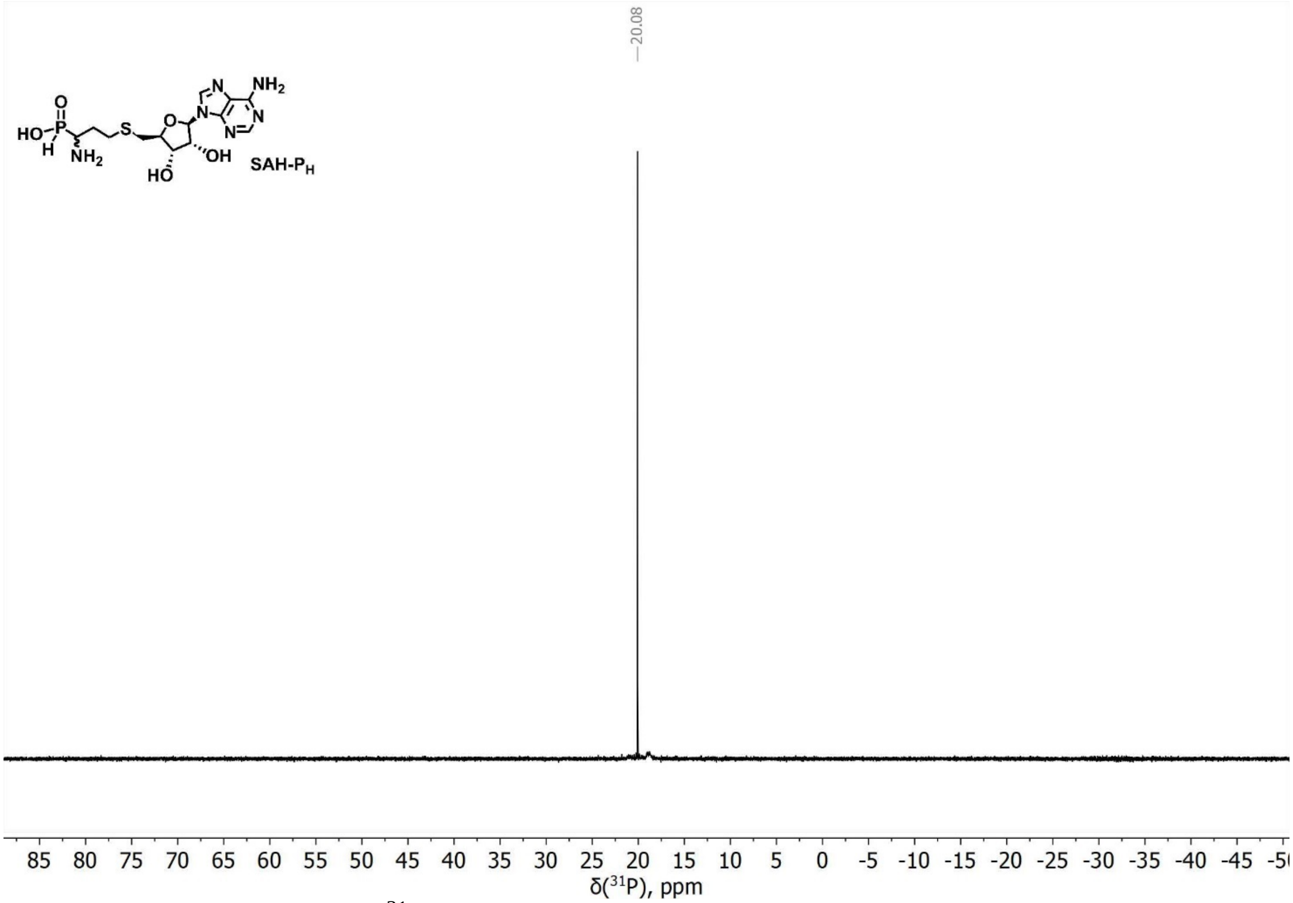

Supplementary Figure S50. ^{31}P NMR spectrum of *rac*-SAH-P_H

^{31}P NMR (243 MHz, D_2O) δ = 20.1.

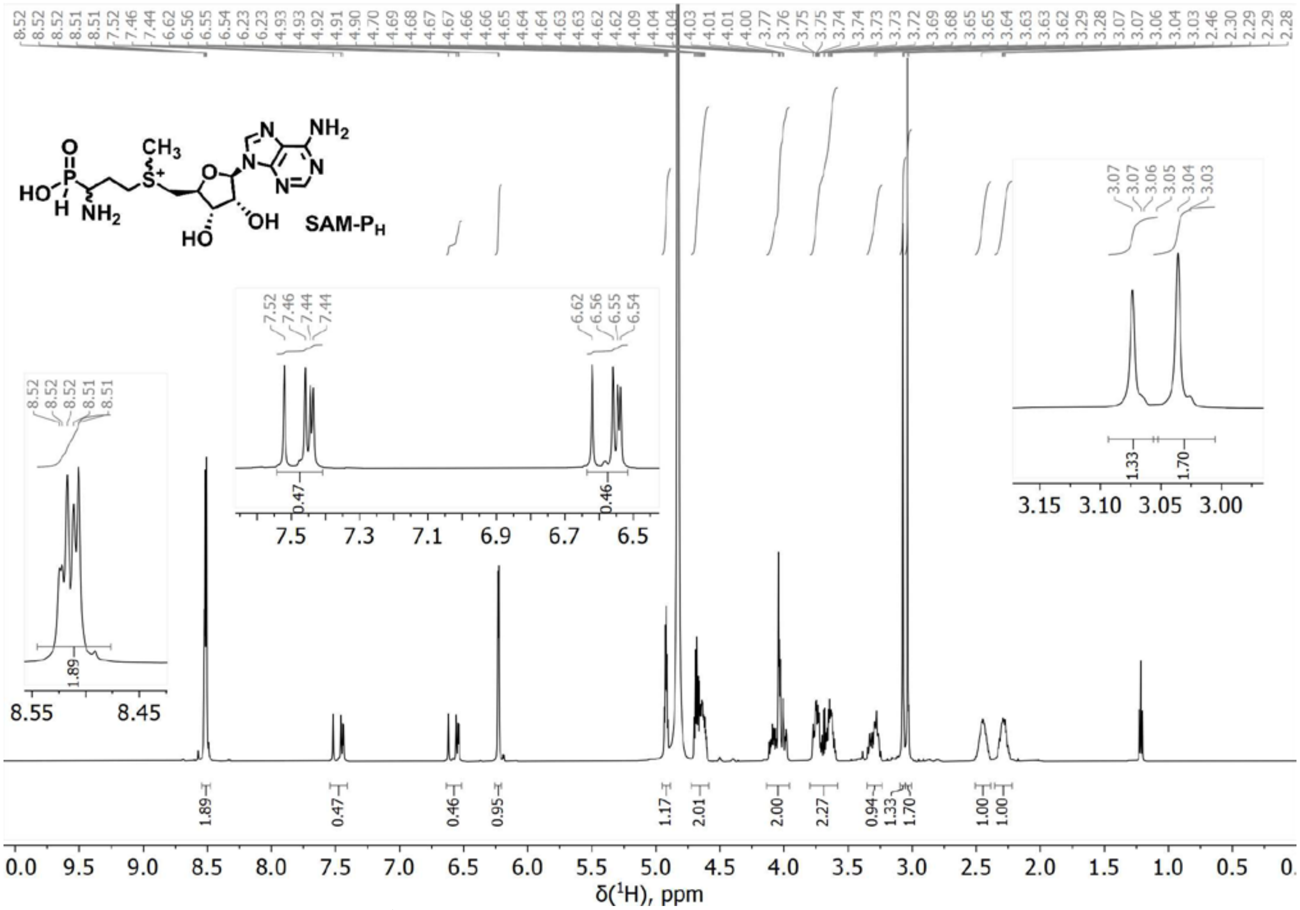

Supplementary Figure S51. ^{1}H NMR spectrum of *rac*-SAM-P_H

^{1}H NMR (600 MHz, D_2O) δ = 8.4 (s, 1H), 8.3 (s, 1H), 7.0 (d, *J*=534.9, 1H), 6.1 (d, *J*=4.9, 1H), 4.9 (t, *J*=5.2, 1H), 4.5 – 4.4 (m, 1H), 4.4 – 4.3 (m, 1H), 3.3 – 3.3 (m, 1H), 3.1 (dt, *J*=14.2, 4.7, 1H), 3.0 (ddd, *J*=14.2, 6.9, 1.7, 1H), 2.9 – 2.7 (m, 2H), 2.2 – 2.1 (m, 1H), 2.0 – 1.9 (m, 1H).

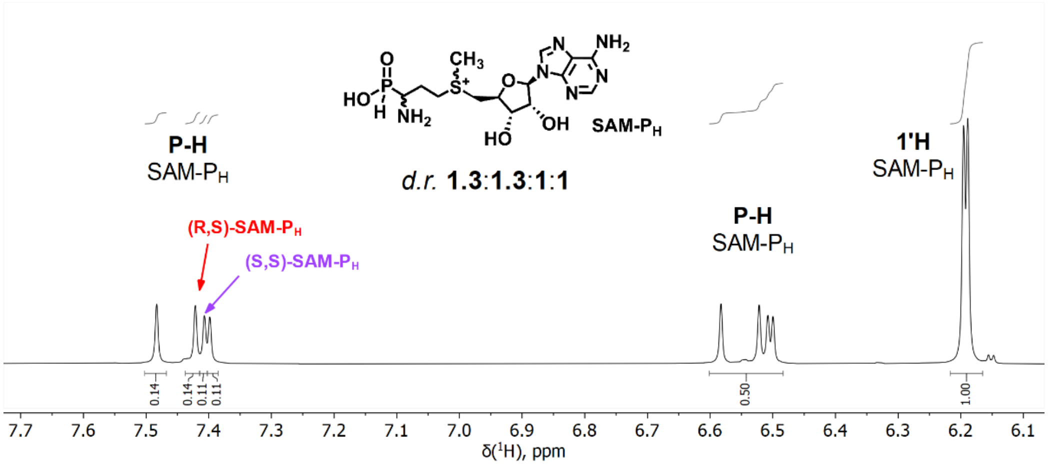

Supplementary Figure S52. The fragment of PH signals of ^{1}H NMR spectrum of various stereoisomers of the *rac*-SAM-P_H. The diastereomeric ratio (*d.r.*) of various stereoisomers 1.3:1.3:1:1. Stereoisomers *(R,S)*-**SAM-P_H** and *(S,S)*-**SAM-P_H** are indicated by arrows (see Supplementary Figure S10).

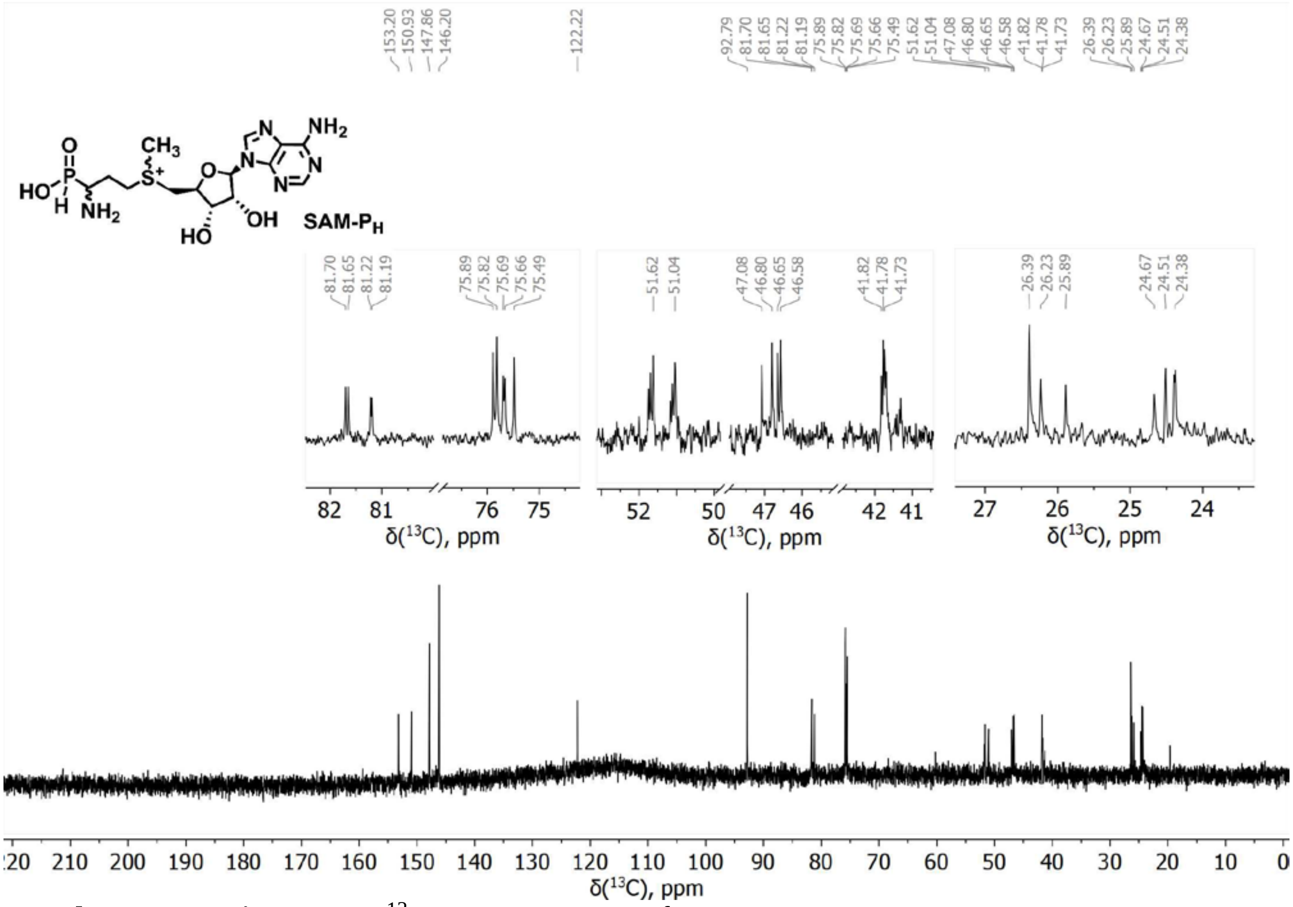

Supplementary Figure S53. ^{13}C NMR spectrum of *rac*-SAM-P$_H$

^{13}C NMR (151 MHz, D_2O) δ = 153.2, 151.0 – 150.8 (m), 148.0 – 147.7 (m), 146.4 – 146.0 (m), 122.2, 92.9 – 92.7 (m), 81.9 – 81.1 (m), 76.1 – 75.8 (m), 75.8 – 75.4 (m), 51.7 – 50.9 (m), 47.4 – 46.4 (m), 42.3 – 41.5 (m), 26.8 – 25.8 (m), 24.9 – 24.1 (m).

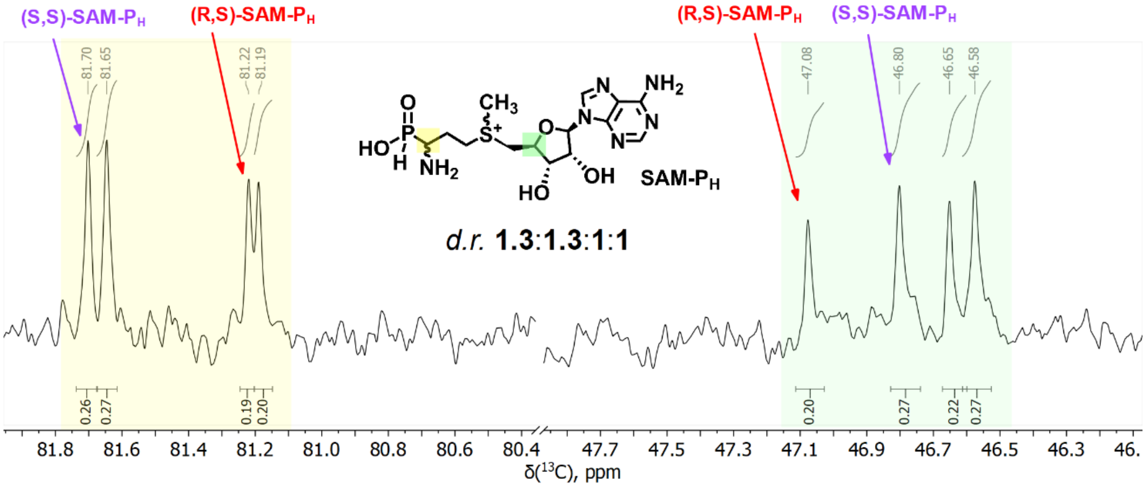

Supplementary Figure S54. The fragment of ^{13}C NMR spectrum of *rac*-SAM-P$_H$. The diastereomeric ratio (*d.r.*) of various stereoisomers 1.3:1.3:1:1. Stereoisomers *(R,S)*-**SAM-P$_H$** and *(S,S)*-**SAM-P$_H$** are indicated by arrows (see Supplementary Figure S11).

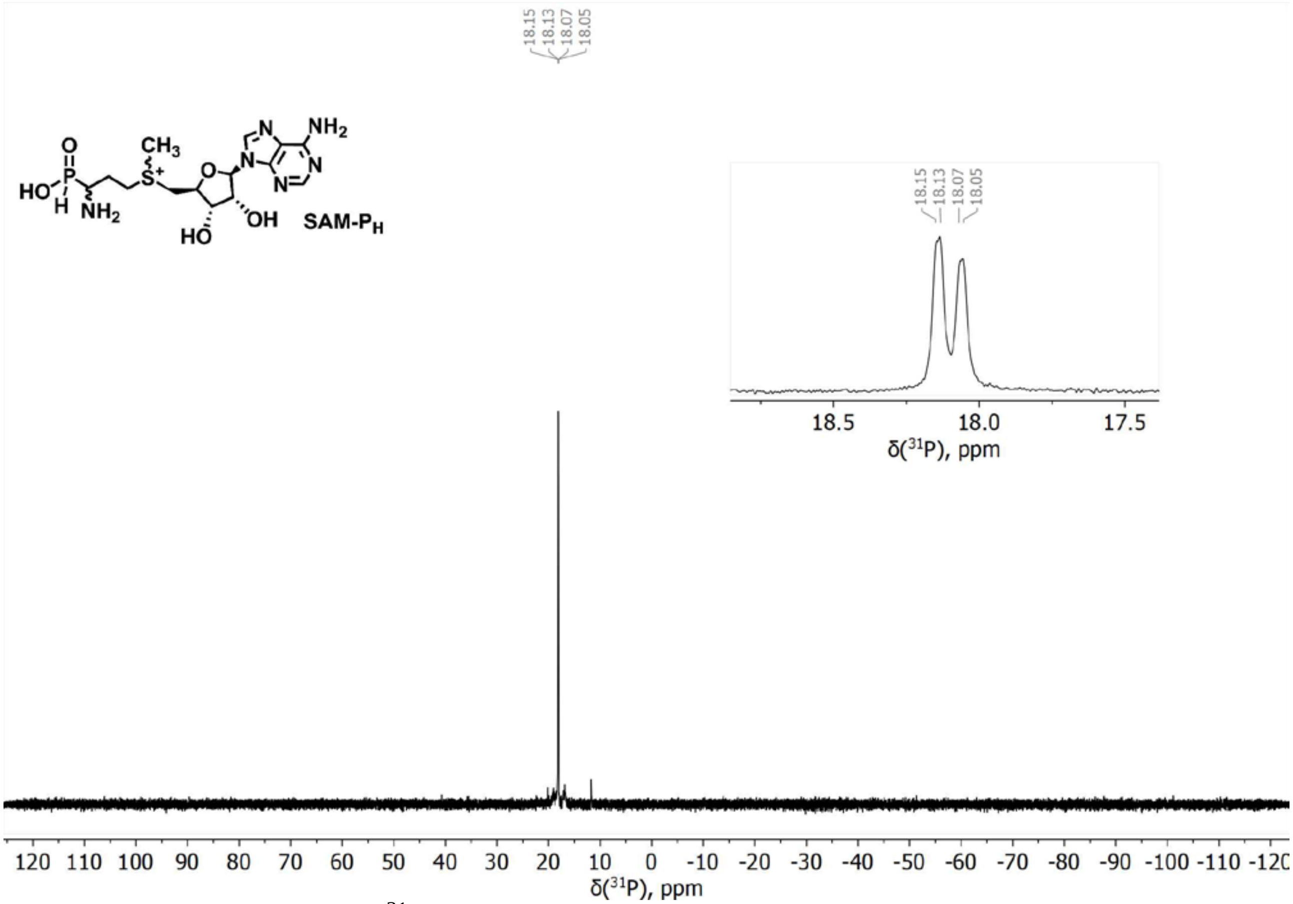

Supplementary Figure S55. ^{31}P NMR spectrum of *rac*-SAM-P_H

^{31}P NMR (243 MHz, D_2O) δ = 18.2, 18.1.

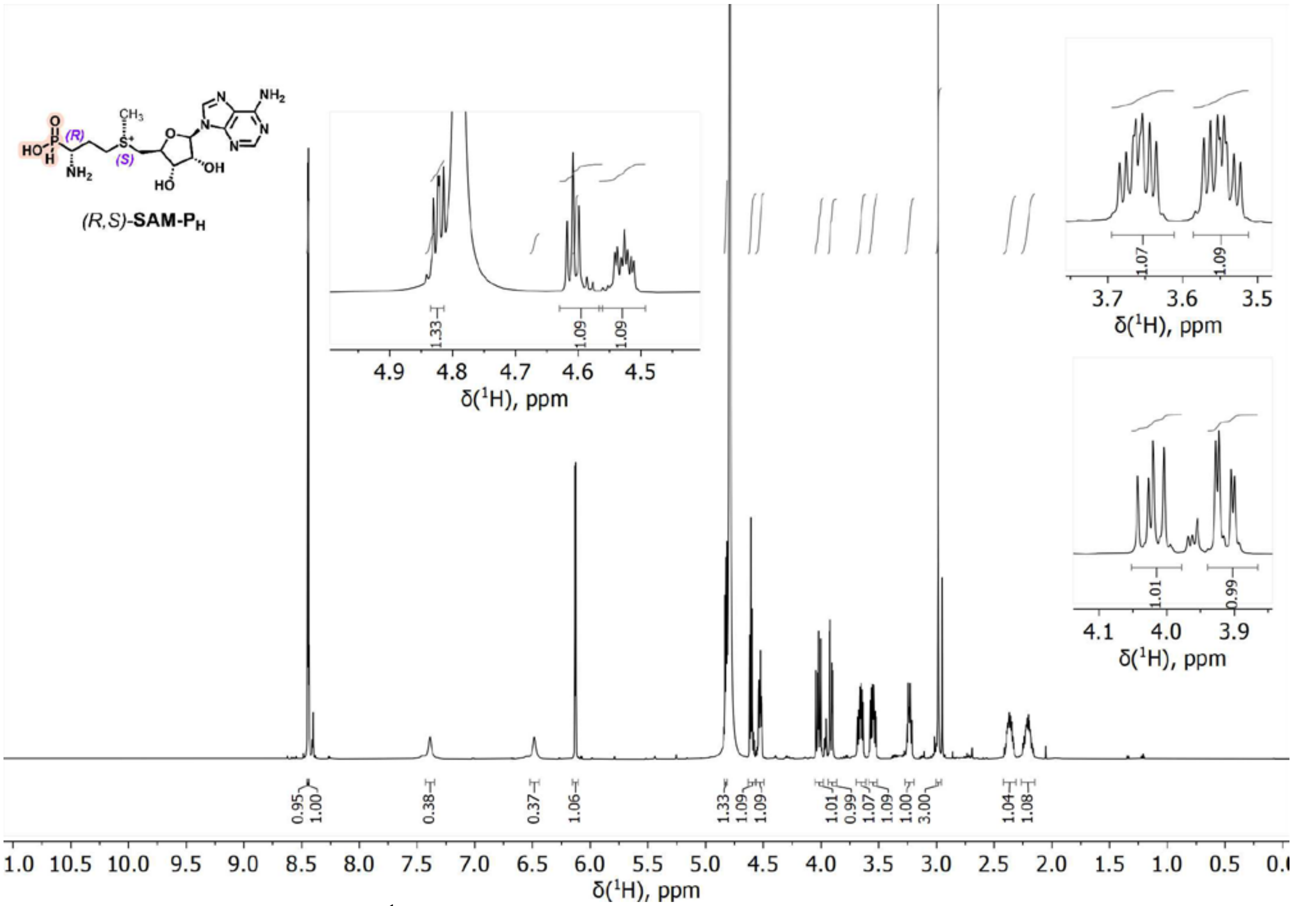

Supplementary Figure S56. ^{1}H NMR spectrum of *(R,S)*-SAM-P_H

^{1}H NMR (600 MHz, D_2O) δ = 8.4 (s, 1H), 8.4 (s, 1H), 6.9 (d, *J*=542.8, 1H), 6.1 (d, *J*=4.0, 1H), 4.9 – 4.8 (m, 1H), 4.6 – 4.5 (m, 1H), 4.6 – 4.5 (m, 1H), 4.0 (dd, *J*=13.8, 9.5, 1H), 3.9 (dd, *J*=13.8, 2.8, 1H), 3.7 – 3.6 (m, 1H), 3.6 – 3.5 (m, 1H), 3.3 – 3.2 (m, 1H), 3.0 (s, 3H), 2.4 – 2.3 (m, 1H), 2.3 – 2.1 (m, 1H).

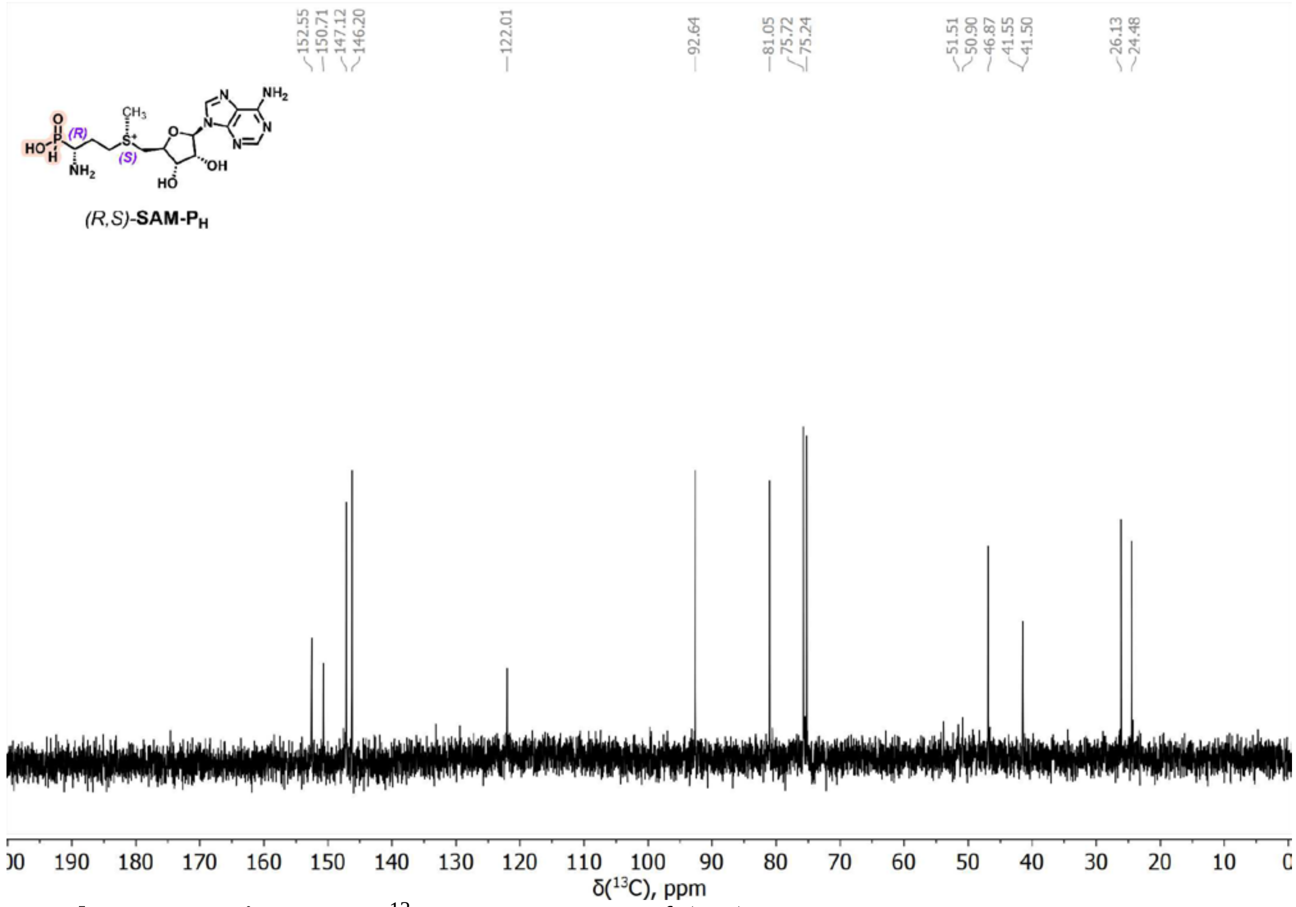

Supplementary Figure S57. ^{13}C NMR spectrum of *(R,S)*-SAM-P$_H$

^{13}C NMR (151 MHz, D_2O) δ = 152.6, 150.7, 147.1, 146.2, 122.0, 92.6, 81.0, 75.7, 75.2, 51.2 (d, *J*=92.7), 46.9, 41.5 (d, *J*=7.3), 26.1, 24.5.

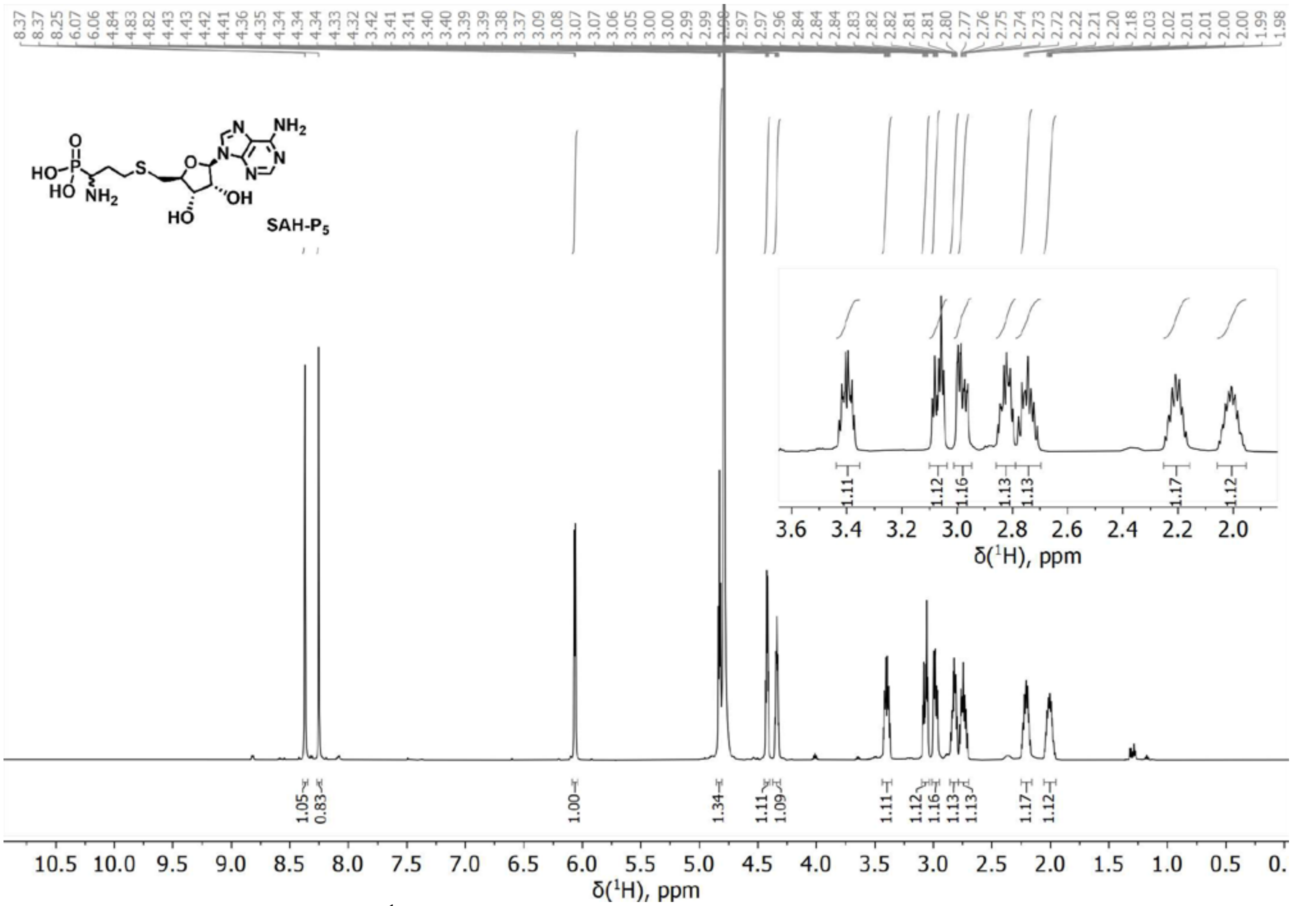

Supplementary Figure S58. ^{1}H NMR spectrum of *rac*-SAH-P_5

^{1}H NMR (600 MHz, D_2O) δ = 8.4 (s, 1H), 8.3 (s, 1H), 6.1 (d, *J*=5.0, 1H), 4.8 (t, *J*=5.2, 1H), 4.4 (q, *J*=4.7, 1H), 4.3 (q, *J*=4.6, 1H), 3.4 – 3.4 (m, 1H), 3.1 – 3.0 (m, 1H), 3.0 – 2.9 (m, 1H), 2.9 – 2.7 (m, 2H), 2.3 – 2.2 (m, 1H), 2.1 – 1.9 (m, 1H).

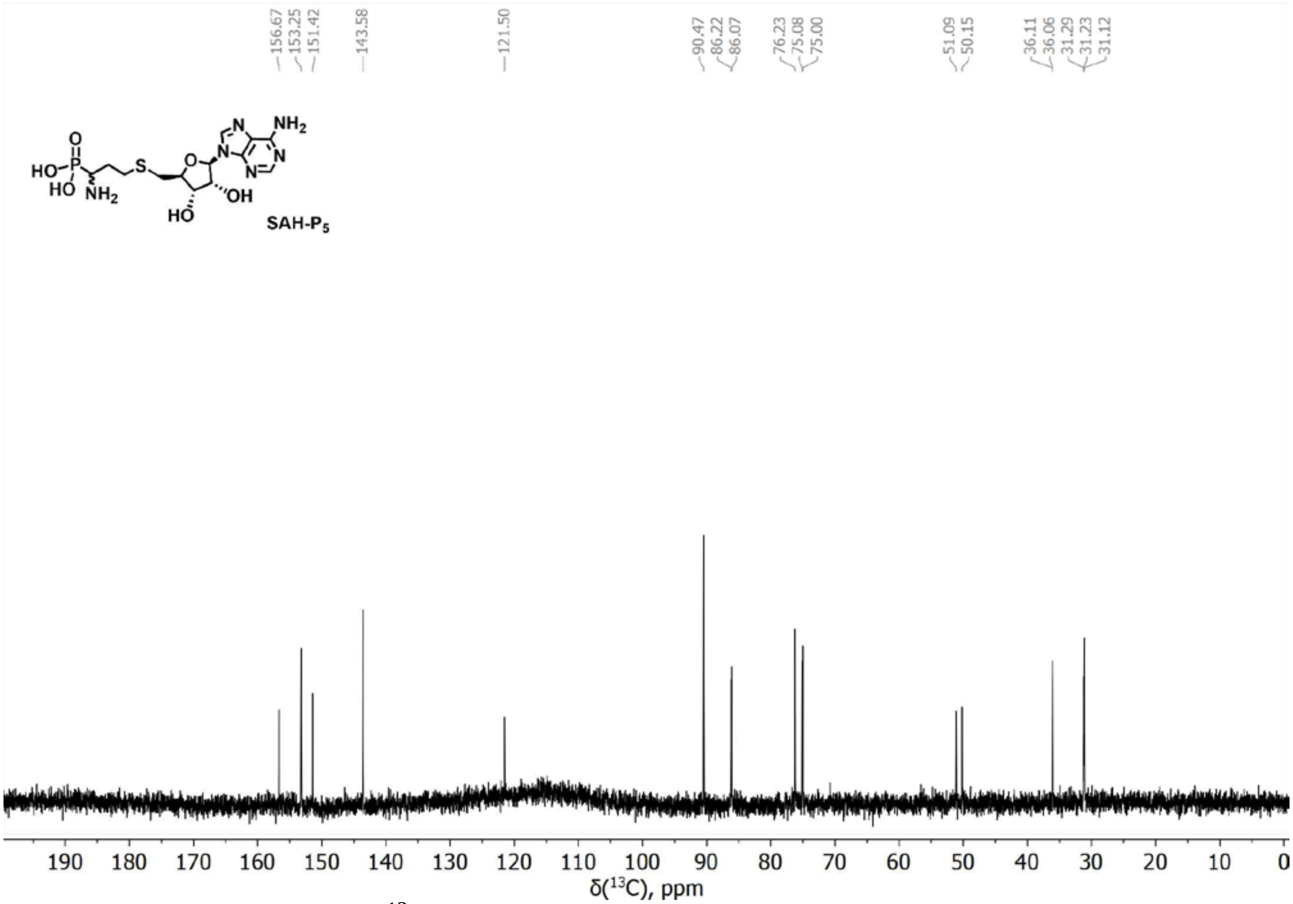

Supplementary Figure S59. ^{13}C NMR spectrum of *rac*-SAH-P5

^{13}C NMR (151 MHz, D_2O) δ = 156.7, 153.2, 151.4, 143.6, 121.5, 90.5, 86.6 – 85.6 (m), 76.2, 75.2 – 74.9 (m), 50.6 (d, *J*=142.3), 36.1 (d, *J*=8.2), 31.3 – 30.9 (m).

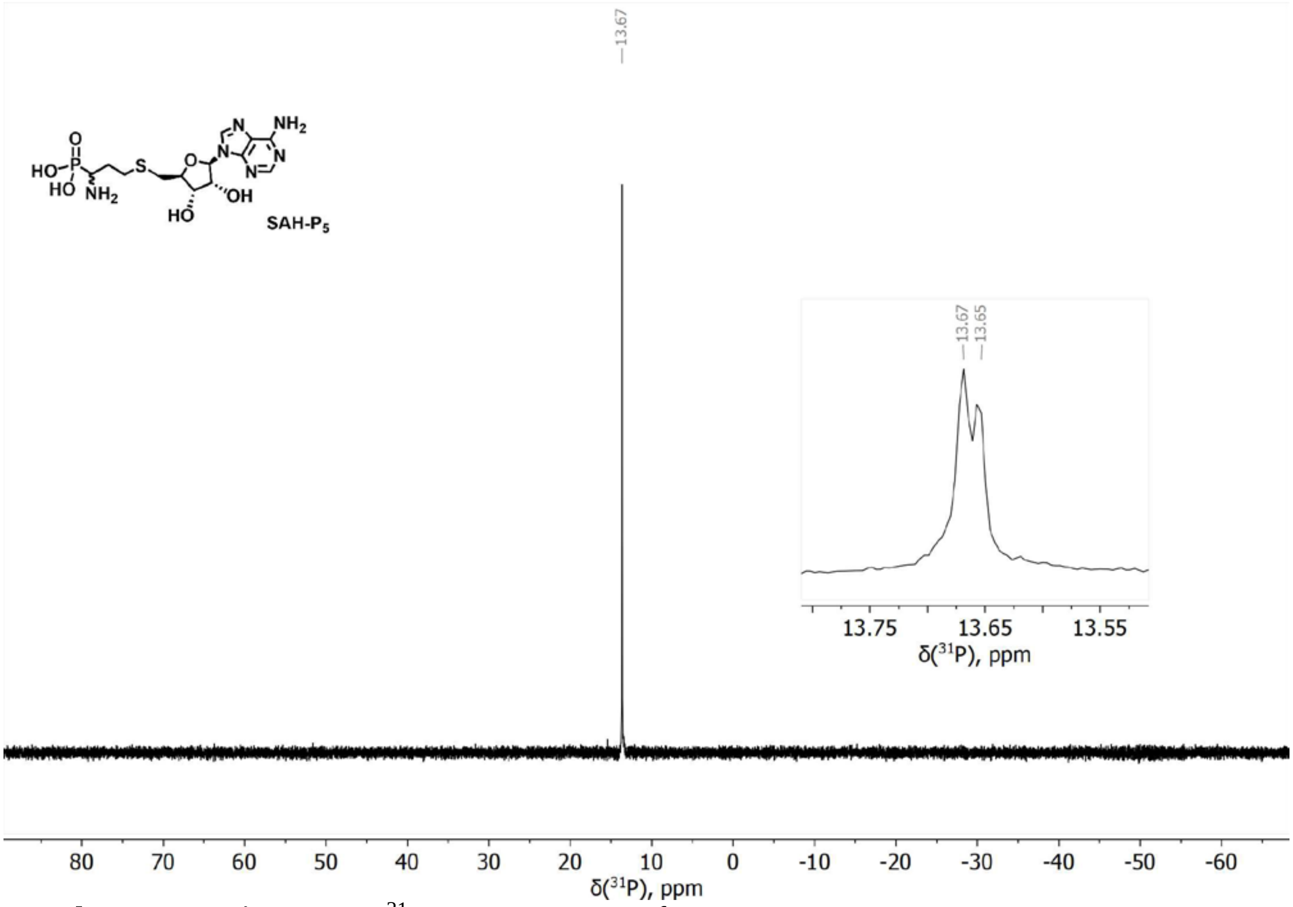

Supplementary Figure S60. ^{31}P NMR spectrum of *rac*-SAH-P_5

^{31}P NMR (243 MHz, D_2O) δ = 16.1 – 13.1 (m).

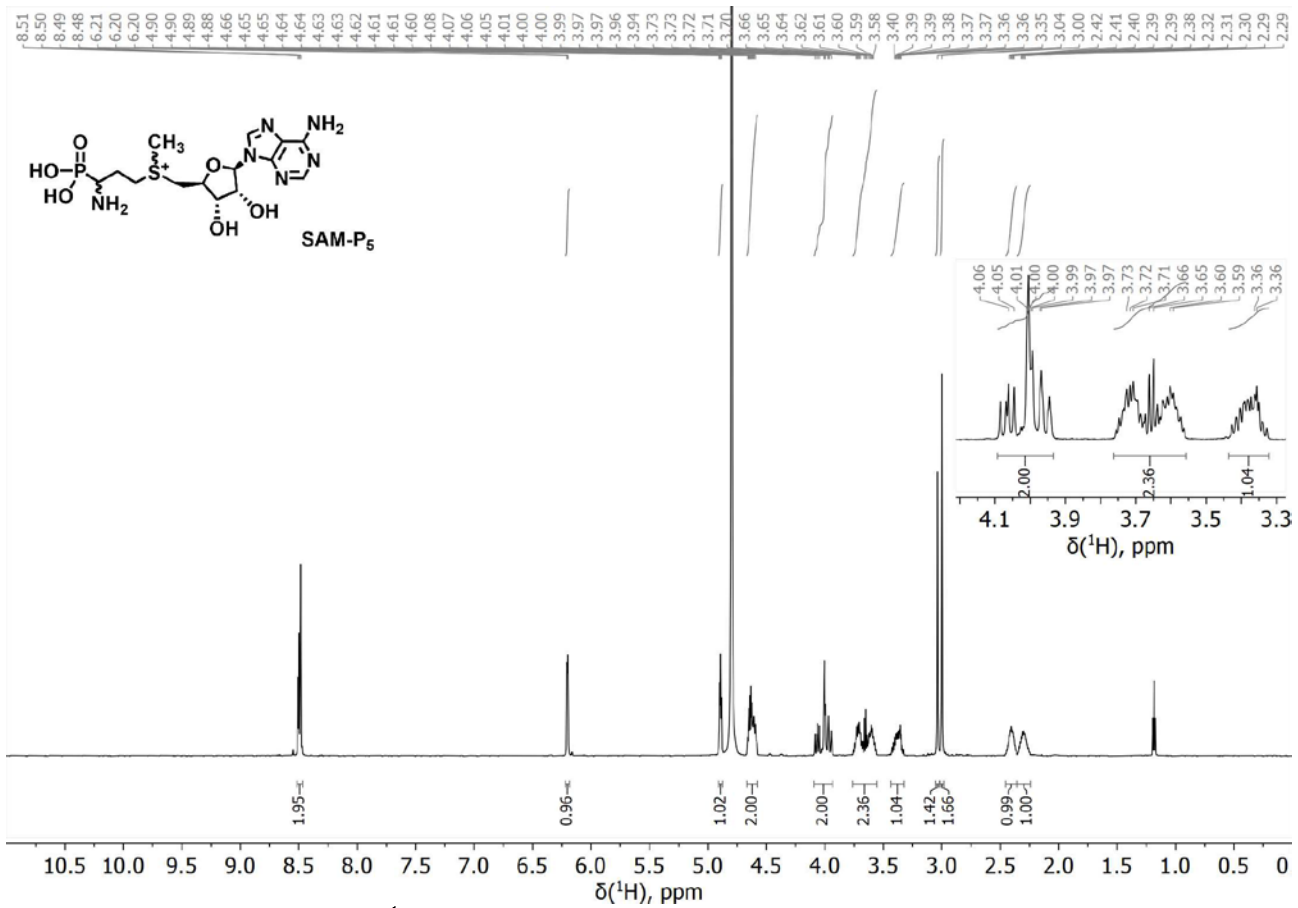

Supplementary Figure S61. ^{1}H NMR spectrum of *rac*-SAM-P_5

^{1}H NMR (600 MHz, D_2O) δ = 8.5 – 8.5 (m, 2H), 6.2 (d, *J*=3.8, 1H), 4.9 – 4.9 (m, 1H), 4.7 – 4.6 (m, 2H), 4.1 – 3.9 (m, 2H), 3.8 – 3.5 (m, 2H), 3.4 – 3.3 (m, 1H), 3.1 – 3.0 (m, 3H), 2.5 – 2.4 (m, 1H), 2.4 – 2.2 (m, 1H).

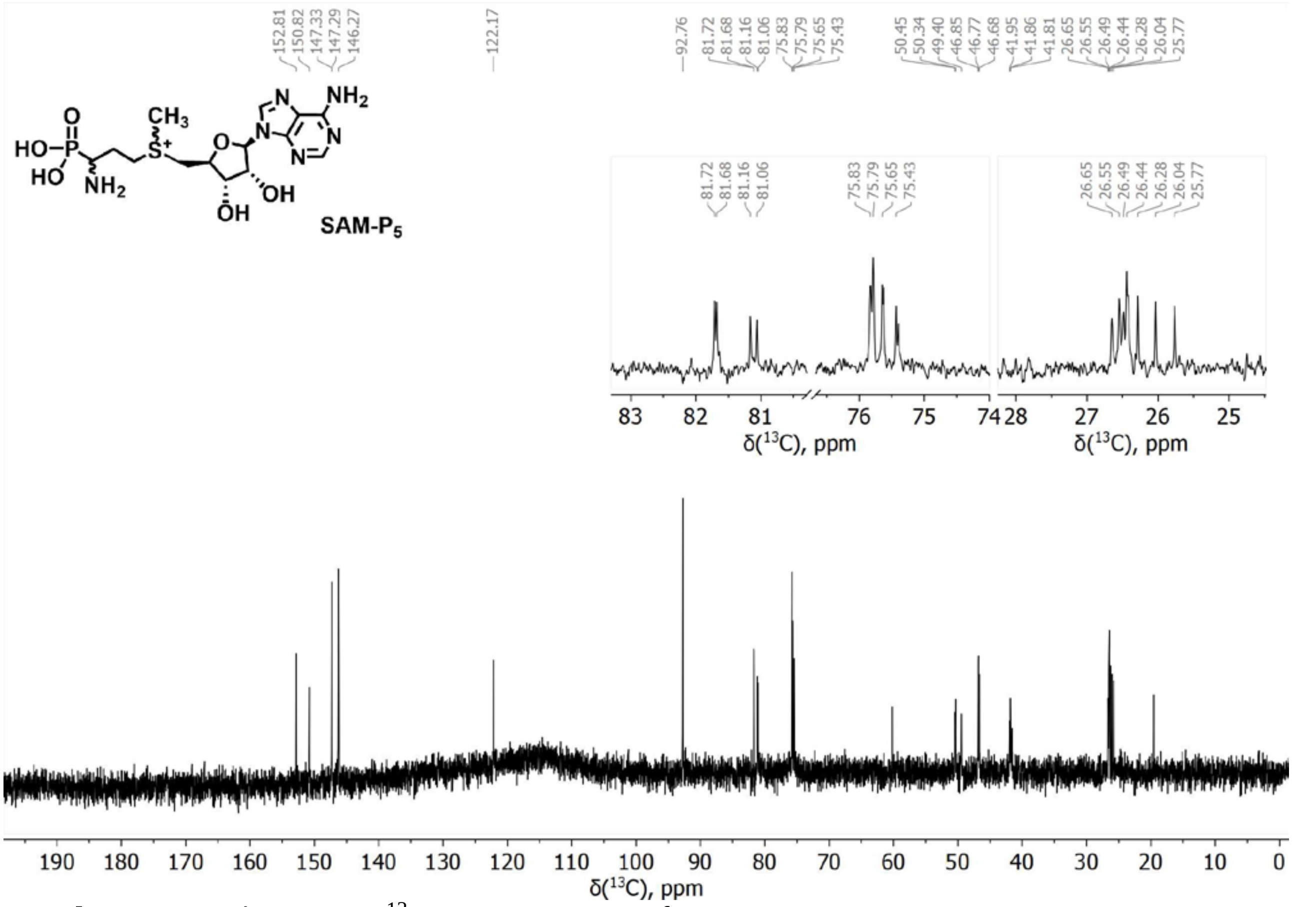

Supplementary Figure S62. ^{13}C NMR spectrum of *rac*-SAM-P_5

^{13}C NMR (151 MHz, D_2O) δ = 152.8, 150.8, 147.3 (d, *J*=5.3), 146.3, 122.2, 92.8, 81.7 (d, *J*=5.5), 81.1 (d, *J*=15.4), 76.0 – 75.3 (m), 50.6 – 49.3 (m), 47.0 – 46.6 (m), 42.1 – 41.5 (m), 26.8 – 25.7 (m).

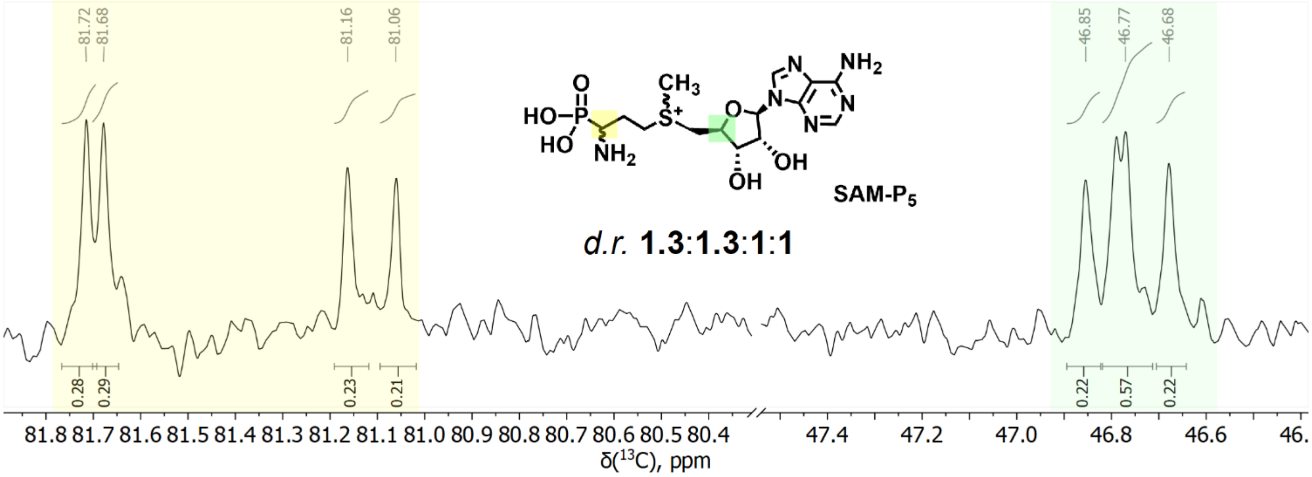

Supplementary Figure S63. The fragment of ^{13}C NMR spectrum of *rac*-SAM-P_5. The diastereomeric ratio (*d.r.*) of various stereoisomers 1.3:1.3:1:1.

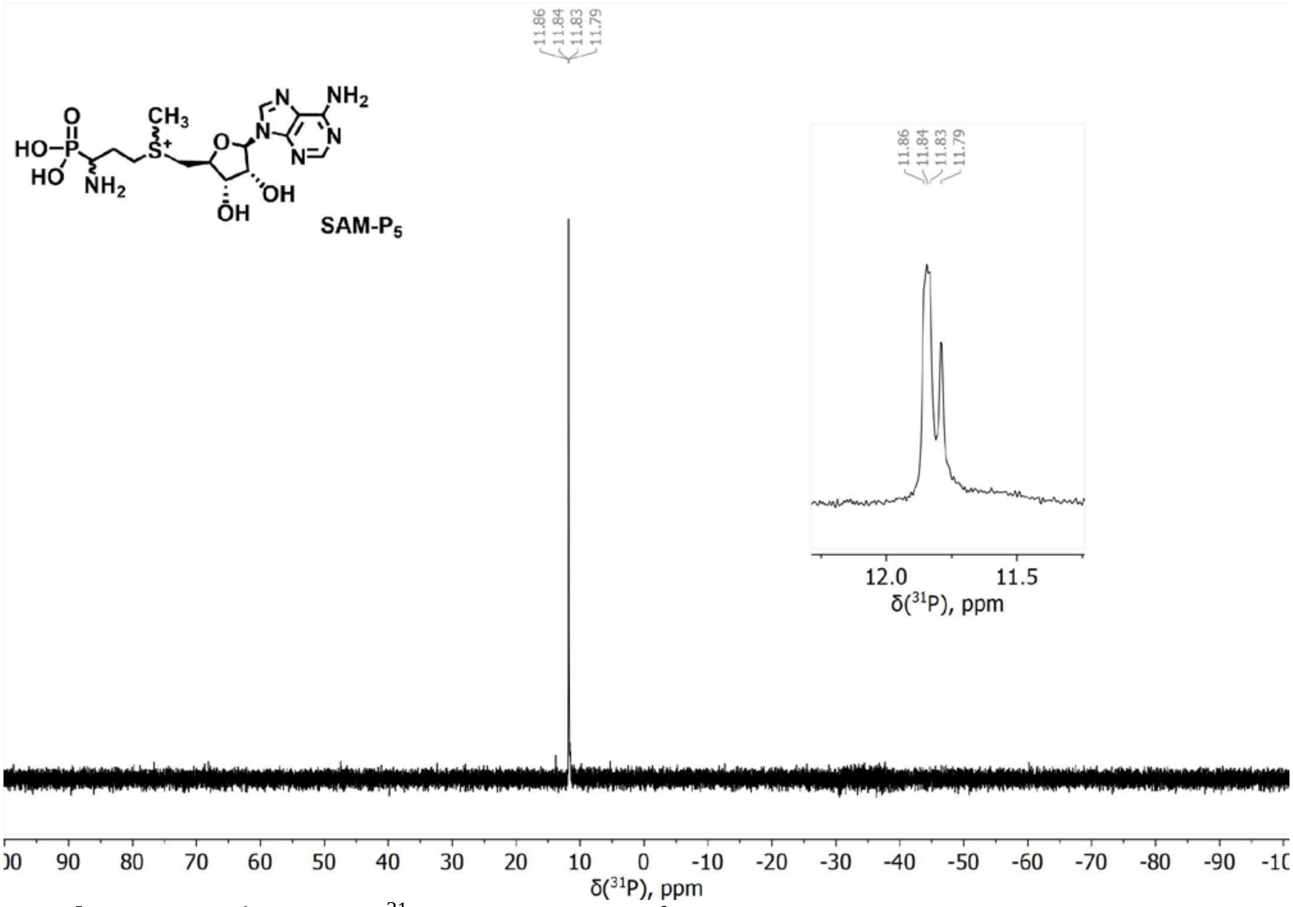

Supplementary Figure S64. ^{31}P NMR spectrum of *rac*-SAM-P_5

^{31}P NMR (243 MHz, D_2O) δ = 11.9 – 11.7 (m).

5 HR-ESI mass spectra of small molecules

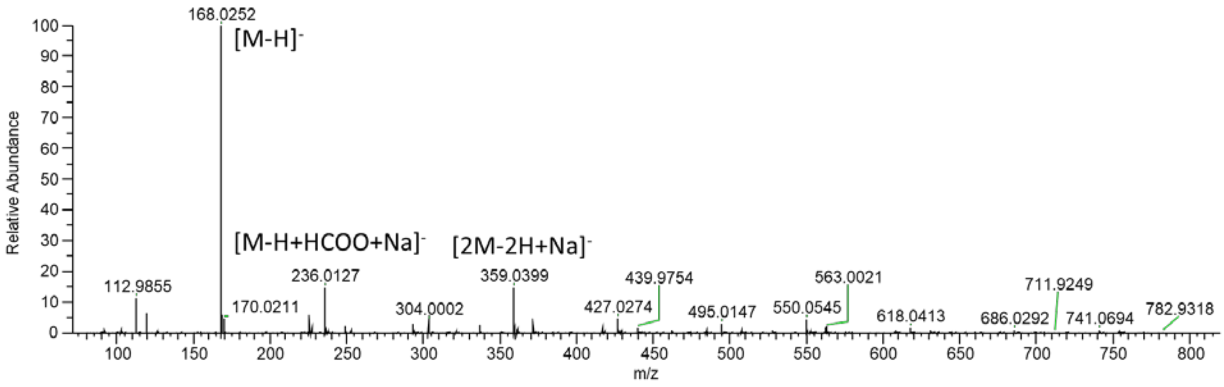

Supplementary Figure S65. Mass-spectra for *rac*-Met-P_H (negative ions). Expected mass for $C_4H_{11}NO_2PS^-$ = 168.0254 $[M-H]^-$, found: 168.0252

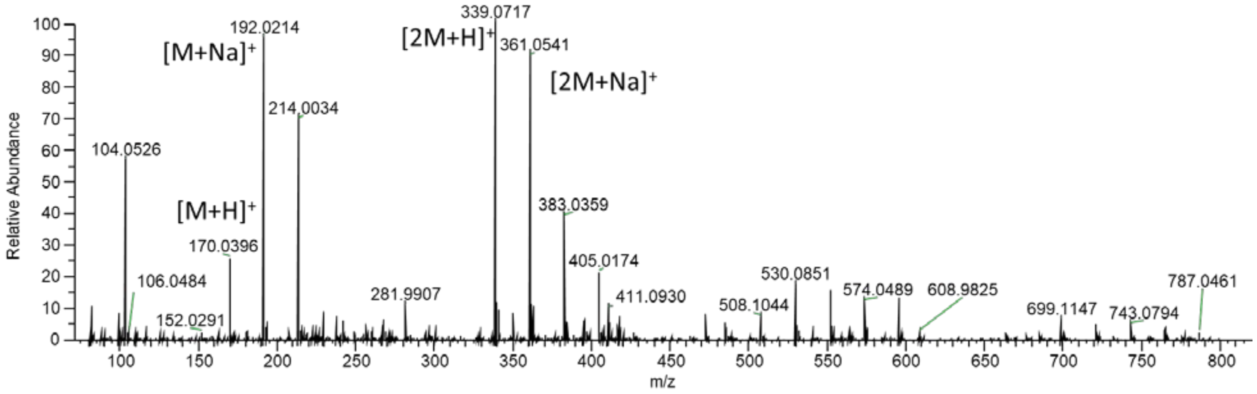

Supplementary Figure S66. Mass-spectra for *rac*-Met-P_H (positive ions). Expected mass for $C_4H_{13}NO_2PS^+$ = 170.0399 $[M+H]^+$, found: 170.0396

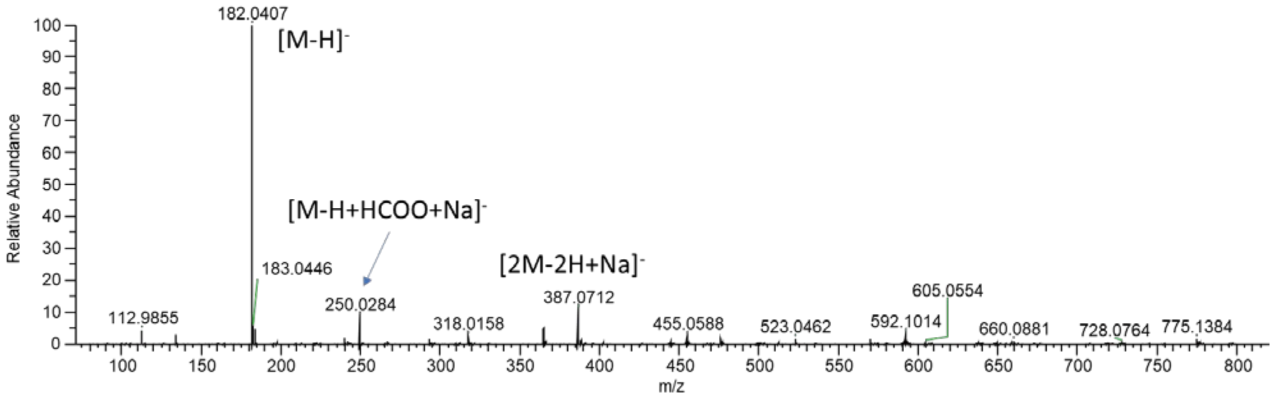

Supplementary Figure S67. Mass-spectra for α-CH_3-Met-P_H (negative ions). Expected mass for $C_5H_{13}NO_2PS^-$ = 182.041 [M-H]$^-$, found: 182.0407

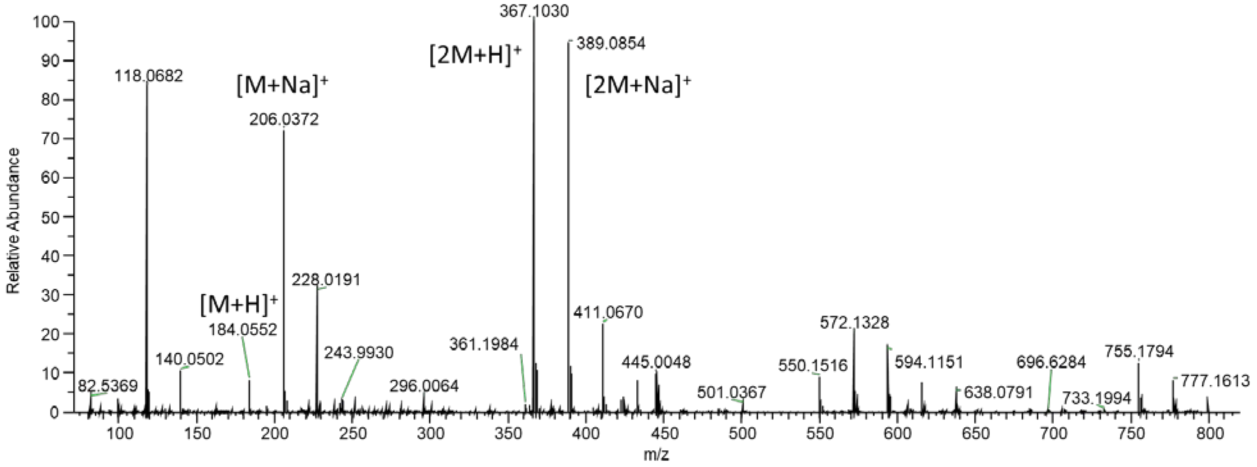

Supplementary Figure S68. Mass-spectra for α-CH_3-Met-P_H (positive ions). Expected mass for $C_5H_{15}NO_2PS^+$ = 184.0556 [M+H]$^+$, found: 184.0552

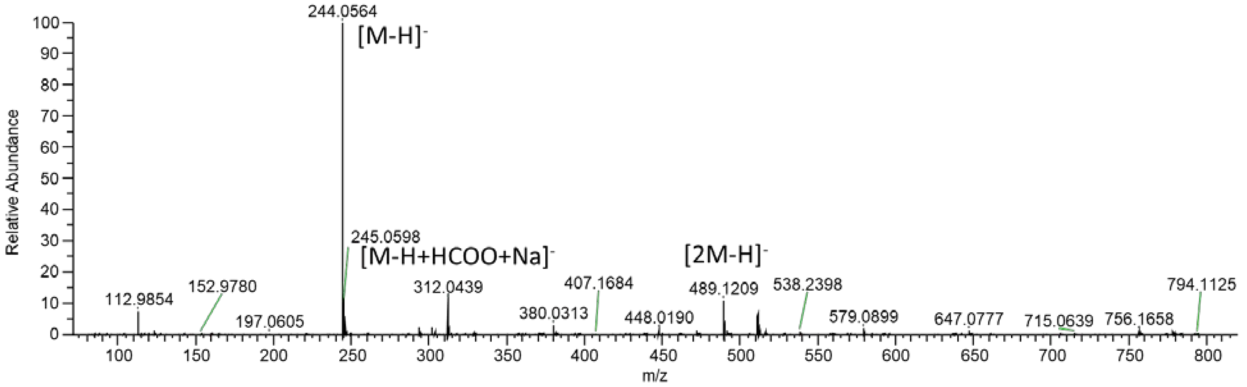

Supplementary Figure S69. Mass-spectra for Bn-S-Hcy-P_H (negative ions). Expected mass for $C_{10}H_{17}NO_2PS^-$ = 244.0567 [M-H]$^-$, found: 244.0564

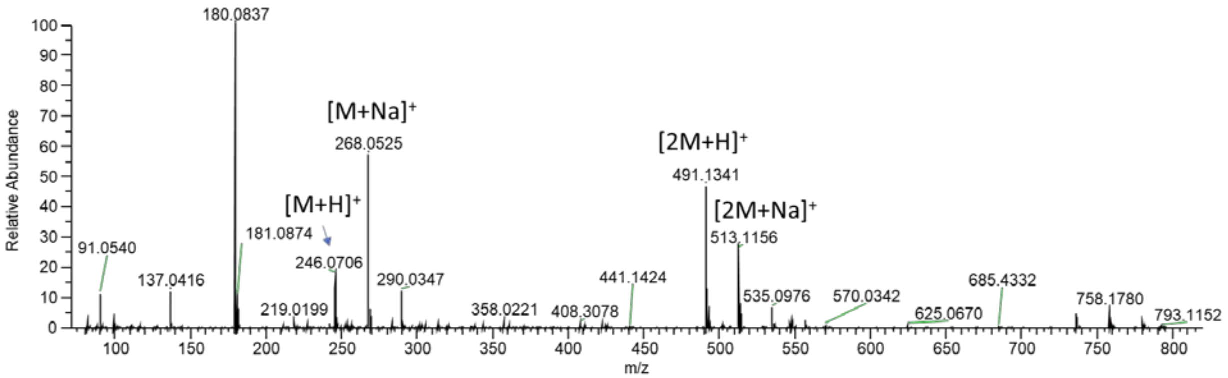

Supplementary Figure S70. Mass-spectra for Bn-S-Hcy-P$_H$ (positive ions). Expected mass for $C_{10}H_{19}NO_2PS^+$ = 246.0712 [M+H]$^+$, found: 246.0706

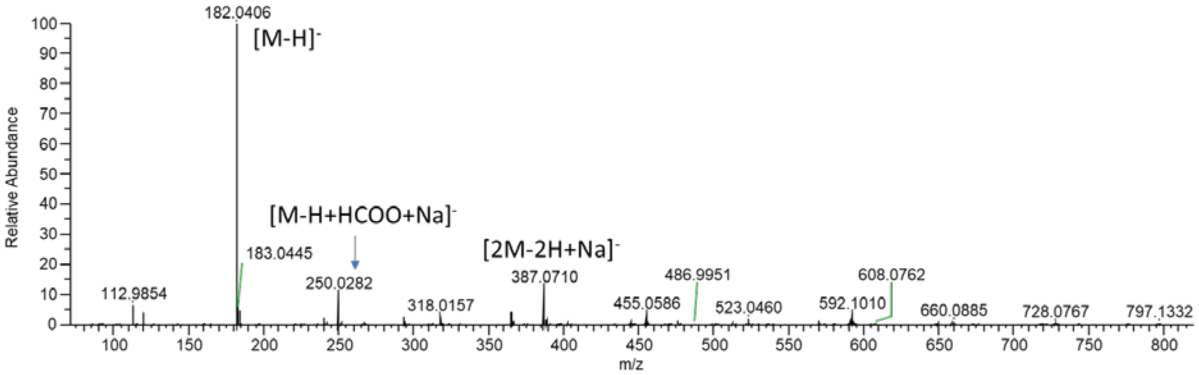

Supplementary Figure S71. Mass-spectra for Ethionine-P$_H$ (negative ions). Expected mass for $C_5H_{13}NO_2PS^-$ = 182.041 [M-H]$^-$, found: 182.0406

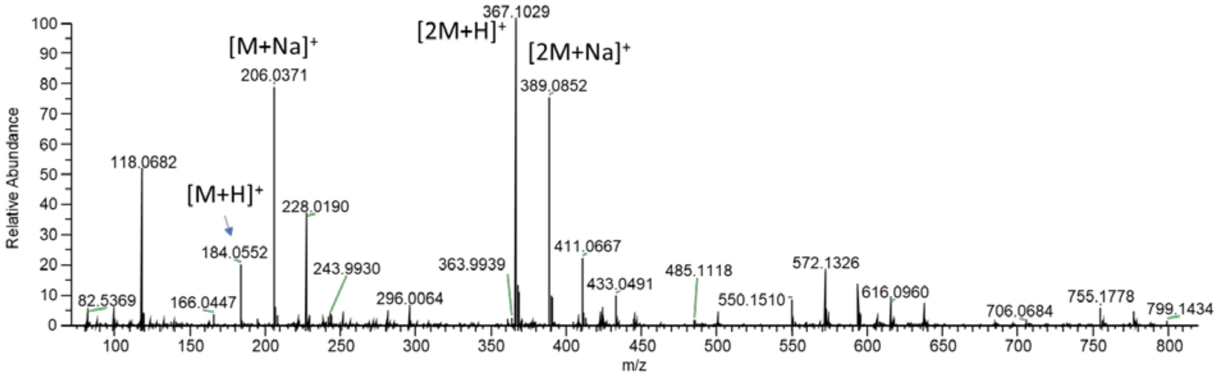

Supplementary Figure S72. Mass-spectra for Ethionine-P$_H$ (positive ions). Expected mass for $C_5H_{15}NO_2PS^+$ = 184.0556 [M+H]$^+$, found: 184.0552

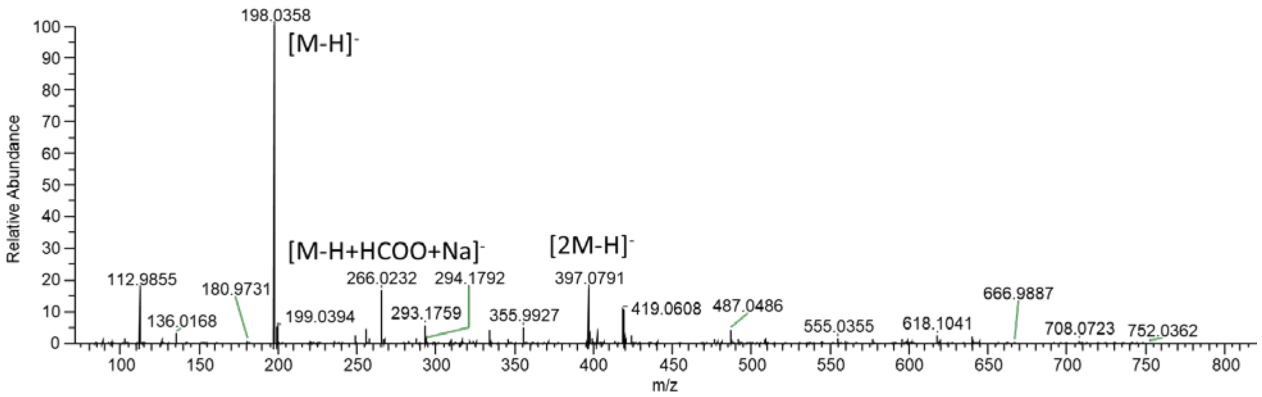

Supplementary Figure S73. Mass-spectra for Ethionine-P_5 (negative ions). Expected mass for $C_5H_{13}NO_3PS^-$ = 198.0359 $[M-H]^-$, found: 198.0358

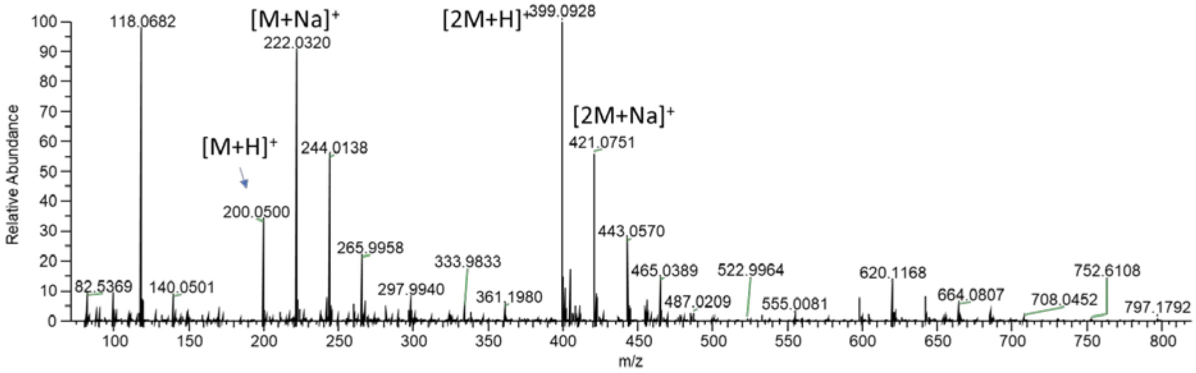

Supplementary Figure S74. Mass-spectra for Ethionine-P_5 (positive ions). Expected mass for $C_5H_{15}NO_3PS^+$ = 200.0505 $[M+H]^+$, found: 200.05

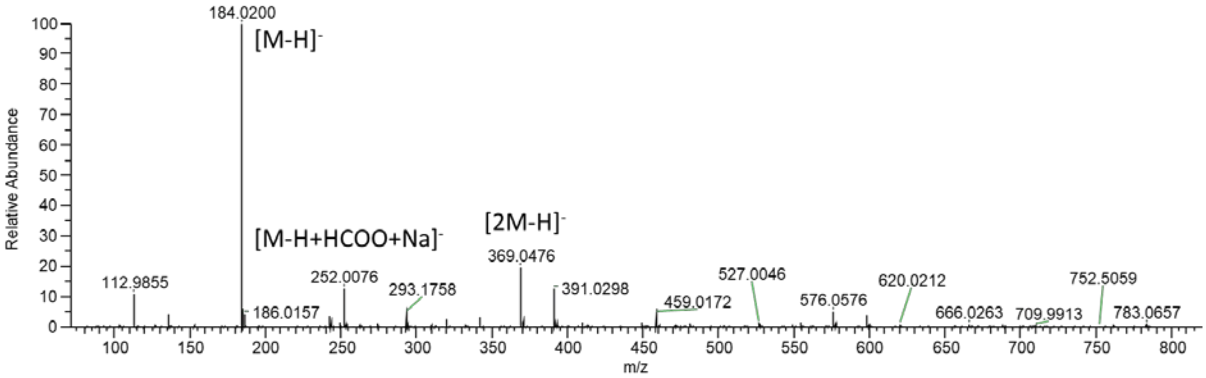

Supplementary Figure S75. Mass-spectra for Met-P_5 (negative ions). Expected mass for $C_4H_{11}NO_3PS^-$ = 184.0203 $[M-H]^-$, found: 184.02

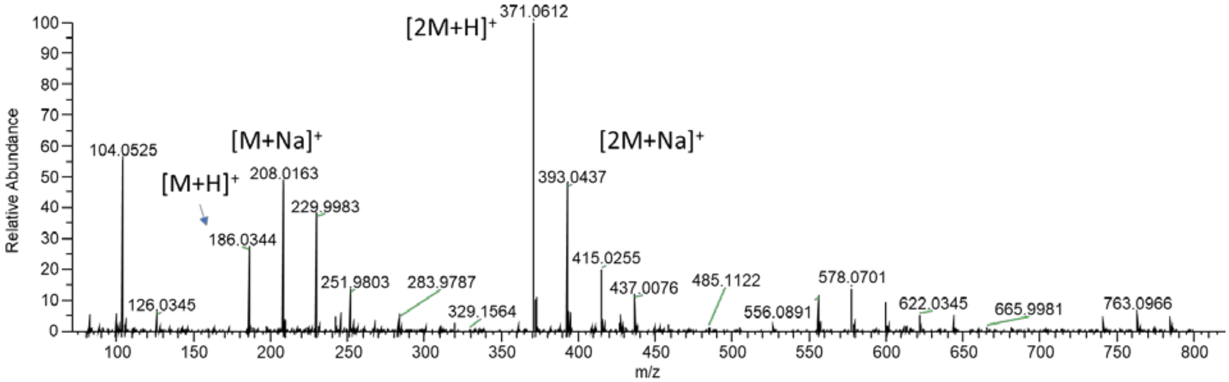

Supplementary Figure S76. Mass-spectra for Met-P_5 (positive ions). Expected mass for $C_4H_{13}NO_3PS^+$ = 186.0348 $[M+H]^+$, found: 186.0344

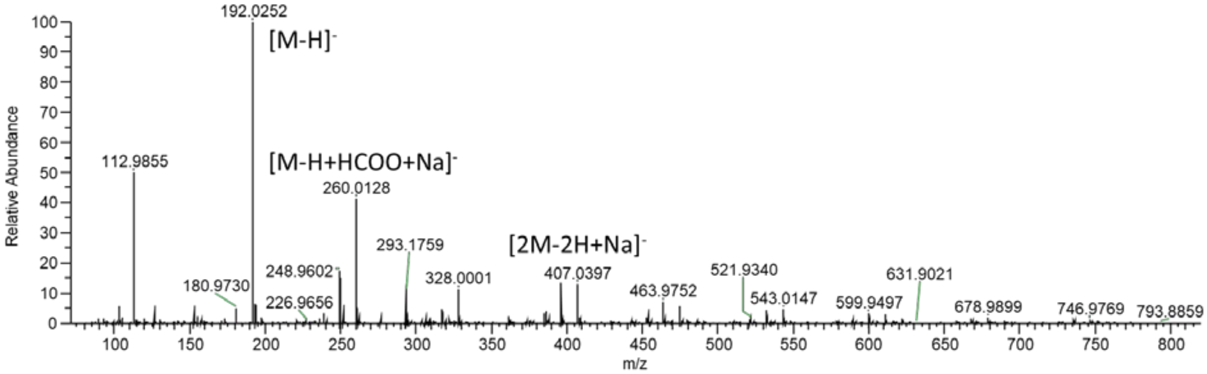

Supplementary Figure S77. Mass-spectra for Pro-S-Hcy-P_H (negative ions). Expected mass for $C_6H_{11}NO_2PS^-$ = 192.0254 $[M-H]^-$, found: 192.0252

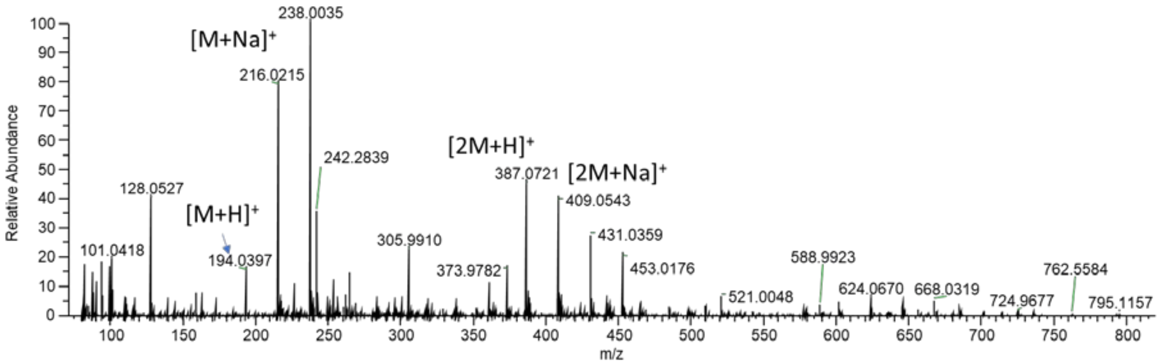

Supplementary Figure S78. Mass-spectra for Pro-S-Hcy-P_H (positive ions). Expected mass for $C_6H_{13}NO_2PS^+$ = 194.0399 $[M+H]^+$, found: 194.0397

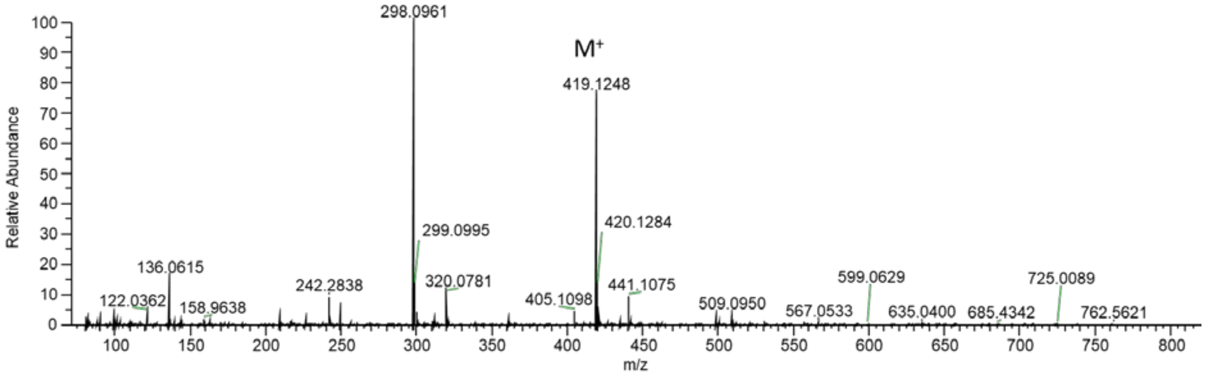

Supplementary Figure S79. Mass-spectra for *rac*-SAM-P_H (positive ions). Expected mass for $C_{14}H_{24}N_6O_5PS^+$ = 419.1261 M^+, found: 419.1248

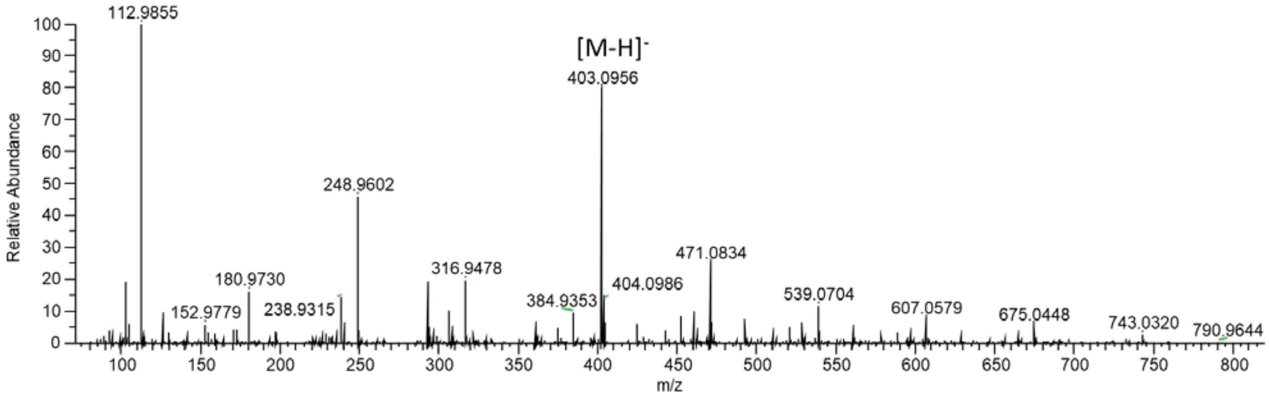

Supplementary Figure S80. Mass-spectra for *rac*-SAH-P_H (negative ions). Expected mass for $C_{13}H_{20}N_6O_5PS^-$ = 403.0959 $[M-H]^-$, found: 403.0956

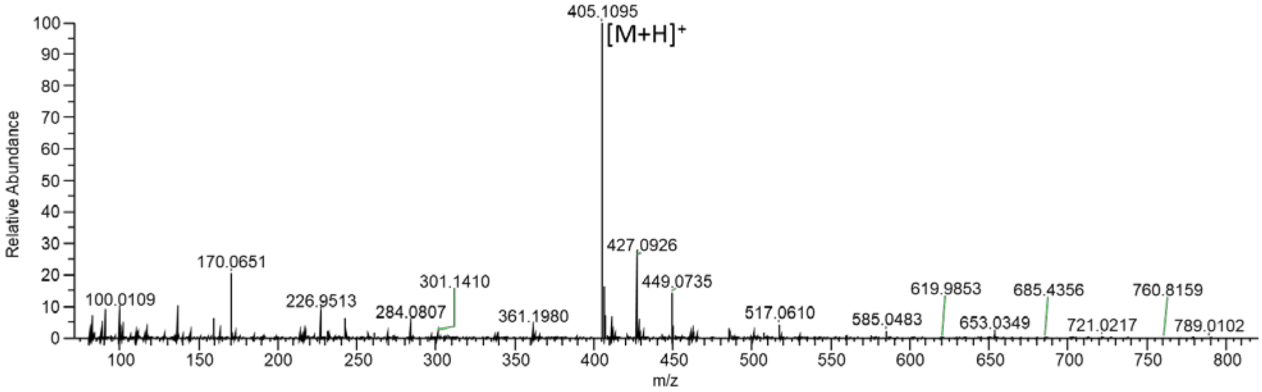

Supplementary Figure S81. Mass-spectra for *rac*-SAH-P_H (positive ions). Expected mass for $C_{13}H_{22}N_6O_5PS^+$ = 405.1105 $[M+H]^+$, found: 405.1095

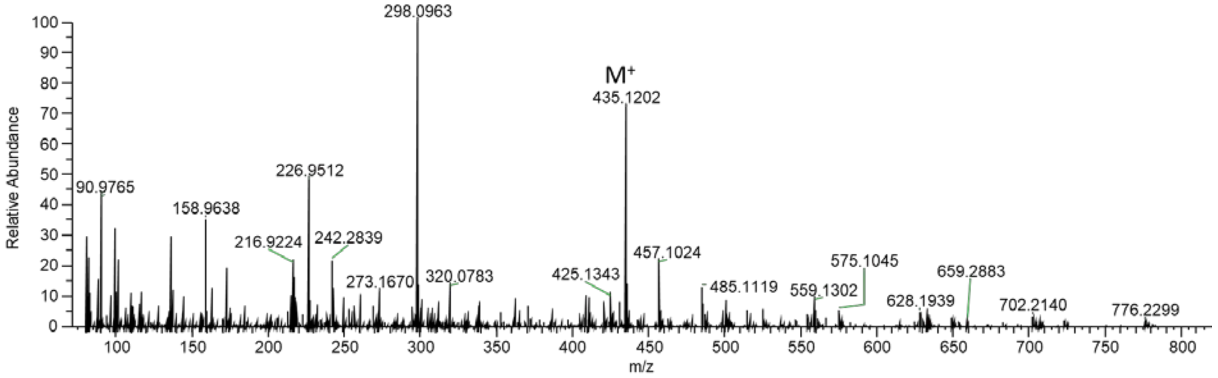

Supplementary Figure S82. Mass-spectra for SAM-P_5 (positive ions). Expected mass for $C_{14}H_{24}N_6O_6PS^+$ = 435.121 M^+, found: 435.1202

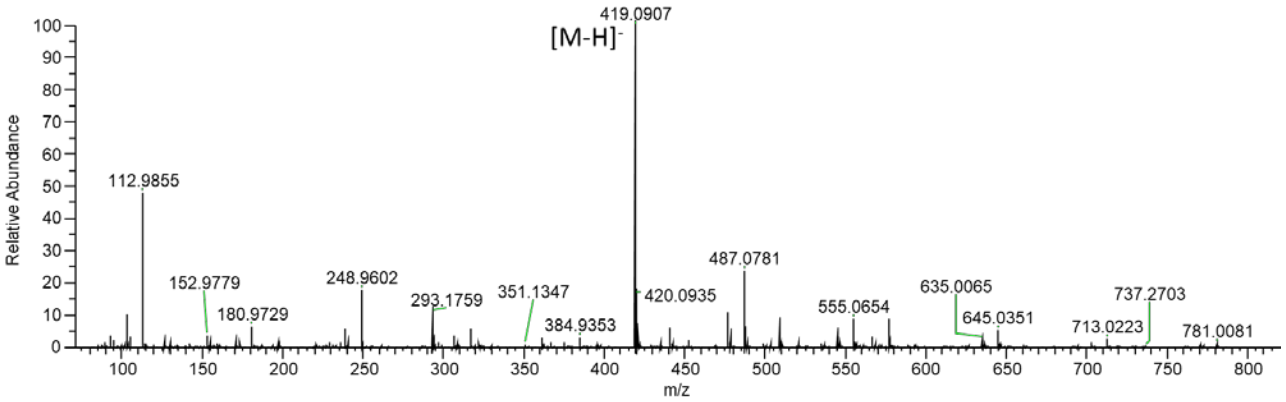

Supplementary Figure S83. Mass-spectra for SAH-P$_5$ (negative ions). Expected mass for $C_{13}H_{20}N_6O_6PS^-$ = 419.0908 [M-H]$^-$, found: 419.0907

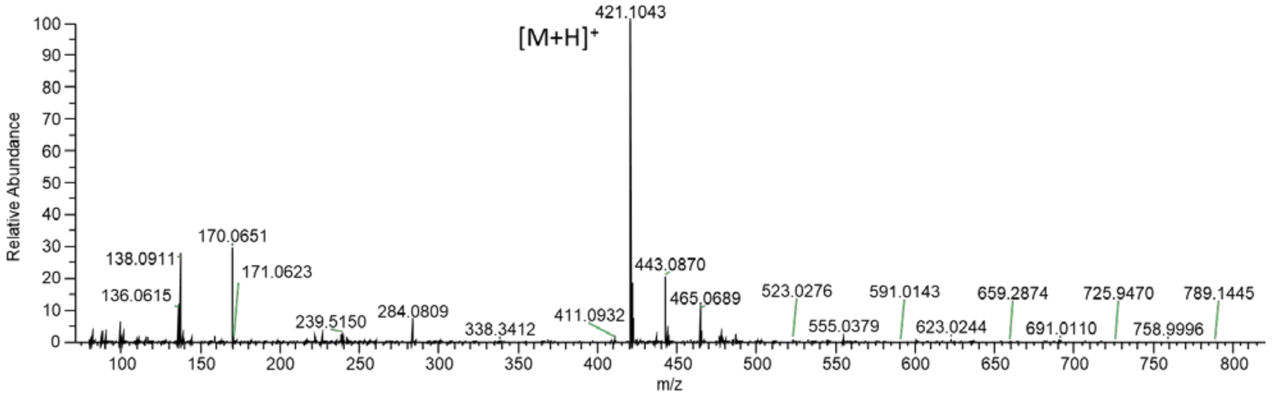

Supplementary Figure S84. Mass-spectra for SAH-P$_5$ (positive ions). Expected mass for $C_{13}H_{22}N_6O_6PS^+$ = 421.1054 [M+H]$^+$, found: 421.1043

6 References

Kitagawa, M., Ara, T., Arifuzzaman, M., Ioka-Nakamichi, T., Inamoto, E., Toyonaga, H., & Mori, H. (2006). Complete set of ORF clones of Escherichia coli ASKA library (A Complete Set of E. coli K-12 ORF Archive): Unique Resources for Biological Research. *DNA Research*, *12*(5), 291–299. https://doi.org/10.1093/dnares/dsi012

Mariasina, S. S., Chang, C. F., Petrova, O. A., Efimov, S. V., Klochkov, V. V., Kechko, O. I., Mitkevich, V. A., Sergiev, P. V., Dontsova, O. A., & Polshakov, V. I. (2020). Williams–Beuren syndrome-related methyltransferase WBSCR27: cofactor binding and cleavage. *FEBS Journal*, *287*(24), 5375–5393. https://doi.org/10.1111/febs.15320

Mariasina, S. S., Petrova, O. A., Osterman, I. A., Sergeeva, O. V., Efimov, S. V., Klochkov, V. V., Sergiev, P. V., Dontsova, O. A., Huang, T., Chang, C.-F., & Polshakov, V. I. (2018). NMR assignments of the WBSCR27 protein related to Williams-Beuren syndrome. *Biomolecular NMR Assignments*, *12*(2), 303–308. https://doi.org/10.1007/s12104-018-9827-2

Tang, Q., Grathwol, C. W., Aslan-Üzel, A. S., Wu, S., Link, A., Pavlidis, I. V., Badenhorst, C. P. S., & Bornscheuer, U. T. (2021). Directed Evolution of a Halide Methyltransferase Enables Biocatalytic Synthesis of Diverse SAM Analogs. *Angewandte Chemie International Edition*, *60*(3), 1524–1527. https://doi.org/10.1002/anie.202013871
